# Recent Advances in C–H Functionalisation through Indirect Hydrogen Atom Transfer †

**DOI:** 10.3390/molecules28166127

**Published:** 2023-08-18

**Authors:** Filip S. Meger, John A. Murphy

**Affiliations:** 1 Institute of Chemical Research of Catalonia (ICIQ), The Barcelona Institute of Science and Technology, 16 Avinguda dels Països Catalans, 43007 Tarragona, Catalonia, Spain; 2 Department of Pure and Applied Chemistry, University of Strathclyde, 295 Cathedral Street, Glasgow G1 1XL, UK

**Keywords:** hydrogen atom transfer, functionalisation, radicals, photoredox, electrochemistry, catalysis

## Abstract

The functionalisation of C–H bonds has been an enormous achievement in synthetic methodology, enabling new retrosynthetic disconnections and affording simple synthetic equivalents for synthons. Hydrogen atom transfer (HAT) is a key method for forming alkyl radicals from C–H substrates. Classic reactions, including the Barton nitrite ester reaction and Hofmann–Löffler–Freytag reaction, among others, provided early examples of HAT. However, recent developments in photoredox catalysis and electrochemistry have made HAT a powerful synthetic tool capable of introducing a wide range of functional groups into C–H bonds. Moreover, greater mechanistic insights into HAT have stimulated the development of increasingly site-selective protocols. Site-selectivity can be achieved through the tuning of electron density at certain C–H bonds using additives, a judicious choice of HAT reagent, and a solvent system. Herein, we describe the latest methods for functionalizing C–H/Si–H/Ge–H bonds using indirect HAT between 2018–2023, as well as a critical discussion of new HAT reagents, mechanistic aspects, substrate scopes, and background contexts of the protocols.

## 1. Introduction

The ability to use C–H bonds as de facto functional handles has streamlined the synthesis of complex organic molecules and changed how chemists approach retrosynthesis [1,2,3,4,5]. Using C–H bonds as functional handles instead of pre-functionalised substrates, the yields’ various benefits include lower step counts in multistep synthesis and an improved atom economy of reactions [3,6,7,8]. Furthermore, the diversity of transformations available to C–H bonds potentially enables access to a wide array of functionality to be introduced into a common core [7,9,10]. Broadly speaking, C–H functionalisation is achieved by generating reactive intermediates from C–H bonds to subsequently harness their reactivity. This can be achieved through organometallic C–H activation [6,11,12,13,14], carbene/nitrene C–H insertion [2,11,15,16,17], enzymatic C–H functionalisation [18], or hydrogen atom transfer (HAT) [11,19,20]. However, site-selective C–H functionalisation is challenging due to the minimal differences between C–H bonds in organic molecules [6,21,22,23]. HAT generates alkyl radicals from C–H bonds through the radical abstraction of hydrogen atoms [19]. Alkyl radicals are highly reactive intermediates that are relatively insensitive to steric crowding and do not form aggregates [24]. Alkyl radicals react chemoselectively with radical traps or couple with other radicals, even with substrates that contain *N*-heterocycles as well as polar and acidic functional groups [20,24,25,26,27,28]. Additionally, HAT processes can be fine-tuned towards specific C–H bonds through choice of HAT reagent, change of solvent, or addition of certain additives [29,30].

Developments in HAT have previously been reviewed [7,19,20,31,32,33,34,35,36,37]. However, due to the rapid pace of protocols published in this field, this review will overlap minimally with those reviews, while works covered previously were omitted unless deemed critical for providing a coherent narrative. Metal hydride-mediated hydrogen atom transfer (MHAT), direct HAT, and indirect HAT mediated by halogen radicals are not covered in this review [31,38,39,40,41,42,43,44,45,46,47,48]. Direct HAT describes HAT where an excited photocatalyst directly carries out HAT [19]. For instance, triplet state ketones [49,50,51,52,53,54,55,56,57,58], decatungstate photocatalysts [48,59,60,61,62,63,64,65,66,67,68,69,70,71], and nitroarenes [72] are direct HAT reagents. Indirect HAT describes protocols where a radical H-atom abstractor is generated in situ [19,73].

### 1.1. HAT Background and Mechanism

**Scheme 1:**
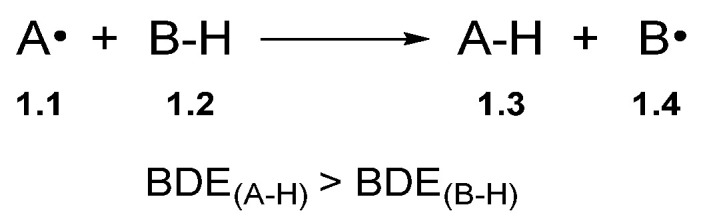
Generic HAT process.

Hydrogen atom transfer is a one-step process that transfers a hydrogen atom (proton and electron) from one species to another (Scheme 1) [74,75]. However, in the context of synthesis, HAT is used for C–H functionalisation by harnessing the reactivity of the alkyl radical with various radical traps [19].

**Scheme 2:**
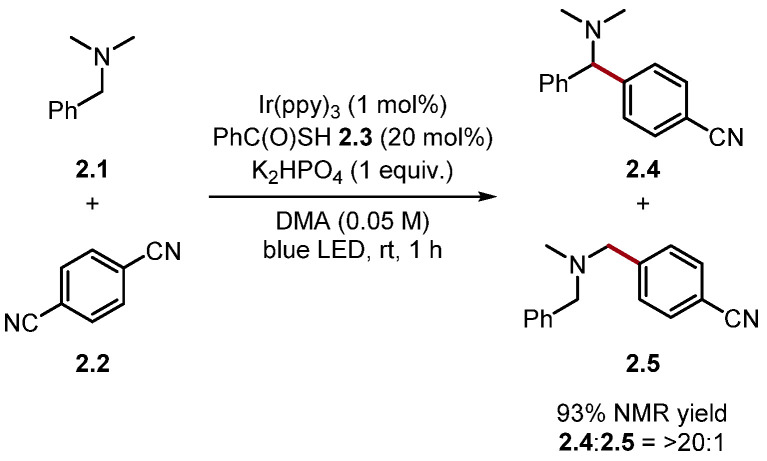
Benzylamine C–H arylation using a thiyl radical formed from thiobenzoic acid.

The bond dissociation energy (BDE) is the key driving force for HAT [76,77,78]. Accordingly, the BDE of A–H in Compound **1.3** (A–H) should be greater than the C–H bond being abstracted (B–H) to favour product formation [75]. Fortunately, BDE values are well-documented in the chemical literature [76,79,80]. BDE values can also be matched to ensure the desired hydrogen atom is abstracted. For example, thiyl radicals undergo HAT with relatively weak C–H bonds to form the corresponding alkyl radical and a thiol [alkyl thiols BDE_S–H_ ≈ 87 kcal/mol] [81]. Hamashima and co-workers developed an arylation of benzylamine **2.1** C(sp^3^)–H bonds, which proceeded through regio- and chemo-selective HAT of the benzylic C(sp^3^)–H using a thiyl radical derived from thiobenzoic acid **2.3** [*N*,*N*-dimethylbenzylamine **2.1** BDE_C–H_ = 84.9 kcal/mol versus thiobenzoic acid **2.3** BDE_S–H_ = 87.4 kcal/mol] (Scheme 2) [82].

**Scheme 3:**
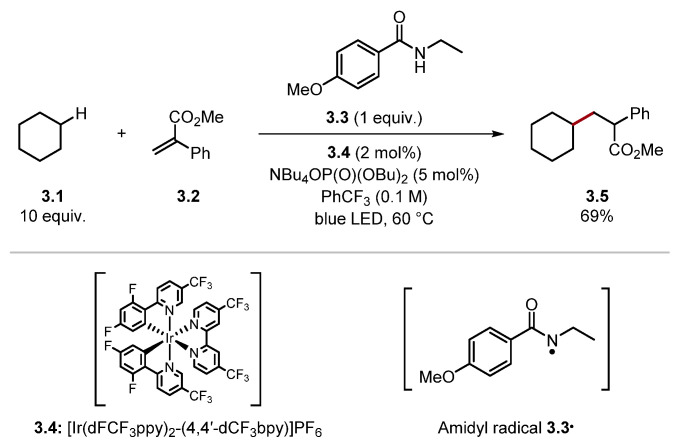
C(sp^3^)–H alkylation using amide **3.3** as a HAT reagent.

Conversely, stronger HAT reagents can be used to abstract unactivated C(sp^3^)–H bonds of alkanes [76]. Knowles and co-workers reported the alkylation of cyclohexane **3.1** through HAT with amidyl radical **3.3•** [amide **3.3** BDE_N–H_ = 107 kcal/mol versus cyclohexane **3.1** BDE_C–H_ = 99.5 kcal/mol] (Scheme 3) [83]. The high BDE value of N–H bonds in amides allows for HAT of unactivated C(sp^3^)–H bonds of alkanes. Hence, BDE values of the HAT reagent and the substrate should be matched carefully to ensure the HAT process is thermodynamically spontaneous and selective [76].

**Scheme 4:**
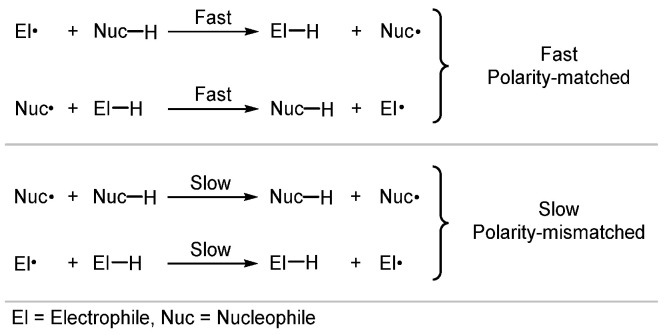
Polarity effects in HAT processes.

Despite radicals being electronically neutral species, electronic factors in transition states of radical reactions greatly influence their rate and selectivity [84,85,86,87]. In this context, polar effects describe the effect the charge transfer has on the activation energy, and HAT is strongly influenced by such electronic factors. Usually, polarity-matched HAT describes the tendency of electrophilic HAT reagents to abstract electron-rich (“hydridic”) hydrogen atoms and nucleophilic HAT reagents to abstract electron-poor (“protic”) hydrogen atoms (Scheme 4) [84,88].

**Scheme 5:**
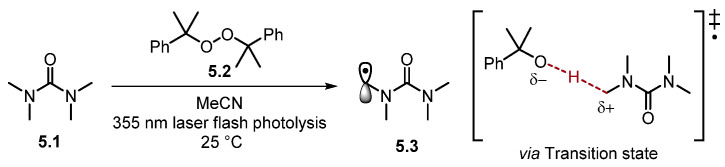
Transition state (‡) of HAT with cumyloxyl radical.

Most HAT reagents are electrophilic and selectively abstract electron-rich hydrogen atoms [30]. As a result, HAT normally occurs adjacent to an electron-donating group (EDG) or another stabilizing functional group [19,48]. Bietti and co-workers have extensively studied the reaction rates of HAT [29,30,80,89,90,91,92,93,94,95,96,97,98,99,100,101,102]. Recently, Bietti studied the rates of HAT for saturated *N*-containing heterocycles and tetramethyl urea **5.1** using dicumyl peroxide **5.2** (Scheme 5) [89].

The HAT transition state can be described as developing a partial positive charge at the C–atom, along with a partial negative charge on the abstracting radical (cumyloxyl radical in this case) [29,77,78]. Functional groups such as amides stabilise the partial positive charge on the incipient radical atom through an orbital overlap of the σ* of the α-C–H (developing SOMO) with a heteroatom lone pair or a π-system [89]. This transition state model has been probed through experimental observations, Hammett plot analysis, and computational studies [78,88,90,103]. HAT processes are dictated by an electron density at different C–H bonds meaning a change in solvent, an addition of H-bond donor/acceptors, or Brønsted/Lewis-acid/base additives can alter the rates of HAT [29,30]. For instance, strong H-bonding solvents [such as hexafluoroisopropanol (HFIP)] are used to supress undesired HAT adjacent to H-bond acceptors (e.g., heteroatoms) [72,92,104,105,106]. However, H-bonding solvents are also known to accelerate HAT at cyclohexane and 1,4-cyclohexadiene through H-bonding to oxyl-radicals [29,107,108,109].

**Scheme 6:**
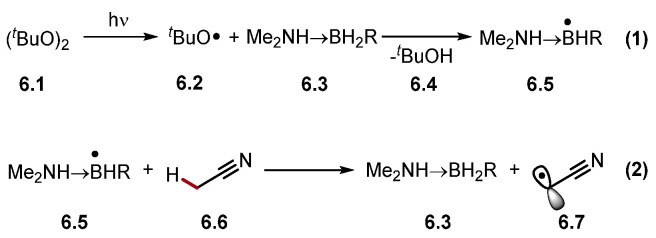
HAT of protic hydrogen atom from MeCN using PRC.

The abstraction of protic hydrogen atoms is difficult as most HAT-capable radicals are inherently electrophilic heteroatom-centred radicals or radical cations [30]. However, the abstraction of protic hydrogens can be accomplished through a polarity reversal catalysis (PRC) [84]. PRC can generate a nucleophilic HAT reagent through an initial polarity-matched HAT step (Scheme 6) [110,111]. For example, electrophilic alkoxyl radical **6.2** reacts with amino boranes **6.3** to form a nucleophilic amine boryl radical **6.5**, which abstracts protic hydrogen atoms selectively, such as acetonitrile **6.6** α-C(sp^3^)–H to generate electrophilic alkyl radical **6.7**.

### 1.2. Indirect HAT

**Scheme 7:**
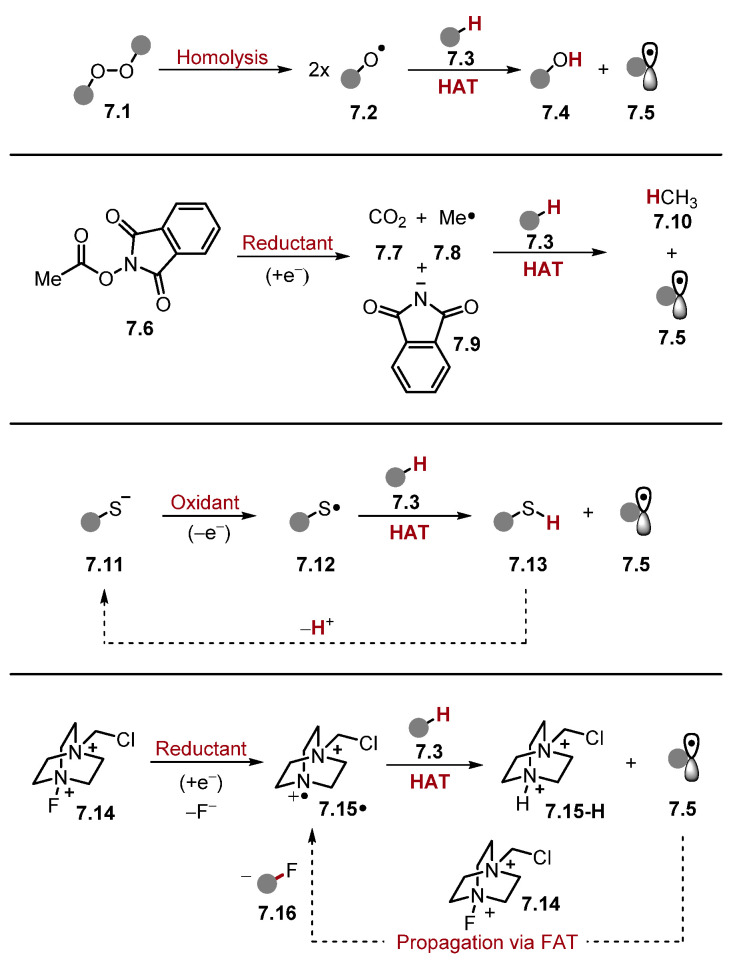
Methods of generating a HAT-capable species in situ.

As mentioned before, indirect HAT describes protocols where a radical H-atom abstractor is generated in situ [19,73]. Radicals capable of HAT are typically formed in the following ways (Scheme 7).

(1) Homolytic/Heterolytic cleavage of a weak bond. The weak O–O bond in peroxides **7.1** undergoes homolysis to form two oxygen-centred radicals **7.2** capable of HAT [*^t^*BuCH_2_O–OCH_2_*^t^*Bu BDE_O–O_ = 36.4 kcal/mol] [79,112]. The peroxide **7.1** can also undergo heterolysis with a reducing agent/acid to form one equivalent of O-centred radical **7.2** [112].

(2) Mesolytic cleavage of a radical ion. For instance, a redox active ester (RAE) **7.6** can be reduced to a radical anion [113,114,115,116]. The radical anion undergoes mesolytic cleavage forming CO_2_ **7.7**, phthalimide anion **7.9**, and a methyl radical **7.8**, which is a competent HAT reagent [117].

(3) Oxidation of a heteroatom or an anion using photoredox catalysis or (less commonly) electrochemistry [118]. For example, a thiolate **7.11** can be oxidised to a thiyl radical **7.12**, which can abstract a hydrogen atom to form a thiol **7.13** and alkyl radical **7.5** [119]. Deprotonation of the thiol **7.13** allows the turnover of the HAT reagent to make the process catalytic.

(4) Radical propagation steps can continuously regenerate the HAT reagent (otherwise known as chain transfer). In the provided example, a fluorine atom transfer (FAT) between alkyl radical **7.5** and Selectfluor **7.14** affords the fluorinated product **7.16** and generates an equivalent of TEDA^2+^**· 7.15·** for further HAT [120]. Notably, chain transfer can also be a contributing pathway in reactions where Methods (1), (2), and (3) are the main pathways with widely varying degrees of chain contribution. In photoredox chemistry, the contribution of the chain transfer to the reaction mechanism can be investigated using quantum-yield measurements or “light/dark experiments” [121]. However, reactions that utilise chain propagation as the major pathway typically use a sub-stoichiometric amount of initiator to initiate the process [122,123,124,125,126].

Each method can form a radical species capable of HAT, therefore facilitating the generation of radicals from corresponding C–H bonds, as well as X–H bonds (where X = heteroatom). Harnessing the high reactivity of radicals allows for a multitude of transformations [28,58,59,76,127,128].

## 2. C–H Functionalisation Using HAT Chemistry

The functionalisation of C–H bonds through radical mechanisms has been a subject of intense research in recent years [19,33,76]. Accessing alkyl radicals can be accomplished through oxidation–deprotonation pathways. However, this approach requires C–H substrates that are easily oxidised substrates and/or requires strongly oxidizing photoredox catalysts [76,129,130,131,132,133]. However, hydrogen atom transfer relies on the abstraction of weak/activated C–H bonds to generate the corresponding alkyl radical [7,19,48].

### 2.1. Nitrogen-Based HAT Reagents

Nitrogen-centred radicals that participate in HAT chemistry are typically highly electrophilic, and their N–H derivatives possess a range of N–H bond strengths [79,134]. However, due to the high BDE of quinuclidine-type species (such as TEDA^2+^-H **7.15-H**) and amide N–H **3.3** and the electrophilic character of their corresponding *N-*centred radicals, these species are used for the abstraction of strong hydridic C–H bonds.

#### 2.1.1. Quinuclidine and DABCO-Style HAT Reagents

**Scheme 8:**
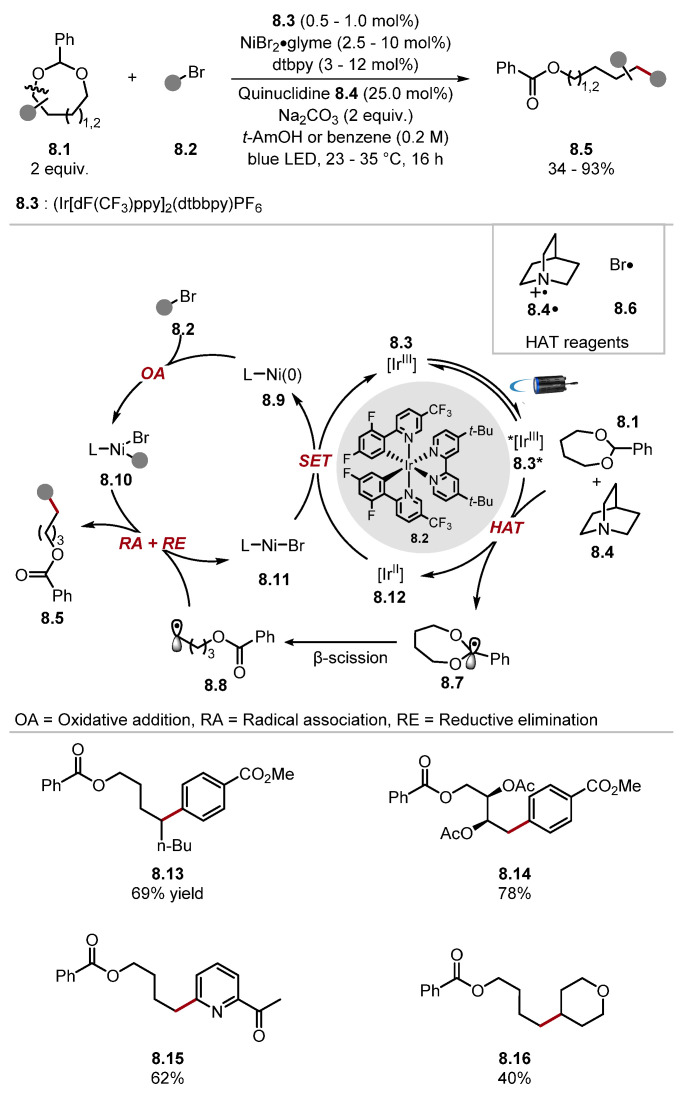
C-C bond formation through C(sp^3^)-O bond scission in cyclic acetals through HAT with quinuclidine.

**Scheme 9:**
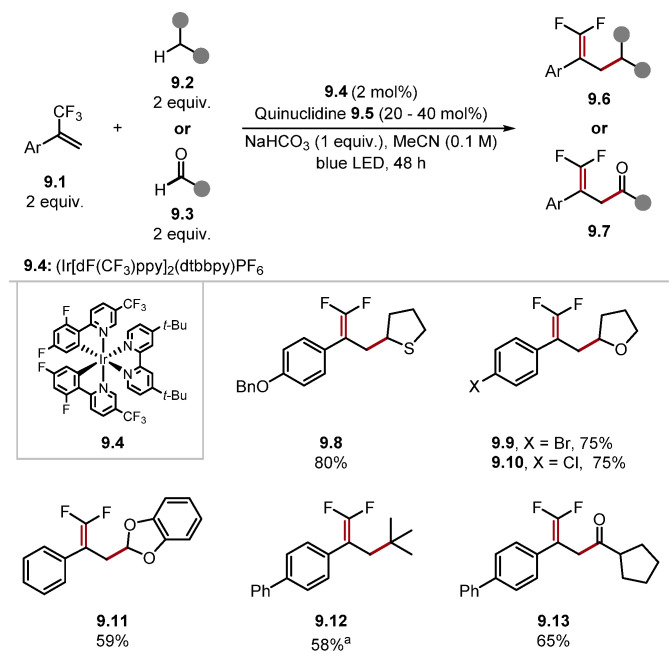
C-H difluoroallylation with photoredox HAT tandem catalysis using quinuclidine. ^a^Product **9.12** was formed from pivaldehyde.

Quinuclidine and DABCO-type HAT reagents are amongst the most popular reagents for HAT (especially in tandem catalytic protocols with photoredox catalysts) [118,135]. They remain a popular option especially for HAT of strong hydridic C–H bonds due to their strong N^+^–H bonds [quinuclidine **8.4-H** BDE_N_^+^_–H_ = 100 kcal/mol] [136]. Additionally, the high electrophilicity of quinuclidine radical cation **8.4·** has been exploited in highly chemo/regio-selective HAT protocols where additives increase electron density at certain hydrogen atoms [137,138]. In some instances, quinuclidine **8.4** has shown a dependence on water. Hence, some protocols use water as a co-solvent/additive [139,140,141]. This is likely caused by water aiding the solubility of the inorganic base (e.g., Na_2_CO_3_ or NaHCO_3_) in the reaction medium, which accelerates the turnover of quinuclidine **8.4** by deprotonating the protonated quinuclidinium intermediate **8.4-H**. Martin recently demonstrated the arylation of C(sp^3^)–O bonds in 7/8-membered cyclic acetals (Scheme 8) [142]. This reaction was initiated by HAT of acetal **8.1** α-C–H with bromine radical **8.6** or quinuclidine radical cation **8.4·** and subsequent β-scission of radical **8.7** to form alkyl radical **8.8**. Alkyl radical **8.8** is trapped by nickel complex **8.10**. The subsequent reductive elimination of a Ni(III) complex delivers product **8.5**. Control experiments showed the reaction proceeded in a 20% lower yield in the absence of quinuclidine **8.4**, suggesting that bromine radical **8.6** is a competing HAT reagent. Halogen radicals (such as Cl**·** and Br**·**) can form through photolysis of metal halide bonds triggered by ligand-to-metal charge transfer (LMCT) [31,143,144,145,146]. The general protocol displayed an excellent functional group tolerance with functionalised acetal rings, ketones and pyridines, and heterocycles reacting well (see products **8.13**–**8.16**). A vinyl bromide and an alkyl bromide (product **8.16**) were also competent electrophilic coupling partners. In 2021, Wang developed a general difluoroallylation protocol using photoredox and HAT tandem catalysis (Scheme 9) [147]. Quinuclidine **9.5** was the optimal HAT reagent for this protocol, as found in preceding literature using identical C–H substrates [137,148]. Alkyl radicals were trapped with 2-trifluoromethylstyrenes **9.1** affording *gem*-difluoroalkene products **9.6** or **9.7**.

**Scheme 10:**
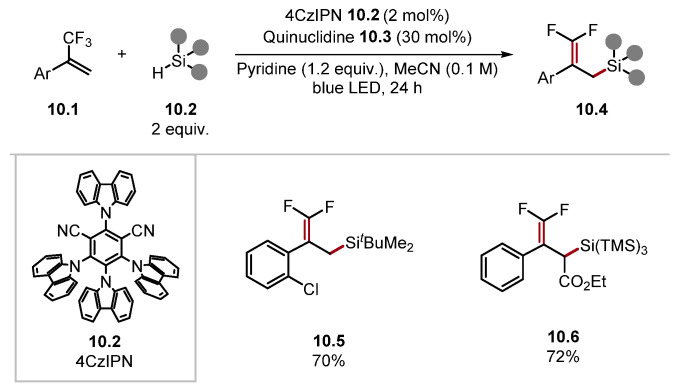
Si-H difluoroallylation with photoredox HAT tandem catalysis using quinuclidine.

**Scheme 11:**
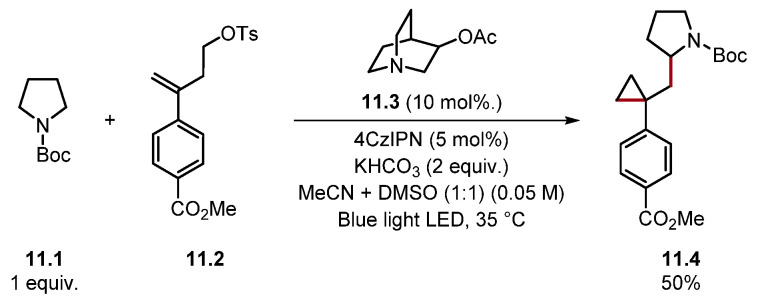
Radical/polar annulation reaction (RPAR) forming a cyclopropyl product **11.4** proceeding through HAT with 3-acetoxyquinuclidine.

This protocol displayed an excellent functional group tolerance and was able to utilise numerous C–H substrates as radical precursors. For instance, in addition to amides and carbamates, thioether, ether and acetal products **9.8**–**9.11** were prepared, while alkyl aldehydes formed ketone products, such as **9.13**. The method did not tolerate aryl aldehydes or alkyl aldehydes containing benzene rings. However, it is worth noting that acetonitrile was the solvent. Solvent effects are important in HAT processes, and the HAT of formyl C–H bonds is known to proceed more efficiently in less-polar solvents such as dioxane or isooctane [98,148]. Notably, alkyl aldehydes with a tertiary alkyl group adjacent to the aldehyde afforded decarbonylated products, as seen in product **9.12**, which was formed from pivaldehyde. This is due to the fast rate of decarbonylation of the corresponding acyl radicals [149]. The method was also highly tolerant of various functional groups on the styrene, and the method was showcased on numerous pharmaceuticals. In 2022, Jing used a similar protocol for hydrosilane **10.2** Si–H difluoroallylation-forming products **10.4** (Scheme 10) [150]. Quinuclidine radical cation **10.3·** is known to abstract hydrogen from Si–H bonds [Et_3_Si–H BDE_Si–H_ = 95.1 kcal/mol versus quinuclidine **10.3** BDE_N_^+^_–H_ = 100 kcal/mol] [79,136,151]. In 2018, Molander reported an example of a radical/polar annulation reaction (RPAR) to form a cyclopropyl product **11.4** proceeding through HAT with 3-acetoxyquinuclidine **11.3** (Scheme 11) [152]. The product was obtained in a moderate yield. However, this could probably be improved by using a photocatalyst, which is stronger in its reduced form (PC**^•^**^−^) as benzyl radicals are known to be reduced slowly by 4CzIPN**^•^**^−^ [153].

**Scheme 12:**
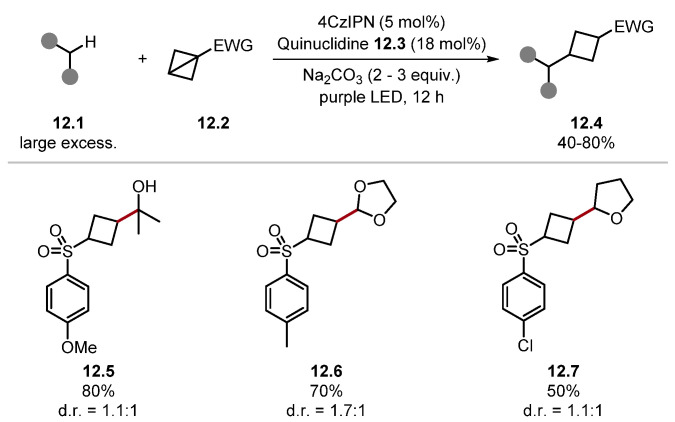
α-Oxyalkyl C(sp^3^)–H cyclobutylation via photoredox HAT tandem catalysis with quinuclidine.

In 2022, Xia used quinuclidine **12.3** for cyclobutylation of α-oxy C(sp^3^)–H bonds using a photoredox HAT tandem catalysis manifold (Scheme 12) [154]. An α-oxyalkyl radical, formed through HAT with a quinuclidine radical cation, attacks electron-poor bicyclo [1.1.0]butane **12.2** to form the functionalised cyclobutane product **12.4** after the reduction and protonation. Alcohols **12.5**, acetals **12.6**, and ethers **12.7** were formed from the appropriate substrates. However, *N*-Boc pyrrolidine, secondary amines, and 1,4-dioxane were unsuccessful. The derivatisation of aldehydes, amides, and thioethers was not attempted.

**Scheme 13:**
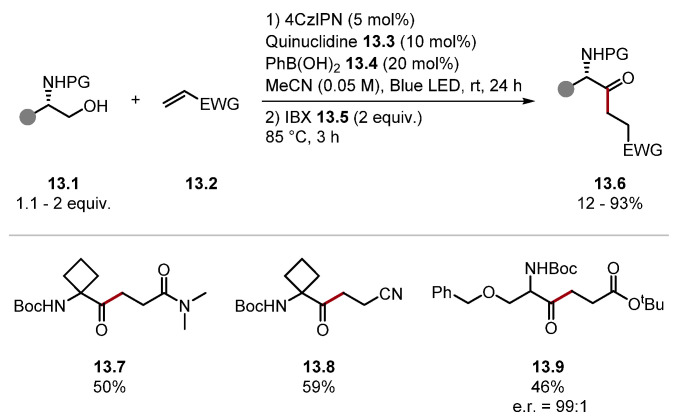
Alcohol α-C(sp^3^)–H alkylation using quinuclidine for HAT provides ketones upon oxidation with IBX.

As mentioned before, polar effects are important in HAT chemistry (Section 1.1). These effects make it possible to promote, or deter, HAT processes by either increasing or decreasing electron density at specific H atoms, respectively. Selectivity has been achieved by adding H-bonding and Brønsted/Lewis acid/base additives [137,138,139,155,156]. Methods for promoting H-atom abstractions at alcohol α-C–H bonds are now widespread [138,157,158,159,160]. In 2022, Suárez used phenylboronic acid **13.4** to promote HAT at alcohol α-C–H bonds on protected α-amino alcohols **13.1** with quinuclidine **13.3** (Scheme 13) [161]. This protocol provides access to γ-oxo-δ-amino acids **13.6** after an oxidation step with IBX [162]. Boc was the optimal protecting group for the amine. However, Cbz was also used. The protocol tolerated a wide range of functionality and used various Giese acceptors. For example, an α,β-unsaturated amide formed product **13.7** and acrylonitrile formed product **13.8**; α,β-unsaturated esters and ketones also reacted well. The protocol proceeded in moderate-to-good yields even in the presence of other weak C–H bonds, as seen in product **13.9**. Overall, this method further expands the utility of alcohols with α-C–H bonds as radical precursors and complements the existing methods [155].

**Scheme 14:**
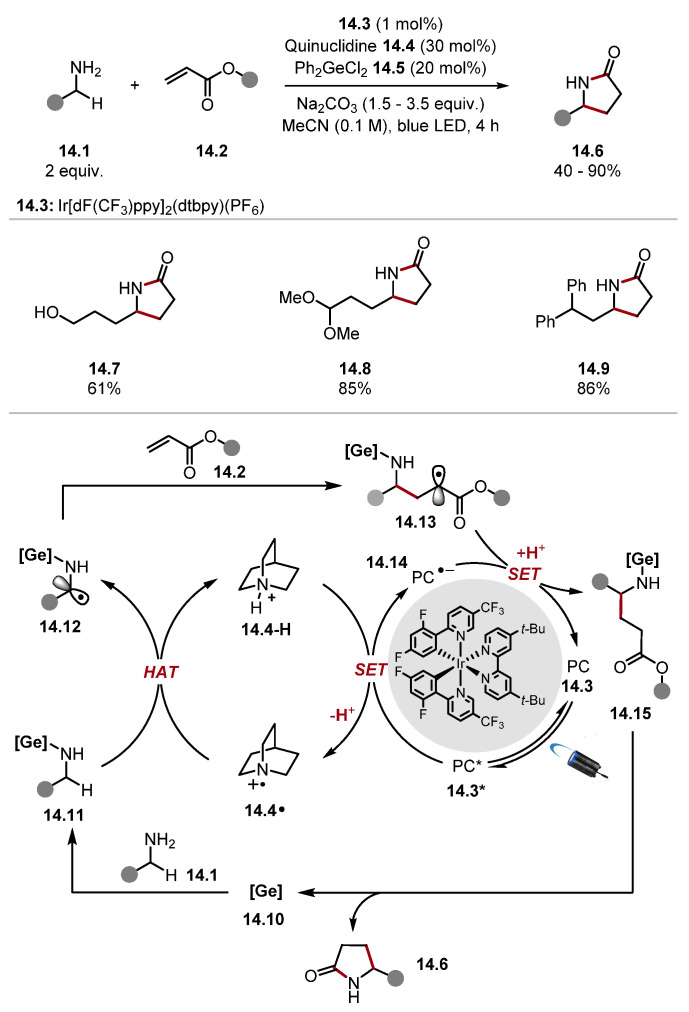
Promotion of HAT at amine α-C–H bonds with a Ge catalyst using quinuclidine.

In contrast to the abundant literature describing the promotion of HAT at alcohol α-C–H bonds, using unprotected amines remains difficult [163]. In 2022, Kanai used a germanium catalyst **14.5** to promote HAT at primary amine **14.1** α-C–H bonds with quinuclidine **14.4** (Scheme 14) [164]. This was used in a Giese protocol with α,β-unsaturated esters **14.2**, which provide lactams **14.6** upon cyclisation. Computational studies showed that the addition of germanium catalyst lowered the BDE of amine α-C–H bonds [ethylamine BDE_α-C–H_ = 94.0 kcal/mol] by 1.3–7.0 kcal/mol depending on whether an aminogermane (neutral 4-coordinate germanium) or aminogermate (anionic 5-coordinate germanate) species is formed in situ [79]. The use of base was vital for the success of the reaction. This effect is probably due to the faster turnover of quinuclidinium **14.4-H**, as well as the prevention of the amine from being protonated, which is known to suppress HAT pathways [29]. This method tolerated a range of functionality, such as alcohols, nitriles, ethers, and acetals (see products **14.7**, **14.8**). The use of germanium catalyst **14.5** supressed HAT at weak benzylic positions and acetal α-C–H bonds [diphenylmethane BDE_benzylic C–H_ = 84.5 kcal/mol and 1,1-dimethoxyethane BDE_C–H_ = 88.2 kcal mol] [79]. These results parallel other methods that use additives to increase electron density at C–H bonds to promote their abstraction even in the presence of weaker bonds through polar effects [137,138,139,155]. The mechanism of the reaction proceeds through the coordination of a germanium catalyst **14.10** to amine **14.1** forming an amino complex **14.11**. The amino complex **14.11** undergoes HAT faster than a primary amine **14.1**. Hence, the amino α-C–H bond in complex **14.11** is abstracted with quinuclidine radical cation **14.4·** to deliver α-aminoalkyl radical **14.12**, which is trapped with a Giese acceptor **14.2** that forms a radical adduct **14.13**. The radical **14.13** is rapidly reduced and protonated to deliver the Giese product **14.15**, which can cyclise to form a lactam **14.6** turning over the germanium catalyst **14.10**.

**Scheme 15:**
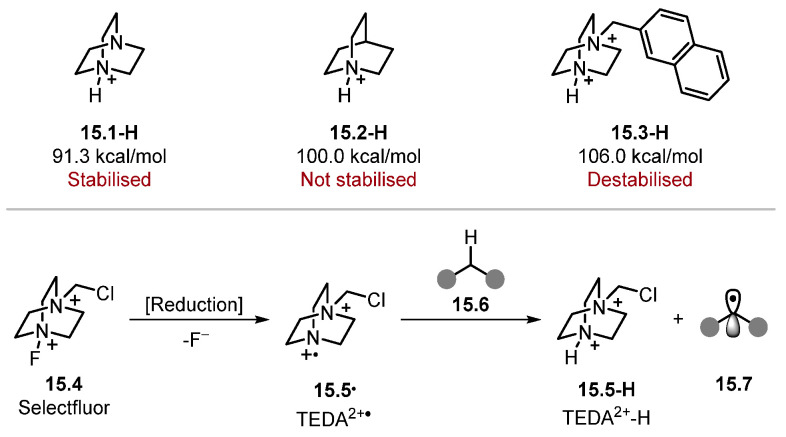
Comparison of DABCO, quinuclidine, and quaternised DABCO.

DABCO **15.1** is different from quinuclidine **15.2** as the radical cation **15.1·** and DABCO–H **15.1-H** are stabilised through a 1-spin-4-non-bonded-electron orbital interaction [165]. As a result, DABCO **15.1** forms weaker N^+^–H bonds compared with quinuclidine **15.2**, which is not stabilised [DABCO **15.1-H** BDE_N+–H_ = 91.3 kcal/mol and quinuclidine–H **15.2-H** BDE_N+–H_ = 100.0 kcal/mol] [136,165]. On that account, a recent trend in DABCO-type HAT reagents has involved removing this stabilizing interaction through the quaternisation of one nitrogen atom, resulting in stronger N^+^–H bonds (for instance, Compound **15.3**) [166]. In several reports, such species are formed through the reduction of Selectfluor **15.4** to form TEDA^2+^**·** radical **15.5·** (Scheme 15) [20,57,167,168,169,170,171]. To the best of our knowledge, no BDE value is known for N^+^–H in TEDA^2+^–H **15.5-H**. However, it is assumed to be around 100 kcal/mol due to its ability to activate alkanes [171].

**Scheme 16:**
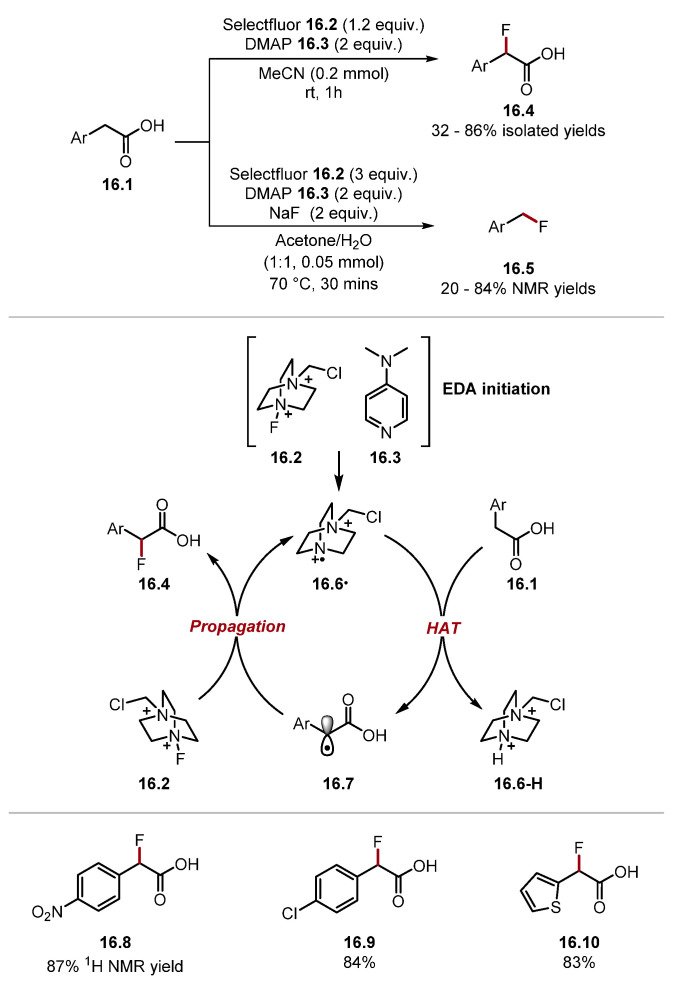
Fluorination of phenylacetic acid α-C–H bonds with Selectfluor.

In 2022, Pieber reported a benzylic C(sp^3^)–H fluorination of phenylacetic acids **16.1** using Selectfluor **16.2** and DMAP **16.3** (Scheme 16) [120].

**Scheme 17:**
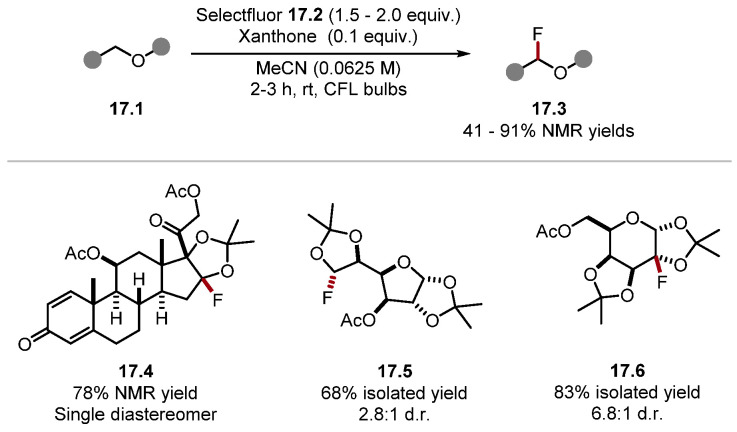
Site-selective fluorination of ketals with Selectfluor.

Conducting the reaction in a mixture of acetone and water led to a decarboxylative fluorination product **16.5**. However, using acetonitrile as a solvent formed fluorinated phenylacetic acid derivatives **16.4**. The initiation step is thought to proceed through an EDA complex between Selectfluor **16.2** and DMAP **16.3**, which forms TEDA^2+^**·** radical **16.6·**. The authors did not perform any experiments elucidating the initiation. However, a similar mechanistic pathway has been previously investigated by Van Humbeck and Baran [126,172]. In addition, Selectfluor **16.2** has a low reduction potential (Selectfluor **16.2** E_1/2_ = +0.33 V vs. SCE in MeCN) [173]. TEDA^2+^**·** radical **16.6·** is a strong HAT reagent and can abstract a benzylic C–H to form benzylic radical **16.7**. The benzylic radical **16.7** can propagate the chain reaction by abstracting a fluorine atom from Selectfluor **16.2**, forming the fluorinated product **16.4** and TEDA^2+^**· 16.6·** [Selectfluor **16.2** BDE_N–F_ = 64.0 kcal/mol versus (fluoromethyl)benzene BDE_C–F_ = 97.6 kcal/mol] [79,174]. In 2020, Lectka and Dudding reported a site-selective fluorination of ketals using Selectfluor **17.2** and xanthone under irradiation with visible light (Scheme 17) [175].

**Scheme 18:**
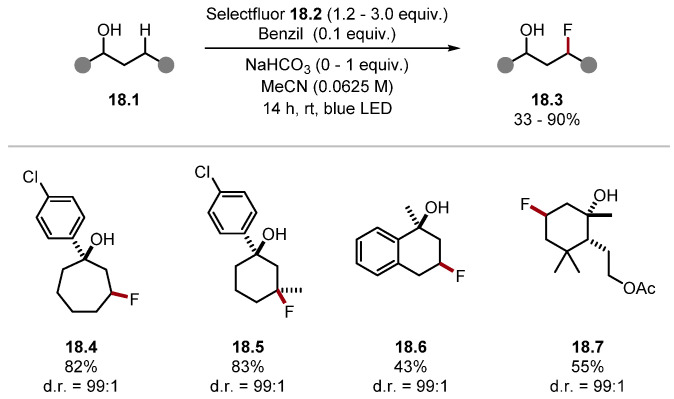
Hydroxy-directed fluorination of C(sp^3^)–H bonds using Selectfluor.

This system worked best on molecules with rigid C–H bonds, most notably on cyclic acetals and ethers. This is likely due to the increased stabilisation of HAT pathways through hyperconjugation in cyclic systems [89]. Numerous polycyclic systems were amenable to the fluorination method. For instance, the precurosor to steroid **17.4** was fluorinated at the ketal α-C(sp^3^)–H bond in a high yield in the presence of a weak allylic C–H bond. Compound **17.5** was also formed in a good yield in the presence of numerous ethereal and acetal C–H bonds. Hence, the site-selectivity observed was not entirely dependent on BDE values as seen with galactose diacetonide **17.6** where five ethereal C–H bonds were present. Computational studies showed that intermolecular interactions in the transition state caused the observed regioselectivity. In a subsequent work, Lectka and Dudding showed carbonyl groups can also direct C(sp^3^)–H fluorination [176]. In 2022, Lectka developed a similar method using alcohols **18.1**, which directed fluorination to the γ-C(sp^3^)–H bonds, forming products **18.3** (Scheme 18) [177]. In addition, 5,6,7-membered rings fluorinated the γ-C(sp^3^)–H bond, as seen in products **18.4** and **18.5**. Notably, the tertiary carbon was prefered to the methylene in product **18.5**. The weaker benzylic position was left unreacted in product **18.6**. In the absence of the hydroxyl group, the fluorination occured on the benzylic C(sp^3^)–H. This demonstrates how the intermolecular interactions in the transition state could alter the outcome of a HAT reaction. Similarly to the group’s previous work, more rigid C–H bonds in cyclic systems reacted in preference to linear ones, as seen in Product **18.7**.

**Scheme 19:**
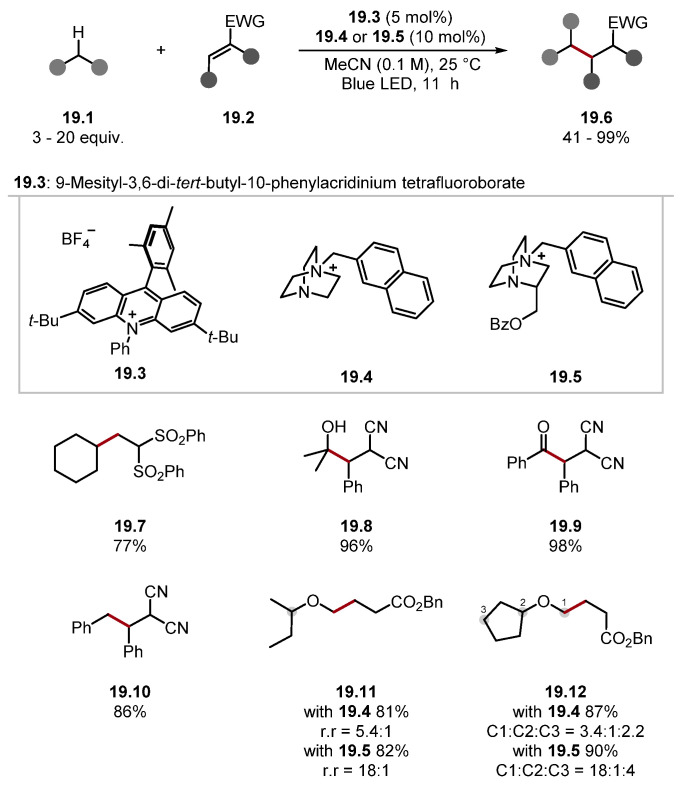
Cationic DABCO-based HAT catalyst used in Giese protocol.

Maruoka developed cationic DABCO-type Catalysts **19.4** and **19.5** for C–H alkylation through a Giese pathway (Scheme 19) [166]. DFT studies showed the DABCO-derived cationic catalysts formed N^+^–H BDE values between 104–109 kcal/mol [**19.4-H** BDE_N+–H_ = 106 kcal/mol]. The low oxidation potential of the reduced form of the acridinium catalyst **19.3** meant only easily reducible alkenes were tolerated, as seen in products **19.7**, **19.8**, and **19.11** (E_1/2_ (Acr **19.3**/Acr+) = −0.56 V vs. SCE in MeCN versus E_1/2_ (**·**CH_2_CO_2_Et/−CH_2_CO_2_Et) = −0.63 V versus SCE in MeCN) [130,178,179,180]. The HAT reagent formed from **19.4** was effective in the alkylation of various C–H substrates forming cycloalkanes **19.7**, alcohols **19.8**, aldehydes **19.9**, toluene **19.10**, and ethers **19.11** and **19.12**. Furthermore, where multiple abstractable C–H bonds were present, the regioselectivity could be refined by using a substituted HAT reagent **19.5**. This effect can be seen on ethers **19.11** and **19.12**. The general method was also showcased on biologically active and complex molecules.

DABCO **20.4** has also been applied to H-atom abstraction from formate anion **20.5** to form a radical anion of CO_2_ **20.10** (CO_2_^•−^) (BDE) [181]. CO_2_^•−^ **20.10** is a highly nucleophilic radical that can behave as a strong reductant (CO_2_^•−^ E_1/2_ = −2.21 V vs. SCE in DMF) [182,183,184]. Generally, substrates with reduction potentials greater than −2.1 V versus SCE undergo SET reaction pathways, while those with reduction potentials lower than this value undergo radical addition pathways [184].

**Scheme 20:**
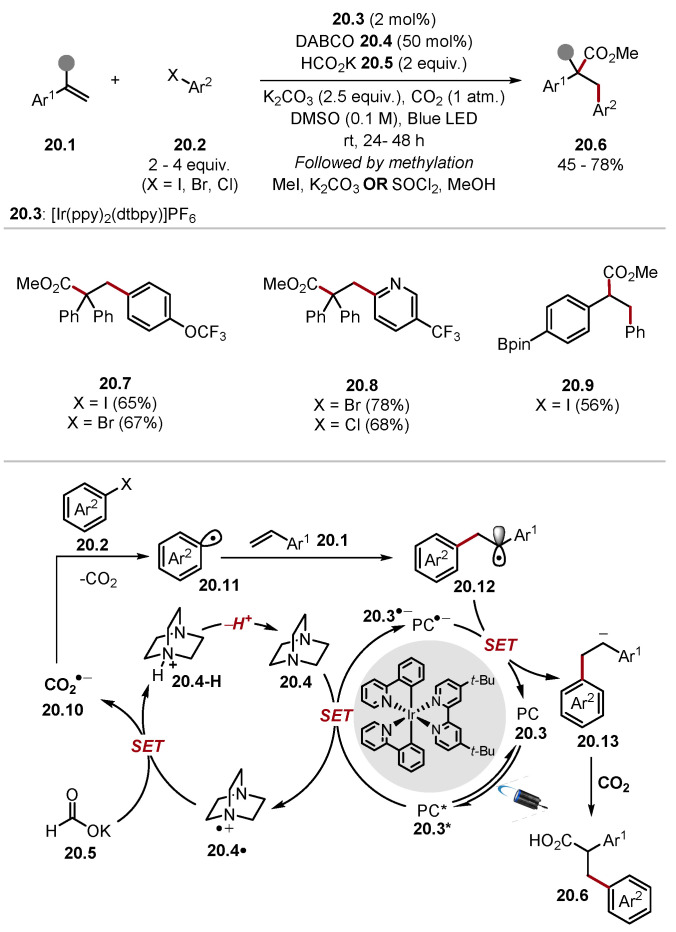
Carboxylative arylation of styrenes **20.1** with CO_2_^•−^.

Li used DABCO and potassium formate to access CO_2_^•−^ **20.10** for an arylative carboxylation of styrenes **20.1** (Scheme 20) [185]. This reaction proceeds through the oxidation of DABCO **20.4** to form DABCO radical cation **20.4·**. DABCO radical cation **20.4·** can abstract a hydrogen atom from formate **20.5** to form CO_2_^•−^ **20.10** [formate HCO_2_^−^ BDE_C–H_ = 86 kcal/mol versus DABCO BDE_N+–H_ = 91.3 kcal/mol] [136,186]. CO_2_^•−^ **20.10** can reduce aryl halides **20.2** to access aryl radical **20.11** ((CO_2_^•−^) E_1/2_ = −2.21 V versus SCE in DMF versus (4-bromobenzotrifluoride) E_1/2_ = −2.18 V vs. SCE in DMF) [187]. The aryl radical **20.11** then adds to styrene **20.1** to form a benzylic radical **20.12**, which is subsequently reduced to the benzylic anion **20.13** by the photocatalyst. The benzylic anion **20.13** traps CO_2_ to form a carboxylate, which is methylated in a subsequent step to form the 1,2-difunctionalised product **20.6**. Electronically diverse aryl bromides and iodides reacted in good yields. However, only electron-poor aryl chlorides were reacted, and no unsuccessful examples were shown.

**Scheme 21:**
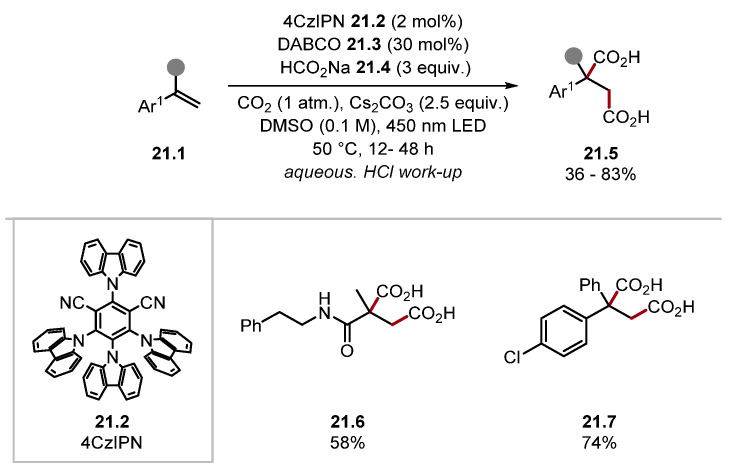
Succinic acid synthesis through dicarboxylation of alkenes with CO_2_^•−^.

Zhu and Guo accessed succinic acid products **21.5** from alkenes **21.1** using a combination of DABCO **21.3** and sodium formate **21.4** under an atmosphere of CO_2_ with photoredox catalysis (Scheme 21) [188].

**Scheme 22:**
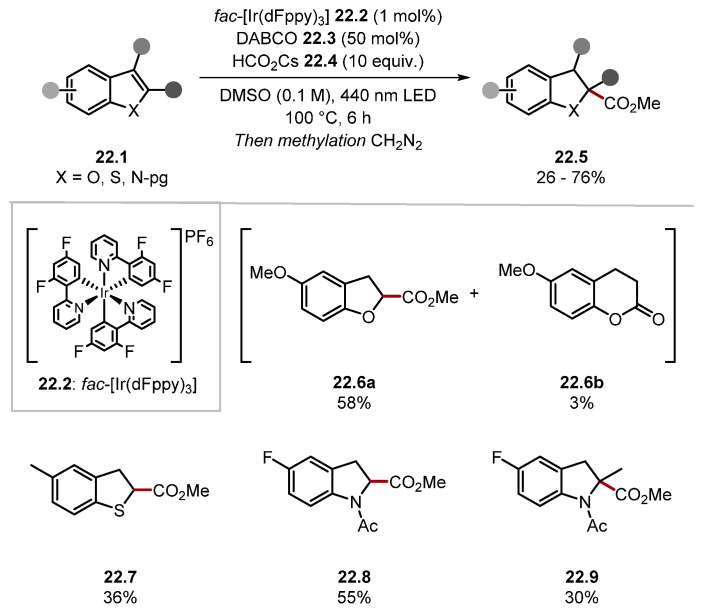
Dearomative carboxylation of heteroarenes **22.1** with CO_2_^•−^.

Mita used a similar combination of DABCO **22.3** and formate salt **22.4** to form CO_2_^•−^ for the addition of heteroaromatics to form products such as benzofurans **22.6**, benzothiophenes **22.7**, and indoles **22.8** and **22.9**) (Scheme 22) [189]. In the case of benzofurans **22.6**, 6-membered lactone by-products were also observed in yields of 3–32%. Various thiols are also used for HAT from formate salts to form CO_2_^•−^ (see Section 2.2.1).

#### 2.1.2. Amide HAT Reagents

Amidyl radicals form amides upon HAT, which have very strong N–H bonds [amide BDE_N–H_ = 97–111 kcal/mol] (Scheme 3) [79]. This makes amidyl radicals powerful HAT abstractors, capable of abstracting unactivated C(sp^3^)–H bonds. In recent years, 1,5-HAT intramolecular protocols using amidyl radicals have been popular, drawing from the classic Hofmann–Löffler–Freytag and Barton reactions [190]. However, the use of amidyl radicals for intermolecular HAT is less explored, and, herein, we cover recent developments in this area [83].

**Scheme 23:**
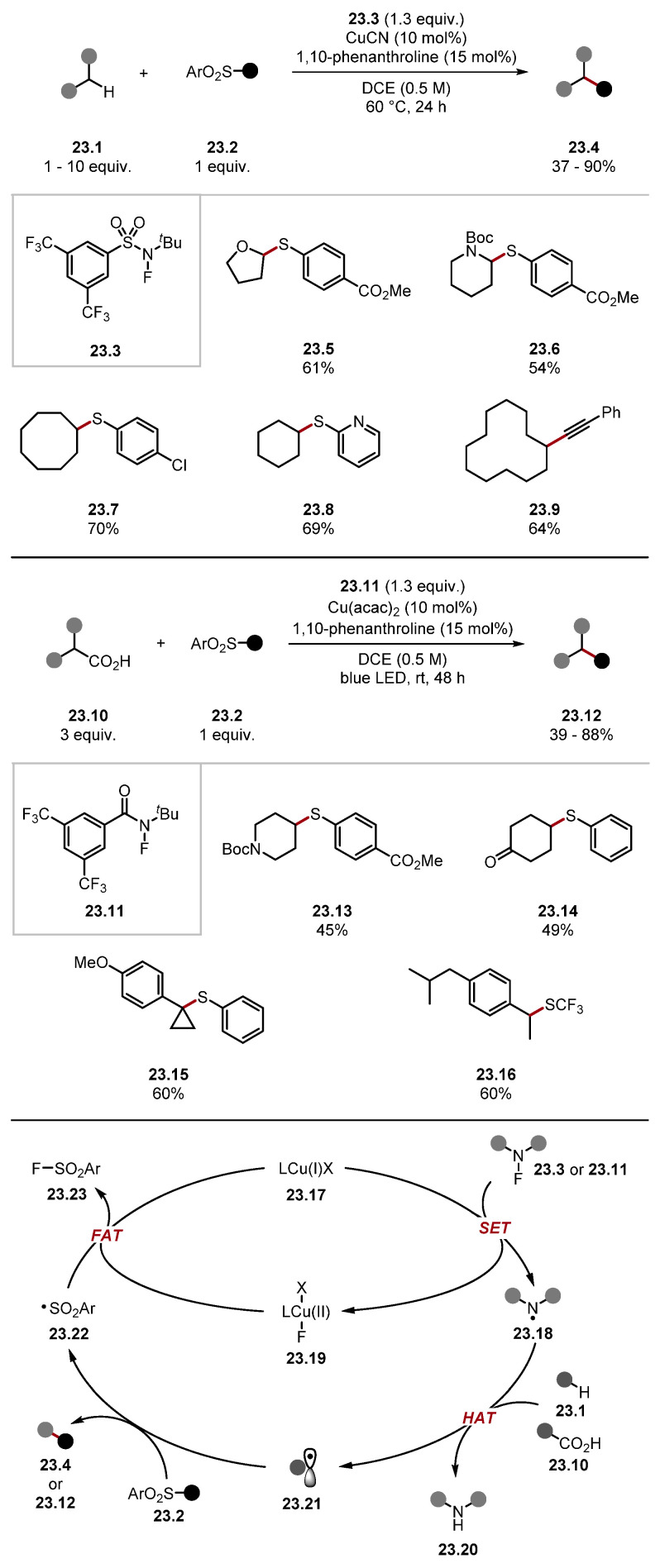
General method for C–H functionalisation using fluoroamides **23.3** and **23.11** as amidyl radical precursors.

In 2021, Hu used amidyl radicals formed by the reduction of *N-*fluorosulfonamide **23.3** or *N-*fluoroamide **23.11** for the functionalisation of strong C(sp^3^)–H bonds and carboxylic acids through decarboxylation (Scheme 23) [191]. Fluorosulfonamide **23.3** and fluorocarboxamide **23.11** were used as radical precursors to access amidyl radicals **23.18**. Numerous C–H substrates were used in the general protocol. For example, THF formed product **23.5**, Boc-protected piperidine formed product **23.6**, and cycloalkanes were also derivatised to form products **23.7**–**23.9**. The authors also developed a decarboxylation protocol from carboxylic acids **23.10**, which, upon decarboxylation and trapping of the radical, yields products **23.12**. This protocol required blue LEDs, and the authors suggest that this could trigger a homolysis of the N–F bonds, as well as forming Cu(I) species from Cu(II) under light irradiation [192,193]. Hence, the authors suggest a HAT step between the O–H of the carboxylic acid and the amidyl radical derived from **23.11**. Using the general protocol, various decarboxylated products were obtained; for instance, Boc-piperidine **23.13**, ketone **23.14**, benzylic thioether **23.15**, and ibuprofen derivative **23.16**. Moreover, using different aryl sulfone radical traps allowed various functional groups to be introduced, including a range of thioethers as well as alkene, alkyne **23.9**, nitriles, trifluoromethylthioether **23.16**, azide, and halogens. The reaction occurs through a reduction of fluoroamides **23.3** or **23.11** with copper (I) **23.17**, forming a copper (II) fluoride salt **23.19** and amidyl radical **23.18** [194,195]. Amidyl radical **23.18** then abstracts a hydrogen atom from the C–H substrate **23.1** or oxidises carboxylic acid **23.10** to form an alkyl radical **23.21**. Alkyl radical **23.21** can be trapped with a multitude of SOMO-philes (radical traps) to form functionalised Product **23.4** or **23.12** and sulfonyl radical **23.22**. Sulfonyl radical **23.22** and copper (II) fluoride **23.19** react forming a sulfonyl fluoride **23.23** and reforming a copper (I) catalyst **23.17** [196]. In contrast to previously reported protocols [197,198,199,200,201], using fluoroamides **23.3** and **23.11** allowed for a general C(sp^3^)–H functionalisation method capable of introducing various functional groups.

**Scheme 24:**
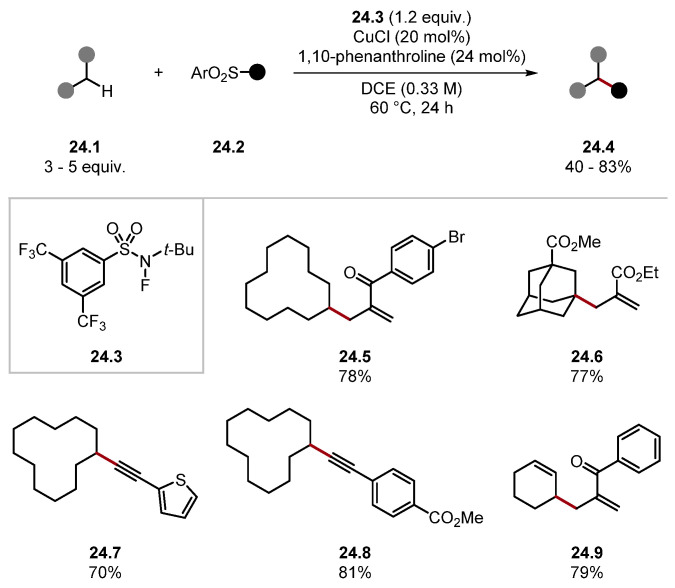
General method for C–H functionalisation using fluoroamide **24.3** as amidyl radical precursor.

Niu used fluorosulfonamide **24.3** for allylation and alkynylation of unactivated C(sp^3^)–H bonds (Scheme 24) [202]. The reaction occurs through a pathway akin to Hu’s protocol (Scheme 23). This method showed good functional group tolerance and incorporated a range of functional groups through numerous SOMO-philes **24.2**. For instance, allylic sulfones formed products **24.5**, **24.6**, and **24.9**, and alkynyl sulfones formed products **24.7** and **24.8**.

**Scheme 25:**
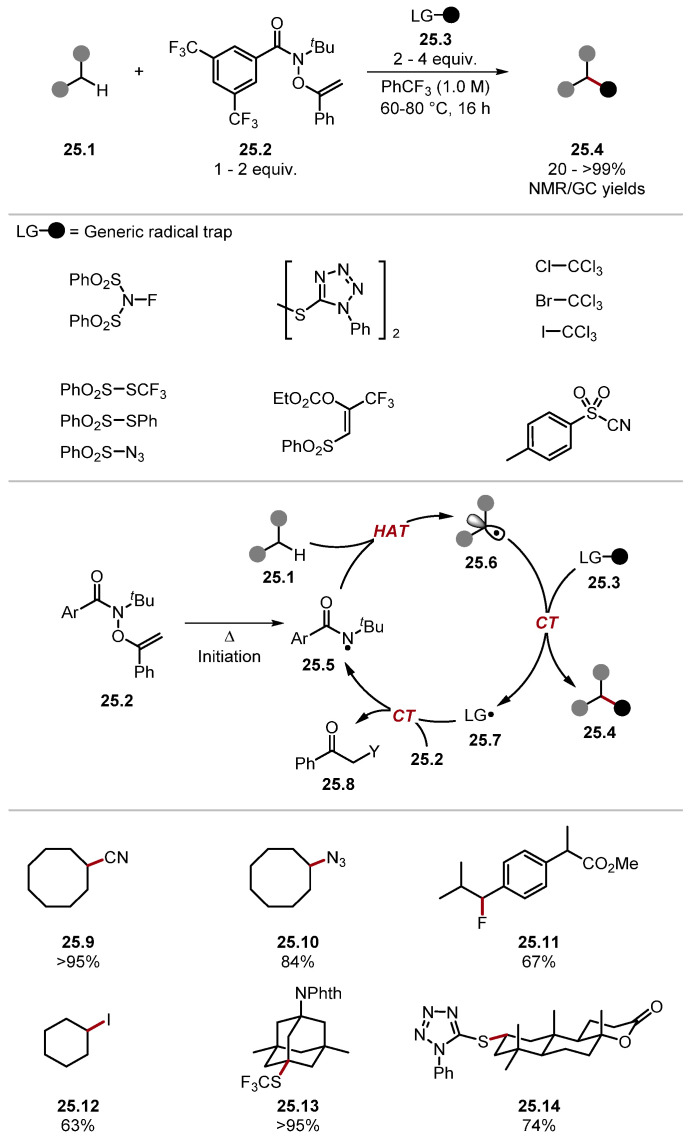
General C–H functionalisation method using amidyl radical precursor **25.2**.

In 2022, Alexanian and Leibfarth developed a general method of aliphatic C(sp^3^)–H functionalisation through HAT using Amide **25.2** (Scheme 25) [203]. This method was an adaptation of Alexanian’s and Leibfarth’s previous works, which used similar amides [197,198,200,204]. However, this work used a HAT reagent precursor **25.2**, allowing different radical traps **25.3** to be used. This allowed various functional groups to be introduced into C–H bonds. Moreover, the C–H substrate was the limiting reagent. The reaction is believed to proceed through a chain propagation mechanism where amide **25.2** undergoes initiation through homolysis or chain transfer (CT) with radical **25.7** forming amidyl radical **25.5**. Amidyl radical **25.5** can abstract hydrogen from unactivatived aliphatic C–H bonds. The alkyl radical **25.6** reacts with a radical trap **25.3** to form C–H functionalised product **25.4** and radical **25.7**. The authors elegantly matched the inherent electrophilicity of the expelled radical **25.7** to trap it with the electron-rich alkene on HAT reagent **25.2** for chain propagation. Cleavage of the resulting radical adduct expels a ketone **25.8** and amidyl radical **25.5**, thus propagating the chain. Various aliphatic compounds were functionalised, and, more importantly, various functional groups were introduced into C(sp^3^)–H bonds. All radical traps outlined were used with cyclooctane forming products in yields of 44–100% NMR/GC yields; for instance, nitrile **25.9** and azide **25.10**. Ibuprofen methyl ester was fluorinated on the benzylic position to form product **25.11**. The iodination product **25.12** was also accessed. Thiolated products **25.13** and **25.14** were also obtained in good yields. Further derivatisations of products were demonstrated, and the protocol was used to introduce functionality into waste-stream aliphatic polymers. This general protocol has enormous potential as various other radical traps may also be used in this manner to introduce an even greater array of functionality into C(sp^3^)–H bonds [76].

**Scheme 26:**
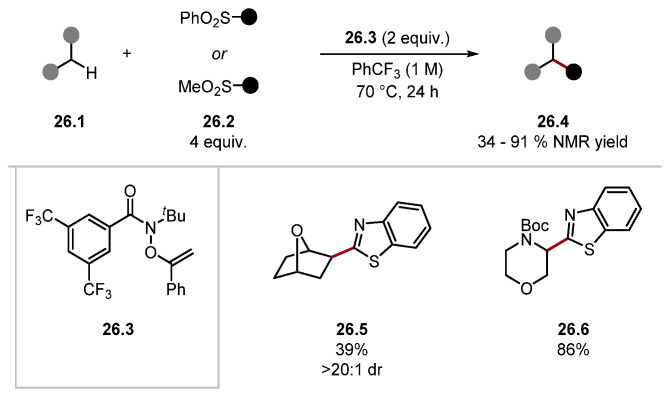
Heteroarylation of C(sp^3^)–H bonds via HAT using amidyl radical precursor **26.3**.

**Scheme 27:**
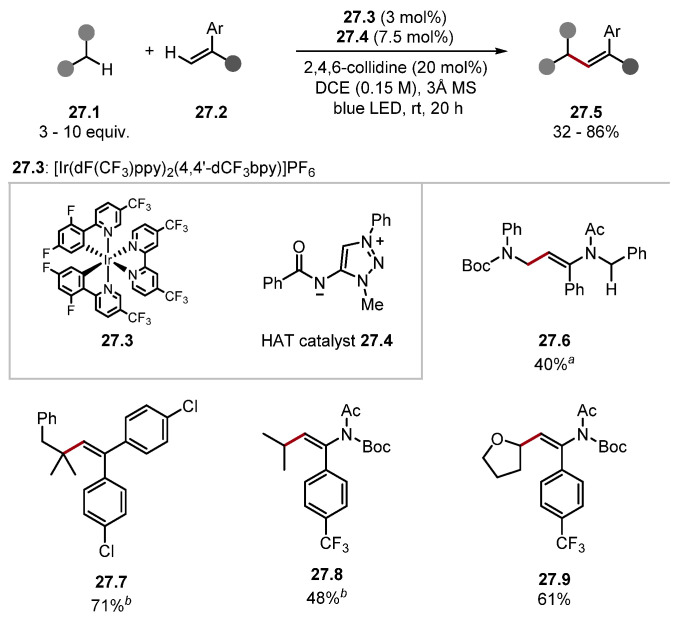
HAT-mediated dehydrogenative cross-coupling using amidyl radical precursor **27.4**. ^a^No 3A MS, ^b^Decarbonylated product made from the corresponding aldehyde.

Alexanian recently built on this work again using HAT reagent **26.3** for a C–H heteroarylation protocol (Scheme 26) [205]. The method used aryl sulfones **26.2** as radical traps, which imposed regioselectivity, negating a common drawback in Minisci-type reactions [127]. Various C–H substrates were derivatised, and the method was selective for the most hydridic C–H bonds, as seen in product **26.6**. Notably, several complex scaffolds were derived regioselectively and diastereoselectively, showcasing the method’s potential for LSF applications. Reagent **26.3** was also recently applied to the functionalisation of B–H bonds in icosahedral carboranes [206]. Ooi has developed a zwitterionic 1,2,3-triazolium amidate HAT catalyst **27.4** [207]. These HAT reagents work on a similar basis to Knowles’s and Alexanian’s works describing amidyl radicals as HAT reagents [83,199,203,204,208]. Amide HAT catalysts, such as **27.4**, form very strong N–H bonds [amide **27.4–H** BDE_N–H_ = 100 kcal/mol], making **27.4** a HAT reagent capable of oxidising strong C–H bonds similar to quinuclidine. Ooi’s previous work shows HAT catalyst **27.4** readily abstracts hydrogen atoms adjacent to carbamates, ethers, aldehydes, and alcohols [207]. In 2022, Ooi used the amidate HAT pre-catalyst **27.4** for a dehydrogenative cross-coupling of various C–H substrates and enamides or 1,1-diarylethenes under irradiation by blue LEDs (Scheme 27) [209].

**Scheme 28:**
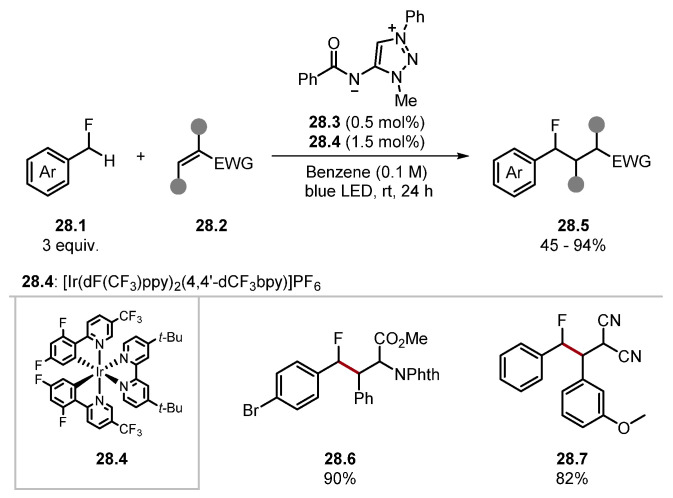
HAT-mediated C(sp^3^)–H alkylation of benzylic fluorides **28.1** with amidyl radical precursor **28.3**.

Carbamates and ethereal α-C(sp^3^)–H were readily alkenylated, forming products **27.6** and **27.9**, respectively. Aldehydes resulted in decarbonylated products such as **27.7** and **27.8**. Various enamide substrates were used in the protocol. Notably, a benzyl protecting group **27.6** was tolerated. Various functionalities were incorporated into arene fragments, such as chloride **27.7** and trifluoromethyl groups **27.8** and **27.9**. Ooi has used HAT pre-catalyst **28.3** for a C(sp^3^)–H alkylation of benzylic fluorides **28.1** (Scheme 28) [210]. The method showed good functional group tolerance with substrates containing halides, ethers, and esters forming products in good yields. Ooi has also recently developed a diphenylphosphinyl amidate HAT catalyst similar to **28.3**, which was used in a Giese protocol with substituted alkanes and cycloalkanes [211].

#### 2.1.3. Azidyl Radical as a HAT Reagent

The azidyl radical **29.7** has previously been used as an oxidant and HAT reagent [212,213]. In recent years, it has mainly been used in the context of primary amine α-C–H HAT. The azidyl radical **29.7** is usually formed through oxidation of its anion **29.10**, although access through homolytic pathways is known. The azidyl radical is inherently electrophilic and abstracts hydridic hydrogen atoms to form hydrazoic acid [hydrazoic acid **29.8** BDE_N–H_ = 92.7 kcal/mol] [214]. Hydrazoic acid **29.8** is easily deprotonated to regenerate the azide anion **29.10**. Due to the facile oxidation of the azide anion to azidyl radical (*^n^*Bu_4_NN_3_ **29.4** E_1/2_ = +0.87 V vs. SCE in MeCN), the reagent can be made catalytic through the use of photoredox catalysis or electrochemistry for oxidation [153,215,216,217]. While the concentration of hydrazoic acid in such reactions is small, it is worth being mindful of hydrazoic acid’s high toxicity and explosive risk [218].

**Scheme 29:**
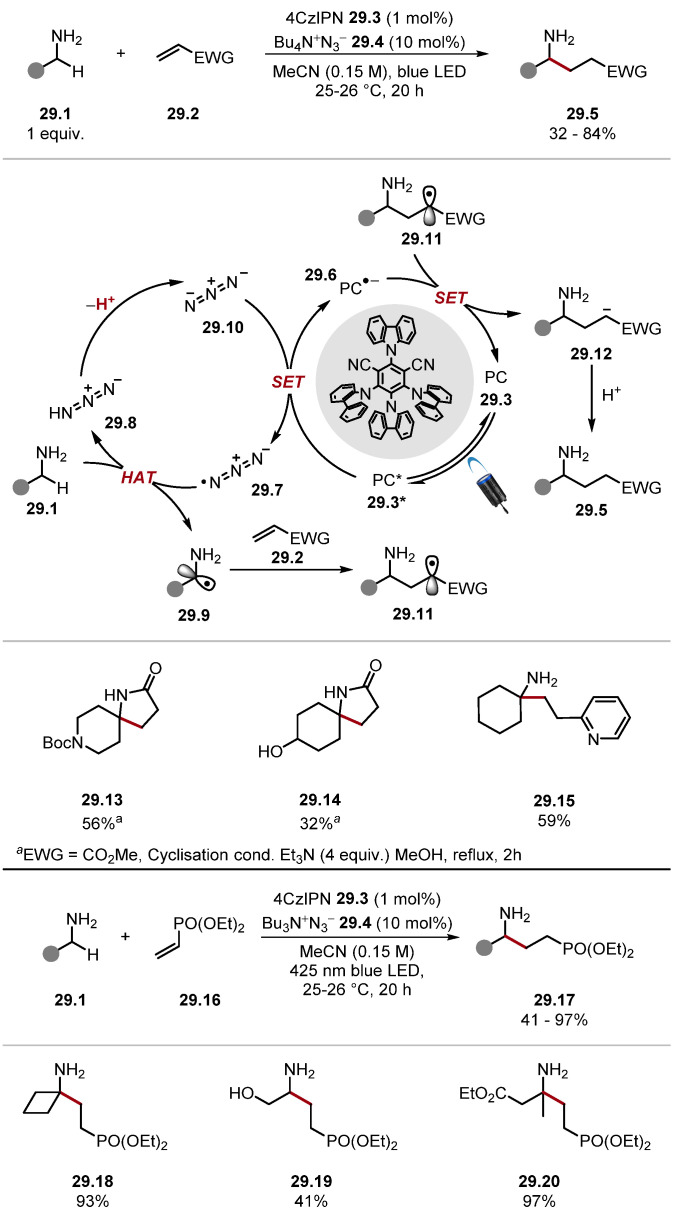
α-C–H alkylation of unprotected primary amines via HAT with azidyl radical.

In 2020, Cresswell used tetrabutylammonium azide **29.4** (which forms azidyl radical **29.7** upon oxidation) for α-C–H alkylation of unprotected amines **29.1** (Scheme 29, top) [215]. When α,β-unsaturated esters were used as Giese acceptors, a separate cyclisation step afforded γ-lactams, such as **29.13**. Previous work sought to develop amine α-C–H alkylation protocols by protecting amines in situ [163]. However, using azidyl radical **29.7** as a HAT catalyst allows unprotected amines **29.1** to be used directly. This reaction proceeds through a HAT and photoredox dual catalysis manifold. The excitation of 4CzIPN **29.3** by blue light produces photoexcited 4CzIPN* 29.3* (E_1/2_ (PC*/PC^•−^) = +1.43 V vs. SCE), which can oxidise azide anion **29.10** (E_red_ = +0.87 V vs. SCE in MeCN) to form 4CzIPN^•−^ **29.6** and azidyl radical **29.7**. Azidyl radical **29.7** abstracts an amino α-C–H from **29.1** to form α–aminoalkyl radical **29.9** [cyclohexylamine BDE_α-C–H_ = 91.1 kcal/mol] [80]. The alkyl radical **29.9** is trapped with a Giese acceptor **29.2** to afford Giese radical adduct **29.11**. The intermediate **29.11** is rapidly reduced by 4CzIPN^•−^ **29.6** (E_1/2_ = (PC/PC^•−^) −1.24 V vs. SCE in MeCN versus E_1/2_ (•CH_2_CO_2_Et/−CH_2_CO_2_Et) = −0.63 V versus SCE in MeCN) and subsequently protonated to form product **29.5**. The low quantum yield (Φ = 0.04) of the reaction suggests that a chain contribution is insignificant. The general protocol showed good chemoselectivity with selectivity for amine α-C(sp^3^)–H bonds, even in the presence of weak carbamate α-C–H bonds **29.13** and benzylic C(sp^3^)–H bonds. Alcohols **29.14**, thioethers, sulfones, and esters (among other functional groups) were tolerated. Numerous Giese acceptors were also used such as 2-vinylpyridine and 4-vinylpyridine. A separate dialkylation protocol was developed for primary amines with two α-C–H bonds. Several derivatisations of primary amines were demonstrated, including reductive amination and amidations. Later that year, Cresswell showed the formation of γ-amino phosphonates **29.17** through the same pathway (Scheme 29, bottom) [216]. The method showed a functional group tolerance akin to the group’s previous reports with cyclobutylamine **29.18**, alcohols **29.19**, esters **29.20**, carbamates, and compounds with benzylic C–H bonds reacting in moderate-to-high yields.

**Scheme 30:**
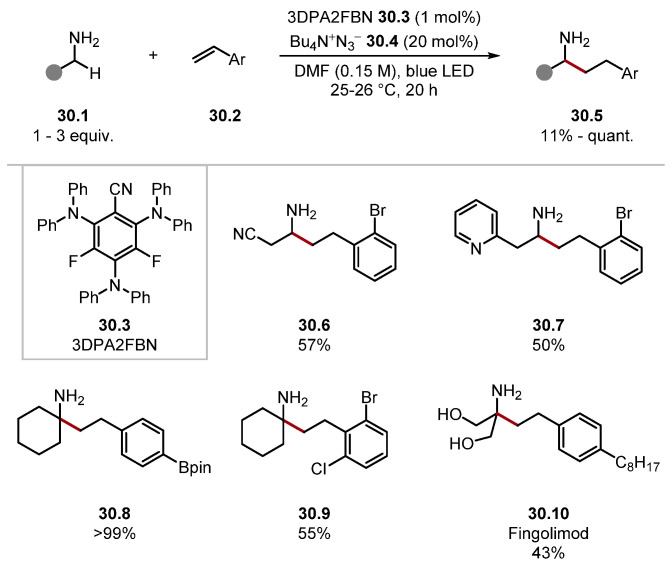
α-C–H alkylation of primary amines **30.1** with styrenes **30.2** through HAT with azidyl radical.

In 2021, Cresswell used tetrabutylammonium azide **30.4** for α-C–H alkylation of unprotected amines **30.1** with styrenes **30.2** (Scheme 30) [153]. The protocol afforded Giese products sluggishly when 4CzIPN was used as a photocatalyst. This was due to the higher reduction potential of benzylic radicals (formed upon radical addition to styrenes, such as **30.2**), compared with radicals with adjacent EWGs (E_1/2_ (**•**CH_2_Ph/−CH_2_Ph) = −1.43 V versus SCE in MeCN compared to E_1/2_ (**•**CH_2_CO_2_Et/−CH_2_CO_2_Et) = −0.63 V versus SCE in MeCN). Hence, when a more strongly reducing photocatalyst 3DPA2FBN **30.3** was used, the product formed in a high yield (E_1/2_ (PC/PC^•−^) = −1.92 V versus SCE in DCM) [135]. The functional group tolerance was excellent on both the amine substrates and the styrene substrates. For instance, silanes, heterocycles **30.7**, nitriles **30.6**, Bpin **30.8**, and halides were tolerated. The general protocol was showcased in a one-step synthesis of Fingolimod **30.10** using a flow set-up. In 2023, Sneha and Orr–Ewing investigated the mechanism of these protocols showing an equivalent of azidyl radical (**•**N_3_), which rapidly reacts with N_3_^−^ to form a cyclic dimer N_6_^•−^, which acts as a reservoir of azidyl radical (**•**N_3_), which carries out the HAT step [219].

**Scheme 31:**
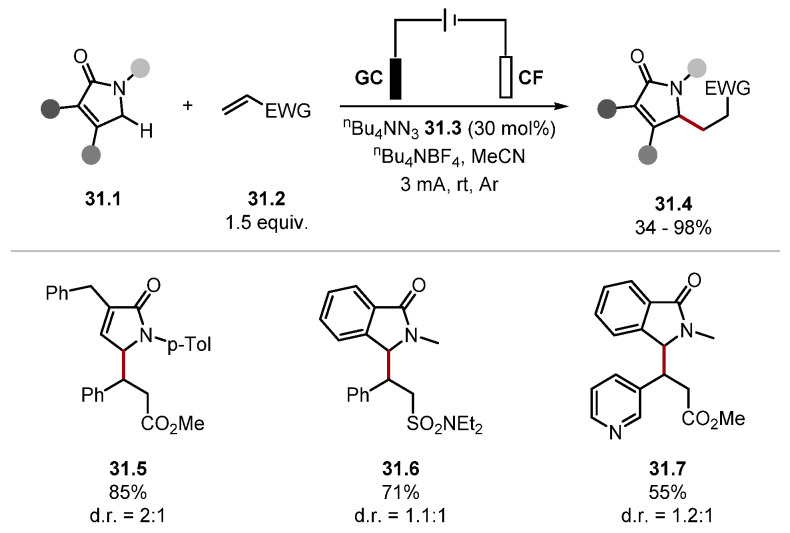
C(sp^3^)–H functionalisation of γ-lactams based on HAT with azidyl radical.

In 2022, Park showed tetrabutylammonium azide **31.3** could be used catalytically under anodic oxidation to generate α-amino radicals for the alkylation of γ-lactams **31.1**, and one δ–lactam example, through HAT with azidyl radical (Scheme 31) [217]. This reaction proceeds through HAT of an α-amino C–H bond with an anodically generated azidyl radical. The radical is subsequently trapped with Giese-acceptors **31.2** to form alkylated products **31.4**. The procedure showed good chemo- and site-selectivity, even in the presence of weaker C–H bonds, as seen in product **31.6**, which contains an allylic C–H bond. Numerous Giese acceptors were used; for instance, α,β-unsaturated sulfonamides **31.6**, α,β-unsaturated sulfones, and α,β-unsaturated phosphonates, among many others. Notably, benzylamines have been problematic substrates in other HAT protocols [153,215].

### 2.2. Sulfur-Based HAT Agents

Thiyl radicals are commonly used in HAT procedures [19,32]. S-centred radicals are less electronegative than their O/N-centred counterparts. However, due to their greater polarisability and the low p*K*_a_ of thiols **7.14** (and thio-acids), thiyl radicals **7.13** are readily accessible through the oxidation of thiolates **7.12** (Scheme 7). Thiyl radicals form S–H bonds (thiols) upon HAT [aliphatic thiols BDE_S–H_ ≈ 87 kcal mol^−1^] [81]. This makes them excellent reagents for HAT from weak (highly activated) C–H bonds, such as α-amino, benzylic, and allylic C–H bonds, as well as weak heteroatom–hydrogen bonds, such as Si–H and Ge–H. Thiols can also be used to close catalytic cycles/reactions through HAT. However, this application is not covered by this review [220,221,222,223,224,225].

#### 2.2.1. Thiols and Thioacid HAT Reagents

**Scheme 32:**
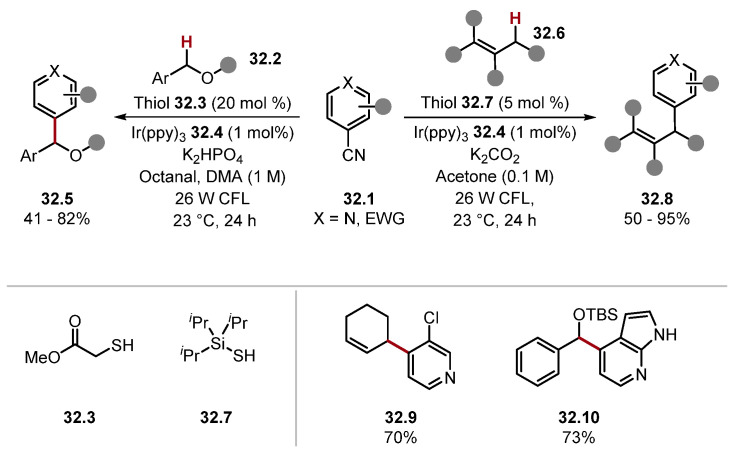
Arylation of benzylic ethers and allylic species with thiyl radicals.

MacMillan and co-workers demonstrated the arylation of benzylic ether **32.5**, and allylic **32.8** C(sp^3^)–H bonds proceeding through a coupling of an alkyl radical and a persistent arene radical anion (Scheme 32) [119,226].

**Scheme 33:**
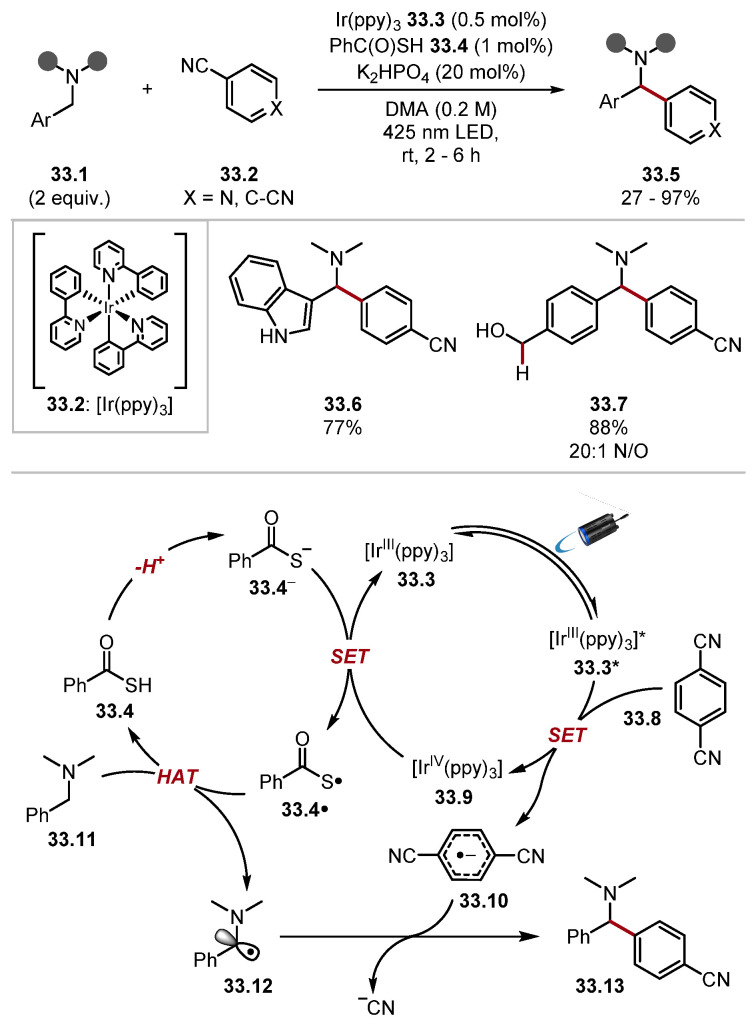
Arylation of benzylamine C(sp^3^)–H though HAT with thiobenzoic acid **33.4**.

These protocols displayed excellent functional group tolerance with respect to all reactants. For instance, alcohols, N/S/O-containing heterocycles **32.9** and **32.10**, and halogen substituents were tolerated. Moreover, in both protocols, only monoarylated products were observed. These works showed that thiyl radicals can abstract hydrogen atoms from allylic and benzylic C(sp^3^)–H bonds, inspiring countless protocols proceeding through similar mechanistic pathways with thiyl radicals. The Hamashima group found that benzylamines **33.1** were arylated through a coupling between radical and radical anion akin to that of the MacMillan group (Scheme 33) [82]. Thiobenzoic acid was used as a HAT reagent precursor as it is readily deprotonated (thioacetic acid, p*K*_a_ = 3.2) [227] and can subsequently be oxidised even in the presence of amines (PhC(O)SK E_1/2_ +0.80 V versus Ag/AgCl in DMA versus *N*,*N-*dimethylbenzylamine **33.11** E_1/2_ = +1.25 V vs. Ag/AgCl in DMA). The method was amenable to late-stage C(sp^3^)–H arylation of several pharmaceuticals and showed outstanding functional group tolerance as heterocycles and primary amines and alcohols (among others) were tolerated (see products **33.6** and **33.7**).

**Scheme 34:**
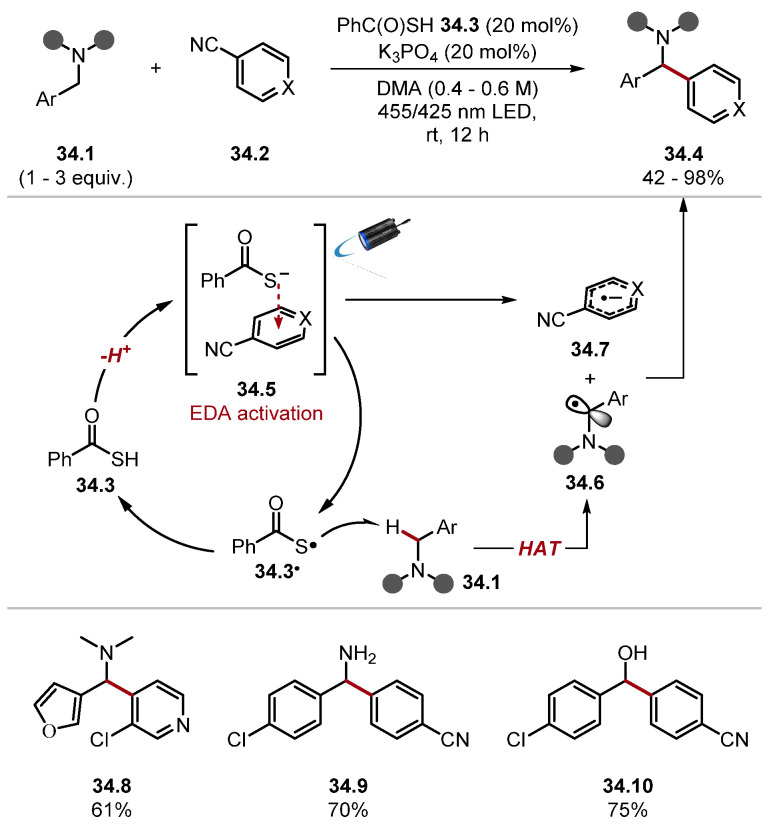
Benzylamine C(sp^3^)–H arylation through EDA-initiated HAT using thiobenzoic acid.

The reaction proceeds though photoexcitation of Ir(ppy)_3_ **33.3** by 425 nm blue light, forming a strong reductant [Ir^III^ (ppy)_3_]* 33.3* (E_1/2_ (Ir^IV^/*Ir^III^) = −1.73 V vs. SCE in MeCN), which reduces Terephthalonitrile **33.8** (E_1/2_ = −1.61 V versus SCE in MeCN) to form aryl radical anion **33.10**. The HAT catalytic cycle proceeds by deprotonation of thiobenzoic acid **33.4** (thioacetic acid, p*K*_a_ = 3.2) [227]. The thiobenzoate anion **33.4^−^** is then oxidised by [Ir^IV^(ppy)_3_] **33.9** (E_1/2_ (Ir^IV^/Ir^III^) = +0.77 V vs. SCE in MeCN) to form the S-centred radical **33.4·**, which can abstract a hydrogen atom from *N-*benzylamine **33.11** [thiobenzoic acid **33.4** BDE_S–H_ = 87.4 kcal/mol versus benzylamine **33.11** BDE_α-C–H_ = 84.9 kcal/mol]. The benzylic radical **33.12** then undergoes a radical-radical anion coupling with **33.10** to form product **33.13** [26,228]. The Hamashima group subsequently developed a photocatalyst-free arylation of benzylamine **34.1** C(sp^3^)–H bonds with thiobenzoic acid **34.3** as a HAT reagent (Scheme 34) [229]. In this work, donor-acceptor complex **34.5** is believed to initiate the formation of S-centred radical **34.3**, as well as arene radical anion **34.10** upon excitation by visible light [230,231,232]. Control experiments showed that the addition of *N*,*N*-dimethylbenzylamine to PhC(O)SK caused an absorption of visible light around 400–450 nm. The authors do not elucidate the EDA complex, which initiates the reaction and a UV–VIS spectrum of PhC(O)SK and terephthalonitrile; another electron acceptor was not investigated. S-centred radical **34.3·** would undergo a HAT process with benzylamine **34.1** to generate an α-aminoalkyl radical **34.6**. The α-aminoalkyl radical **34.6** could then combine with arene radical anion **34.7**, affording the product **34.4**.

**Scheme 35:**
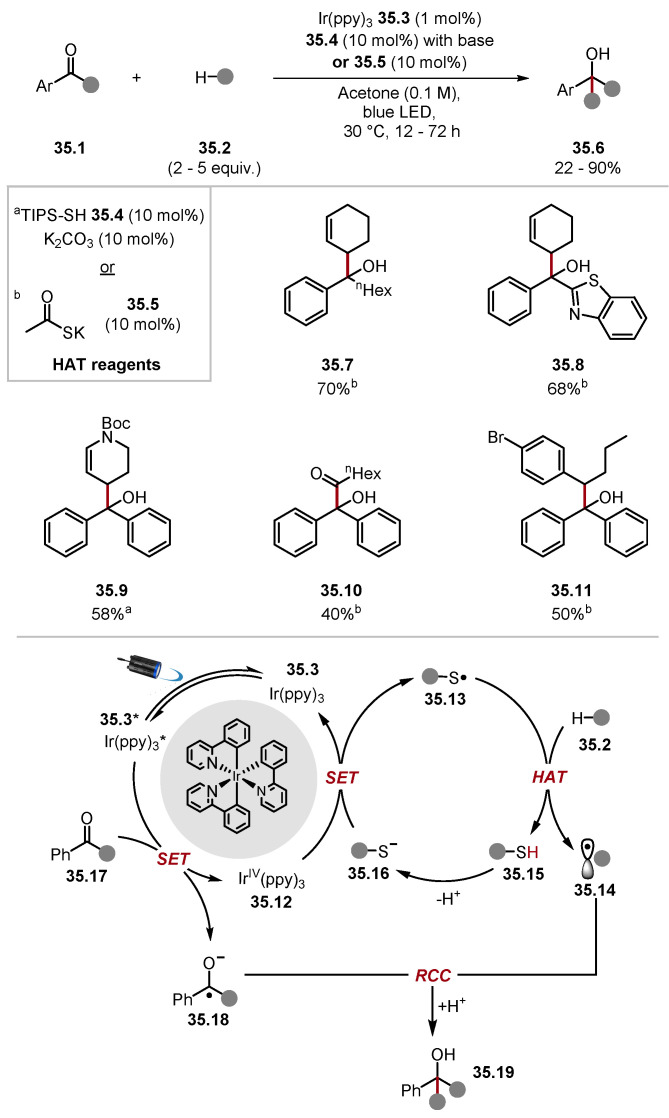
Ketone carbonyl alkylation through radical–radical anion coupling mediated by photoredox catalysis and HAT with triisopropylsilanethiol and thioacetic acid. ^a^ TIPSH **35.4** and K_2_CO_3_ were used. ^b^ Thioacetate salt **35.5** was used.

Liu used thiol HAT catalysts to abstract allylic and benzylic hydrogen atoms to form alkyl radicals for radical–radical anion couplings with ketyl radicals/ketyl radical anions to form tertiary alcohol products (Scheme 35) [233]. Two general protocols were developed; one used TIPS–SH **35.4**, and the other used thioacetate salt **35.5**. Both diaryl ketones and alkyl aryl ketones were suitable substrates. Aliphatic ketones did not react. Numerous heterocycles were tolerated, including benzothiazole, as seen in product **35.8**. The protocol mainly functionalised allylic and benzylic C–H bonds, with one example of aldehyde C–H functionalisation. The mechanism of the reaction is believed to occur through reductive SET to ketone **35.17** by Ir(ppy)_3_ **35.3** to form a ketyl radical anion **35.18** (benzophenone E_1/2_ = −1.66 V vs. Ag/AgCl versus E_1/2_ (Ir^IV^/Ir^III^) = −1.73 V versus SCE) [234,235]. Ir(IV)(ppy)_3_^−^ **35.12** is sufficiently strong to oxidise thiolate **35.16** to thiyl radical **35.13**, which is capable of abstracting weak allylic or benzylic C(sp^3^)–H bonds to form alkyl radical **35.14**. The alkyl radical **35.14** subsequently undergoes radical–radical anion coupling with the ketyl radical anion **35.18** to form tertiary alcohols **35.19**. Thiyl radicals have also been used for the functionalisation of Si–H bonds [151,236].

**Scheme 36:**
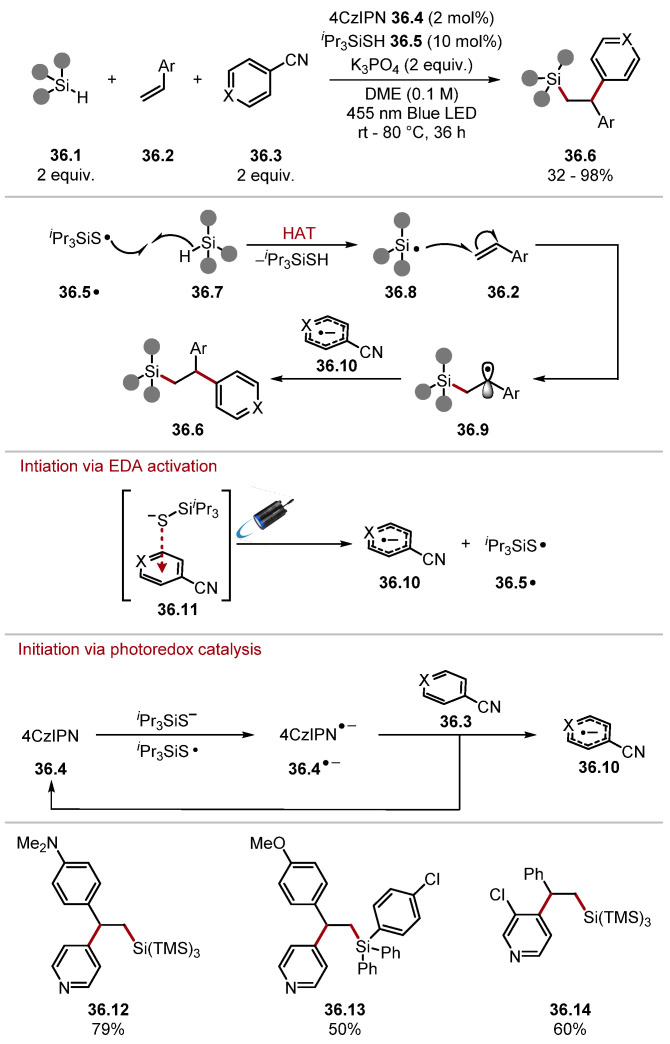
Arylsilylation of styrenes **36.2** with hydrosilanes **36.1** and cyanoarenes **36.3** proceeding through HAT with triisopropylsilanethiol.

In 2022, Lin developed an arylsilylation of styrenes **36.2** with hydrosilanes **36.1** and cyanoarenes **36.3** under irradiation with blue LEDs (Scheme 36) [237]. The reaction proceeds through HAT of a silane **36.1** Si–H with thiyl radical **36.5** to form silyl radical **36.8** [thiols BDE_S–H_ ≈ 87 kcal/mol versus Ph_3_SiH BDE_Si–H_ = 86.4 kcal/mol] [79,81]. The silyl radical **36.8** can add into a styrene **36.2** to form a benzylic radical adduct **36.9**. The benzylic radical adduct **36.9** then undergoes a radical-radical anion cross-coupling, with radical anion **36.10** expelling a cyanide to form the arylsilylated product **36.6**. There are two competing initiation mechanisms in this reaction: In one, the radical anion **36.10** and thiyl radical **36.5•** can form through EDA complex **36.11** [238]. Alternatively, the process is initiated by 4CzIPN **36.4**, although it should be noted 4CzIPN^•−^ (**36.4**^•−^) is not sufficiently strong to reduce 4-cyanopyridine to its radical anion **36.10** (E_1/2_ (PC/PC^•−^) = −1.24 V versus SCE in MeCN versus 4-cyanopyridine E_1/2_ = −1.86 V vs. SCE in MeCN) [135,239]. Moreover, reactions in which 4-cyanopyridine or 1,4-dicyanobenzene are reduced to radical anions feature more strongly reducing photoredox catalysts [119,226]. Additionally, control experiments showed that the reaction occurred in 64% yield in the absence of photocatalyst, compared with 92% for optimal conditions, suggesting initiation through an EDA complex is the major pathway. Hence, the mechanistic pathway of this protocol is not fully understood. The protocol showed good functional group tolerance towards all three reactants with products featuring amines **36.12**, amides, ethers **36.13**, and halides **36.14**, among other functionalities being prepared in fair-to-excellent yields. Silanes containing alkyl substituents provide the lowest yields.

**Scheme 37:**
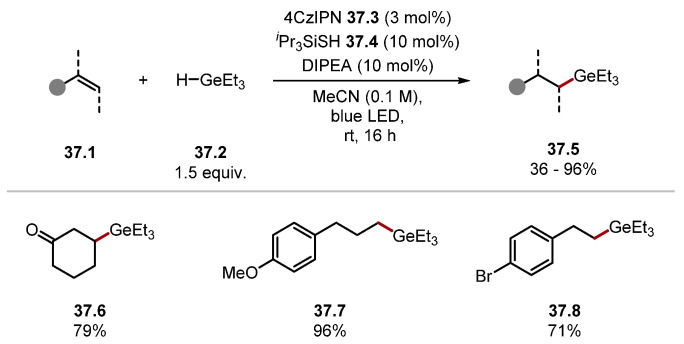
Hydrogermylation of olefins via HAT of H–GeEt_3_ **37.2** with triisopropylsilanethiol.

In 2021, Schoenebeck used *^i^*Pr_3_SiSH **37.4** to abstract hydrogen from Ge–H bonds to facilitate a hydrogermylation of alkenes (Scheme 37) [240]. Notably, organogermanes have shown enormous potential as functional handles [241]. Various olefins were tolerated with the procedure being relatively insensitive to the electronic nature of the olefin. The good yield of product **37.8** from 4-bromostyrene and product **37.7** from an unactivated alkene may indicate an innate chain propagation as benzylic radicals are reduced slowly by 4CzIPN^•−^ [153]. However, no quantum yields were measured. DFT studies showed an abstraction of hydrogen from Et_3_Ge–H, while TIPS–S**·** was thermodynamically favourable [Et_3_Ge–H BDE_Ge–H_ = 86.0 kcal/mol versus alkyl thiol BDE_S–H_ ≈ 87 kcal mol^−1^] [79,81].

**Scheme 38:**
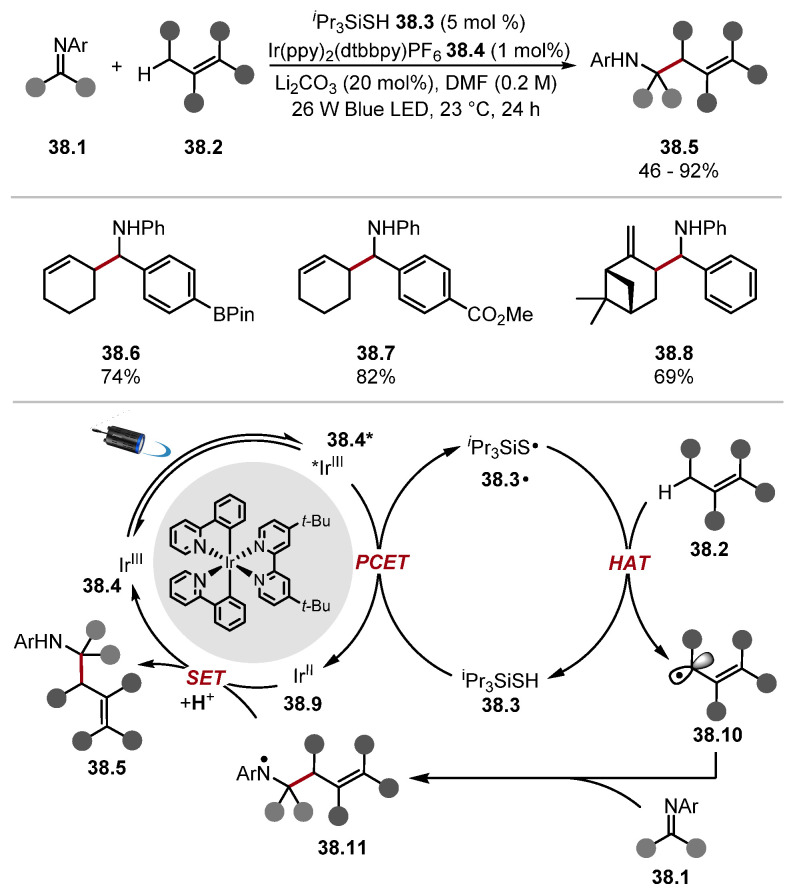
Allylic C(sp^3^)–H alkylation through HAT with triisopropylthiol and photoredox catalysis.

**Scheme 39:**
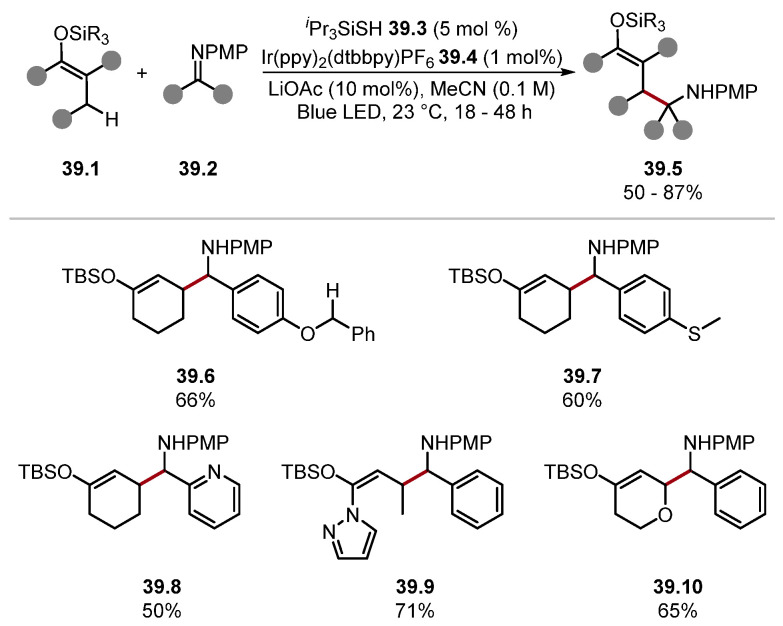
Silyl enol ether β-C(sp^3^)–H Mannich-type alkylation through HAT with triisopropylthiol and photoredox catalysis.

Huang and Rueping developed an allylic C(sp^3^)–H alkylation protocol proceeding through photoredox HAT dual catalysis using triisopropylsilanethiol **38.3** as HAT reagent (Scheme 38) [242]. The assessment of the substrate scope for this general protocol showed a good functional group tolerance with heterocycles (thiophene and pyridine) and pinacolborane, as seen in products **38.6**, esters **38.7**, as well as halogens and ethers tolerated on the imine substrate. The general conditions were also used to functionalise natural products, as seen in the formation of compound **38.8**. The Ooi group developed a similar protocol for silyl enol ethers **39.1**, where a β-C(sp^3^)–H Mannich-type alkylation of imines **39.2** is accomplished using TIPS–SH **39.3** (Scheme 39) [243]. This protocol also displayed a wide functional group tolerance with ethers, thioethers **39.7**, and various heterocycles being tolerated. The reactions also proceeded well in the presence of a weak benzyl ether C(sp^3^)–H bond, as seen in the formation of **39.6**. The quantum yield of this reaction (average Φ = 0.092) shows that chain contribution to the reaction is not significant, adding evidence to the mechanism previously suggested by Huang and co-workers [242].

**Scheme 40:**
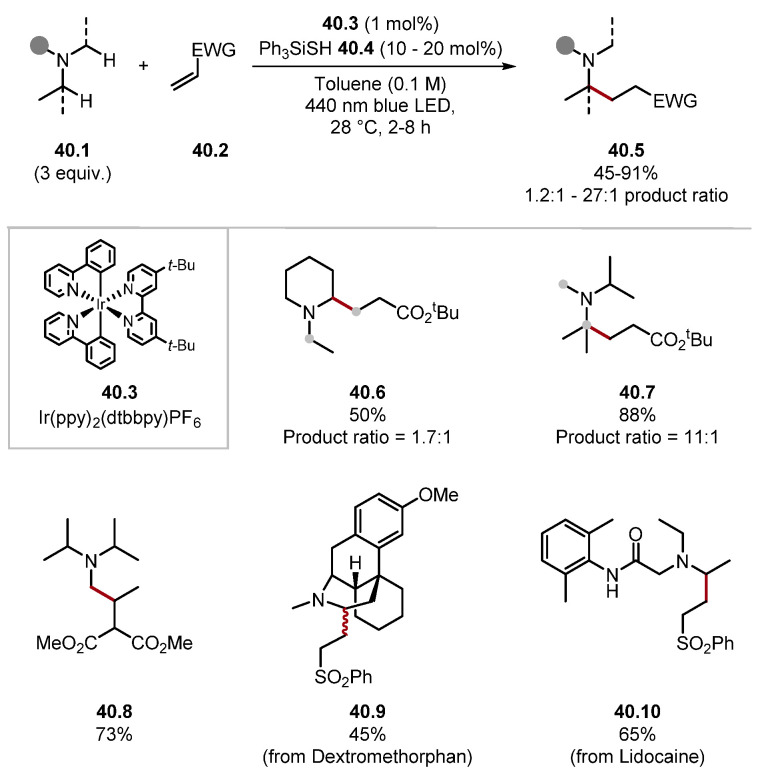
Site selective α-C–H alkylation of trialkylamines through a reversible HAT step with triphenylsilanethiol.

In 2021, Rovis and Schoenebeck developed a site-selective α-C–H alkylation of trialkylamines **40.1** through a Giese addition by establishing a reversible HAT step with triphenylsilanethiol **40.4** (Scheme 40) [133]. Establishing an equilibrium in the HAT step allowed for both the less and more substituted radicals to form as their BDE vales are almost identical. However, the more nucleophilic (more substituted) α-amino radical undergoes Giese addition faster [85]. Hence, the selectivity of this protocol is guided by the Curtin–Hammett principle. ^13^C NMR was used to predict the regioselectivity of the alkylation as α-amino C atoms, with more downfield shifts reacted more favourably. The evaluation of the substrate scope for the protocol showed numerous functional groups were tolerated, such as alcohols, ketones, and amides (see product **40.10**), among many others. Site-selectivity for the more substituted alkyl group ranged from 1.3:1 to 27:1 (where ratio is mentioned), and there were notable exceptions in attainable site selectivity. *N*-Ethylpiperidine was alkylated on the ring, as seen in product **40.6**. Additionally, where the Giese acceptor had substituents on the terminal carbon site-selectivity had reversed, as seen by product **40.8** [85]. The method was also showcased on 12 pharmaceuticals, such as Dextromethorphan **40.9** and Lidocaine **40.10**, demonstrating potential for late-stage functionalisation. It is worth noting the contrast in selectivity compared with similar C–H functionalisation methods [244,245].

**Scheme 41:**
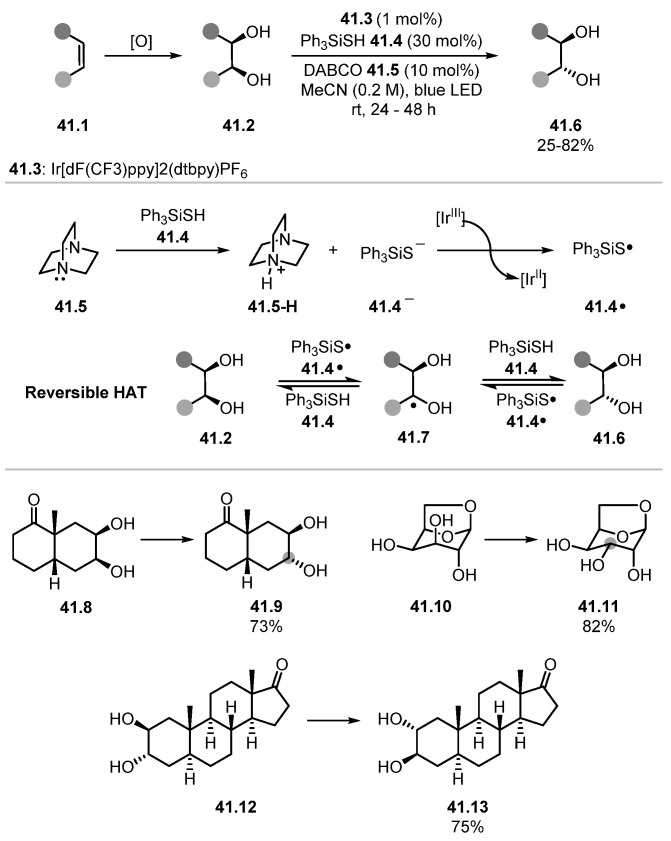
Reversible HAT for *tran*s-selective stereochemical editing of vicinal diols with triphenylsilanethiol.

Wendlandt used Ph_3_SSiH **41.4** to reversibly abstract alcohol α-C(sp^3^)–H bonds to establish an equilibrium, which leads to stereochemical editing of vicinal diols through thermodynamic control (Scheme 41) [246].

This protocol changes stereochemistry of vicinal diols from *cis*-diols **41.2** to *trans*-diols **41.6**. DABCO **41.5** serves as a base, deprotonating Ph_3_SiSH **41.4** to a thiolate **41.4^−^**, which is oxidised to a thiyl radical **41.4·** by the photoredox catalyst **41.3**. The thiyl radical **41.4·** can reversibly abstract the alcohol α-C(sp^3^)–H atom where this equilibrium will favour the *trans*-isomer **41.6**. Thiyl radicals have been used in previous efforts to edit stereochemistry through reversible HAT steps [247]. Various diols were edited using this method affording the products in low-to-excellent yields. Despite lower yields for certain substrates, the authors note that traditional means of changing stereochemistry of diols can take numerous steps. The method was also highly chemoselective, with substrates containing sensitive functional groups like ketones and acetals reacting in high yields, as seen in products **41.9** and **41.11**. Complex structures like *trans*-diaxial diastereomer **41.12** were converted at both α-hydroxy C–H bonds to form product **41.13**.

**Scheme 42:**
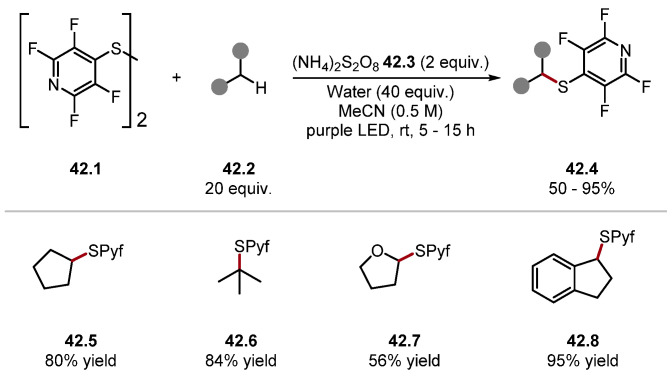
Thiolation of unactivated C(sp^3^)–H bonds using (SPyf)_2_ **42.1**.

In 2021, the Dilman group demonstrated an example of a thiyl radical activating unactivated alkanes’ C(sp^3^)–H bonds for thiolation (Scheme 42) [248]. The disulfide **42.1** undergoes a homolysis of the S–S bond to generate two thiyl radicals capable of HAT. Screening experiments showed diphenyl disulfide provided no product, while ((C_6_F_5_)S)_2_ provided less than a 5% yield of the product. ((Pyf)S)_2_ **42.1** was able to thiolate even unactivated C(sp^3^)–H bonds of alkanes to form products such as **42.5 and 42.6**. Various saturated heterocycles formed products in good yields (such as product **42.7**), and benzylic C(sp^3^)–H bonds were also amenable (**42.8**).

**Scheme 43:**
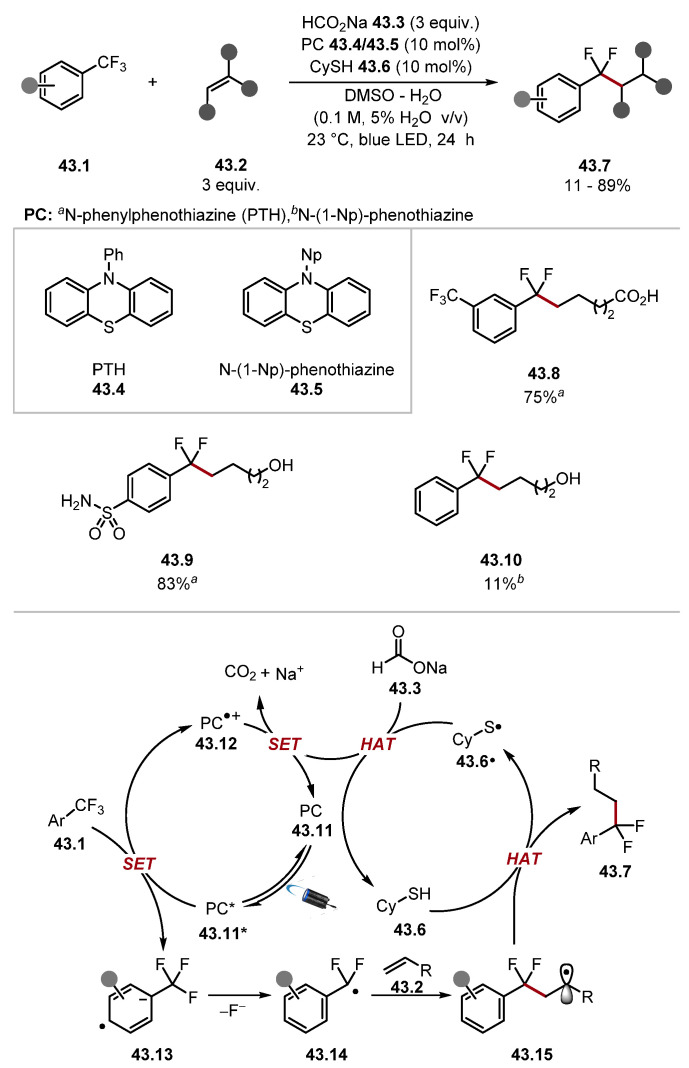
Defluorinative alkylation of trifluorotoluenes **43.1**.

**Scheme 44:**
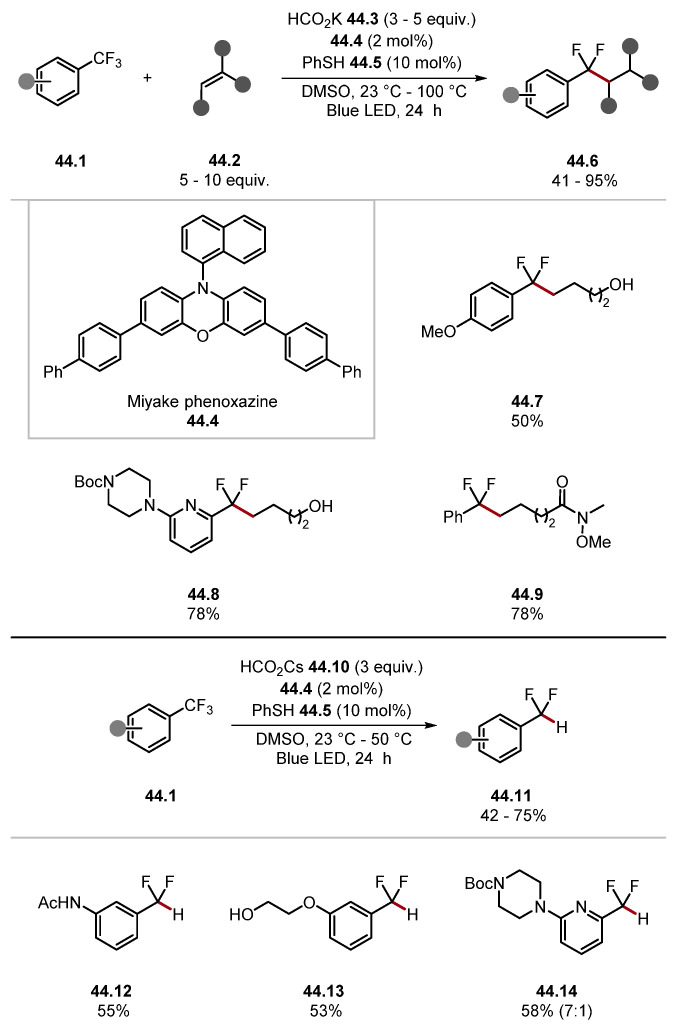
Defluorinative alkylation and hydrodefluorination of trifluorotoluenes **44.1**.

**Scheme 45:**
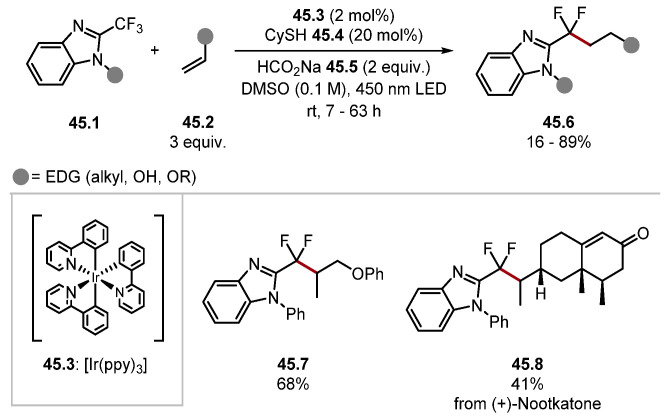
Defluorinative alkylation of trifluoromethylbenzimidazoles.

Thiols have also been used as HAT reagents in the context of CO_2_^•−^ formation via HAT from formate salts [181]. In 2018, Jui developed a defluorinative alkylation of trifluorotoluenes **43.1** (Scheme 43) [249]. Mechanistically, this reaction worked through reductive SET of trifluorotoluenes **43.1** with photoexcited *N-*phenylphenothiazine (PTH*) 43.4* to form radical anion **43.13**. The radical anion **43.13** would then expel a fluoride to form a difluorobenzylic radical **43.14**, which is trapped by an olefin to afford radical **43.15**. Cyclohexanethiol **43.6** would quench radical **43.15** to form the product **43.7**. The resulting thiyl radical **43.6·** abstracts a hydrogen atom from the formate **43.3** to form CO_2_^•−^. CO_2_^•−^ completes the catalytic cycle by restoring photocatalyst **43.11** and releasing CO_2_. This paper was a landmark for both CO_2_^•−^ and trifluorotoluene defluorinative reactions. However, the scope of trifluorotoluenes **43.1** was limited to activated trifluorotoluenes with additional EWGs. In 2019, Jui built on his previous work by developing a similar protocol, which tolerated substrates containing electron-donating groups (Scheme 44) [250]. Further optimisation found Miyake’s phenoxazine **44.4** as a photocatalyst, thiophenol **44.5** as an HAT catalyst, and an elevated temperature of 100 °C to be optimal. General defluorinative alkylation and hydrodefluorination protocols were developed. While BDE values between formate C–H and thiophenol [thiophenol BDE_S–H_ = 83.3 kcal/mol versus formate HCO_2_^−^ BDE_C–H_ = 86 kcal/mol] are not matching, the elevated temperature and potential for competing initiation through formate oxidation in DMSO mean that CO_2_^•−^ can form through several pathways [251,252]. Subsequent work by Jui found a similar protocol has a quantum yield (Φ) of 2.63, indicating a radical chain contribution [184]. Additionally, in similar studies by Wickens, Stern–Volmer studies showed methyl thiosalicylate-quenched excited state 4DPAIPN at a faster rate than a formate salt [252]. In 2022, Zhu, Guo, and Zhu described a similar protocol for defluorinative alkylation of trifluoromethylbenzimidazoles **45.1** (Scheme 45) [253].

Thiols have also been used to promote CO_2_^•−^ formation via HAT from formate salts in elegant protocols by Molander, Glorius, and Wickens [183,252,254,255,256,257,258,259]. However, due to mechanistic complexities associated with initiation and the similarity with the HAT chemistry already mentioned, these works have been omitted.

#### 2.2.2. BINOL-Derived Thiophosphoric Acids

In recent years, thiyl radicals formed from BINOL-derived thiophosphoric acids have been used for HAT processes. Such reagents have been shown to effectively abstract hydrogen atoms from C–H bonds up to 96 kcal/mol [260]. This has allowed them to be used in a wider range of HAT processes than traditional thiyl radicals covered in Section 2.2.1.

**Scheme 46:**
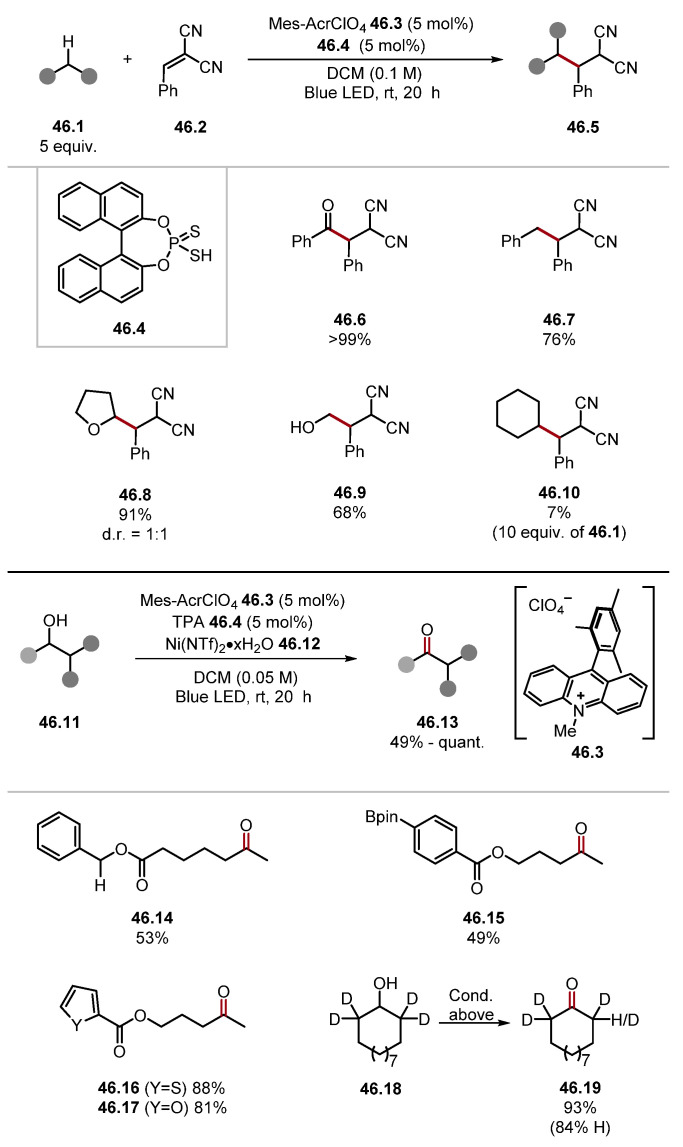
Thiophosphoric acid **46.4** as a HAT catalyst and CAD of secondary alcohols.

**Scheme 47:**
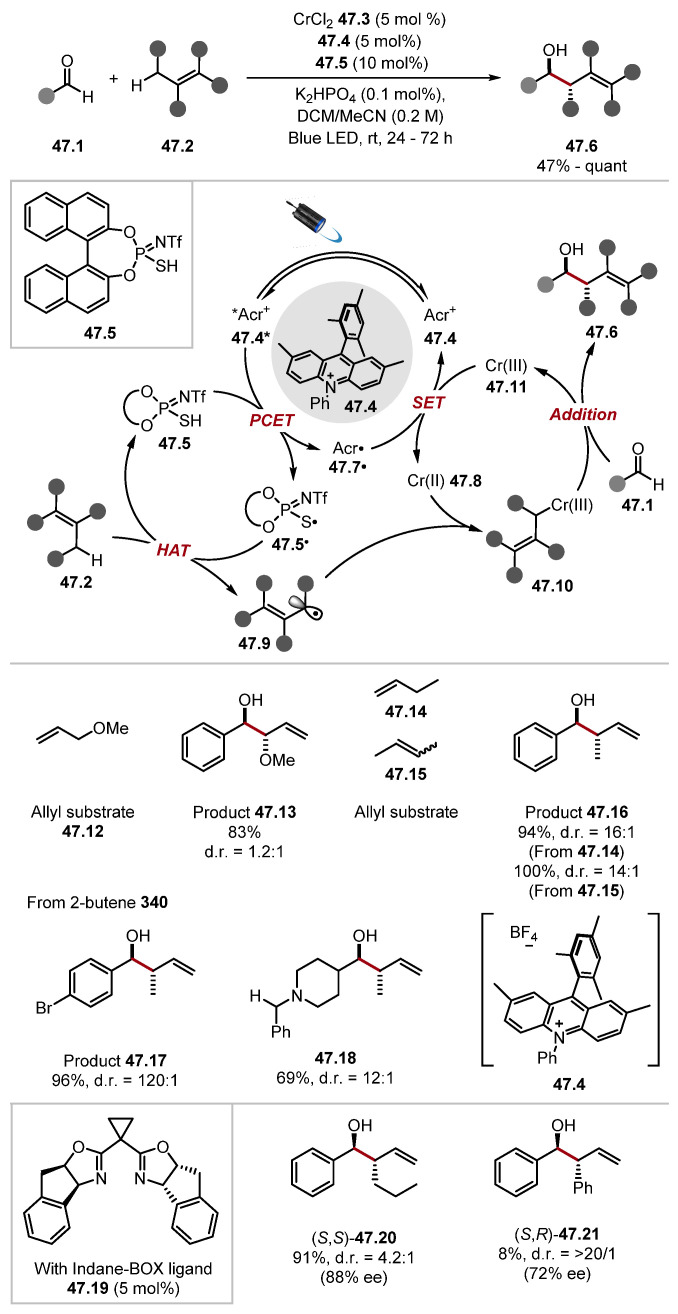
Allylation of aldehydes through ternary catalysis with thiophosphoric imide **47.5**.

In 2020, the Kanai group reported the use of thiophosphoric acid **46.4** as a HAT reagent in a catalytic acceptorless dehydrogenation (CAD) of secondary alcohols **46.11** to ketones **46.13** (Scheme 46) [260]. Initial studies investigated thiophosphoric acid **46.4** in combination with an acridinium photocatalyst **46.3** for a HAT-photoredox tandem catalysis system in a Giese pathway using benzylidene malononitrile **46.2**. This system successfully alkylated various hydridic C–H bonds. Namely, formyl C(sp^2^)–H bonds (see product **46.6**), benzylic C(sp^3^)–H bonds (**46.7**), and ethereal C(sp^3^)–H bonds (**46.8**) were alkylated effectively [benzaldehyde BDE_C–H_ = 88.7 kcal/mol and toluene BDE_C–H_ = 89.3 kcal/mol and THF BDE_C–H_ = 92.1 kcal/mol] [79]. Alkylation of α-alcohol C(sp^3^)–H was less effective (**46.9**) [methanol BDE_α-C–H_ = 96.2 kcal/mol] and strong aliphatic C(sp^3^)–H bonds of cyclohexane **46.10** were abstracted slowly [cyclohexane BDE_C–H_ = 99.5 kcal/mol]. Overall, this study showed thiophosphoric acid **46.4** and related binol-derived thiophosphoric acid HAT reagents are capable of abstracting stronger C–H bonds than standard thiol HAT catalysts (Section 2.2.1). Kanai also demonstrated a ternary catalytic system for the CAD of secondary alcohol **46.11**, combining HAT, photoredox, and nickel catalysis. The general protocol provided moderate-to-quantitative yields and possessed a good chemoselectivity profile. The mechanism of the reaction is believed to proceed through HAT of the alcohol α-C–H bond to form an alkyl radical that is captured by the nickel catalyst, which undergoes a reductive SET and subsequent β-hydride elimination to form an enol (ketone). The β-hydride elimination step was probed through the scrambling of deuterium in product **46.19**, as well as substrates without β-hydrogens undergoing the process in yields around 10%. The system was also extended to the oxidation of aldehydes to esters. Kanai and coworkers also reported a ternary catalysis method for the allylation of aldehydes **47.1** proceeding through the HAT of allylic C(sp^3^)–H bonds with thiophosphoric imide (TPI) **47.5** (Scheme 47) [261].

**Scheme 48:**
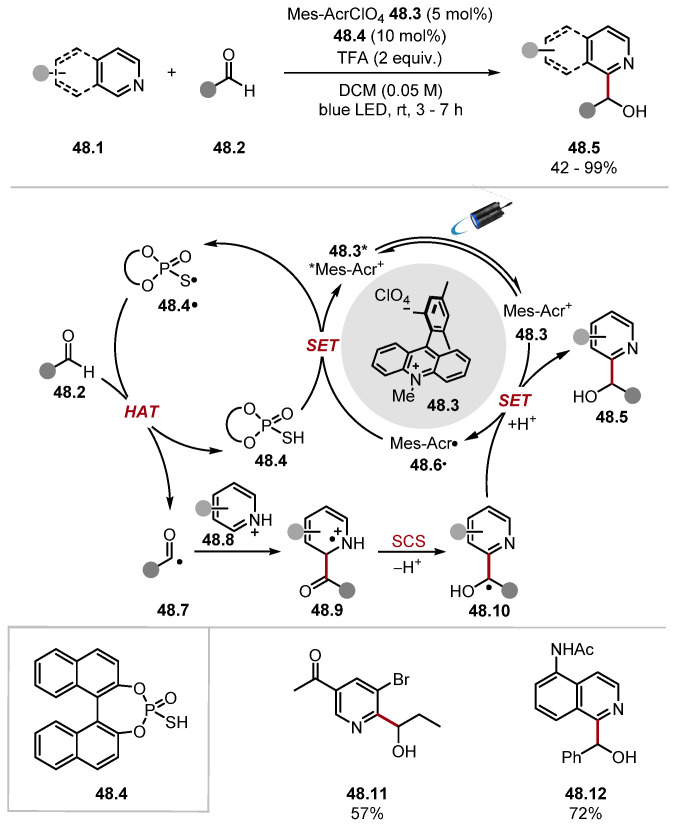
Hydroxyalkylation of *N*-heteroaromatics with aldehydes using thiophosphoric acid **48.4**.

Mechanistically, this method proceeds through the photoexcitation of acridinium photocatalyst **47.4** to generate the strongly oxidising photoexcited catalyst 47.4* (E_1/2_ = +2.12 V vs. SCE in MeCN) [262]. The excited photocatalyst 47.4* can oxidise TPI **47.5**, which also loses a proton to form TPI radical **47.5·**. The TPI radical **47.5·** can abstract a hydrogen atom from a weak allylic C–H bond in **47.2** to generate allyl radical **47.9**, which is intercepted by Cr(II) **47.8** to form Cr(III) complex **47.10**. The allylic Cr(III) complex **47.10** reacts with an aldehyde **47.1**, and the subsequent species undergoes hydrolysis to form *anti*-product **47.6**. A screening of HAT reagents showed that other thiols resulted in no desired product. Notably, the allylic substrates reacted to produce branched products as opposed to linear ones; for instance, product **47.16**. Allyl ether **47.12** reacted to form product **47.13**. The chemoselectivity of the protocol was exceptional with allylic C(sp^3^)–H bonds being functionalised even in the presence of benzylic amines (**47.18**) and benzylic ethers, among other species known to undergo HAT processes. The addition of a chiral INDANE-box ligand **47.19** to the reaction resulted in the formation of the products in high ee values e.g., products **47.20** (88% ee) and **47.21** (72% ee). Subsequently, Kanai used HAT reagent **48.4** for a hydroxyalkylation of *N*-heteroaromatics **48.1**, with aldehydes **48.2** to form hydroxyalkylated products **48.5** (Scheme 48) [263].

**Scheme 49:**
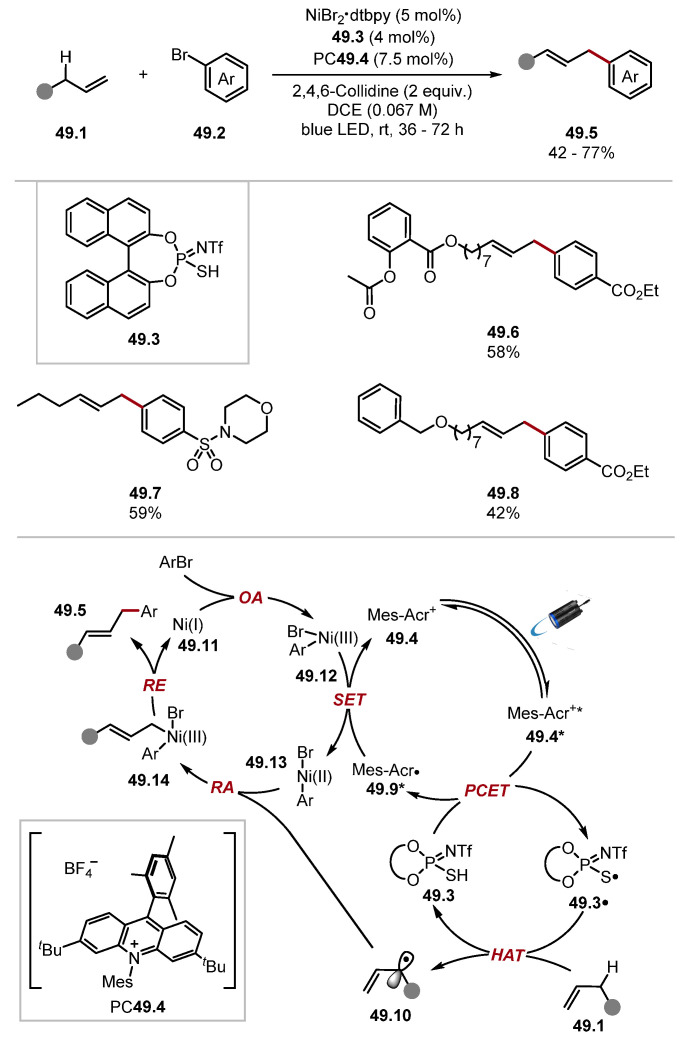
Allylic C(sp^3^)–H arylation through ternary catalysis with thiophosphoric imide **49.3**.

The authors suggested a plausible mechanism for the reaction proceeding through the oxidation of **48.4** with photoexcited MesAcr 48.3* to form a thiyl radical **48.4·**, which abstracts a formyl hydrogen atom [benzaldehyde BDE_C–H_ = 88.7 kcal/mol] to form radical **48.7**. Radical **48.7** undergoes a Minisci-type addition to form intermediate **48.9**, which rapidly undergoes a spin centre shift (SCS) step to form radical **48.10** [264,265,266,267]. The final product **48.5** is delivered upon SET and protonation or PCET. The general protocol tolerated a wide range of functionality on both the aldehyde and *N*-heteroaromatic substrates. Notably, aliphatic aldehydes (product **48.11**) and aromatic aldehydes (product **48.12**) were amenable to this transformation, and common functional groups such as esters, amides, ketones, and halides were tolerated. In 2022, the Glorius group reported an arylation of allylic C(sp^3^)–H bonds proceeding through a triple tandem catalysis protocol combining photoredox and HAT catalysis with nickel-catalysed cross-coupling (Scheme 49) [268]. This methodology showed a good functional group tolerance with respect to both the aryl bromide and allylic substates. Impressively, TPI **49.3** was selective for allylic C(sp^3^)–H bonds over benzyl ether C(sp^3^)–H bonds, as previously noted in Kanai’s work (Scheme 47) [261]. This protocol afforded linear olefin products, rather than branched ones, resulting from a lower energy transition state that was required for the reductive elimination of the linear product. This linear selectivity has been noted in a similar protocol by the Rueping group [144]. The chemoselectivity of the protocol was outstanding, with esters (see product **49.6**), amides, sulfonamides (**49.7**), nitriles, ketones, and *N*-containing heterocycles, including tetrazole among others. Nitro groups were not tolerated. However, low-valent nickel species usually do not tolerate nitro groups due to competitive reductive pathways forming nitroso compounds and inhibiting the catalyst [269,270,271]. The mechanism of this reaction was studied using DFT studies and is believed to progress through an oxidative addition of Ni(I) complex **49.11** with an aryl bromide **49.2** to form a Ni(III) complex **49.12**. This is supported by experimental work by Doyle, who isolated an Ni(III) complex formed by oxidative addition of the Ni(I)-bypyridine complex with aryl bromides [272]. Complex **49.12** is subsequently reduced by MesAcr**· 49.9** to a Ni(II) complex **49.13**, which traps allyl radical **49.10** to form Ni(III) complex **49.14**. The reductive elimination of a linear product is more energetically favourable than a branched product due to steric effects and hyperconjugation in the transition state. Hence, the linear olefin **49.5** is obtained.

**Scheme 50:**
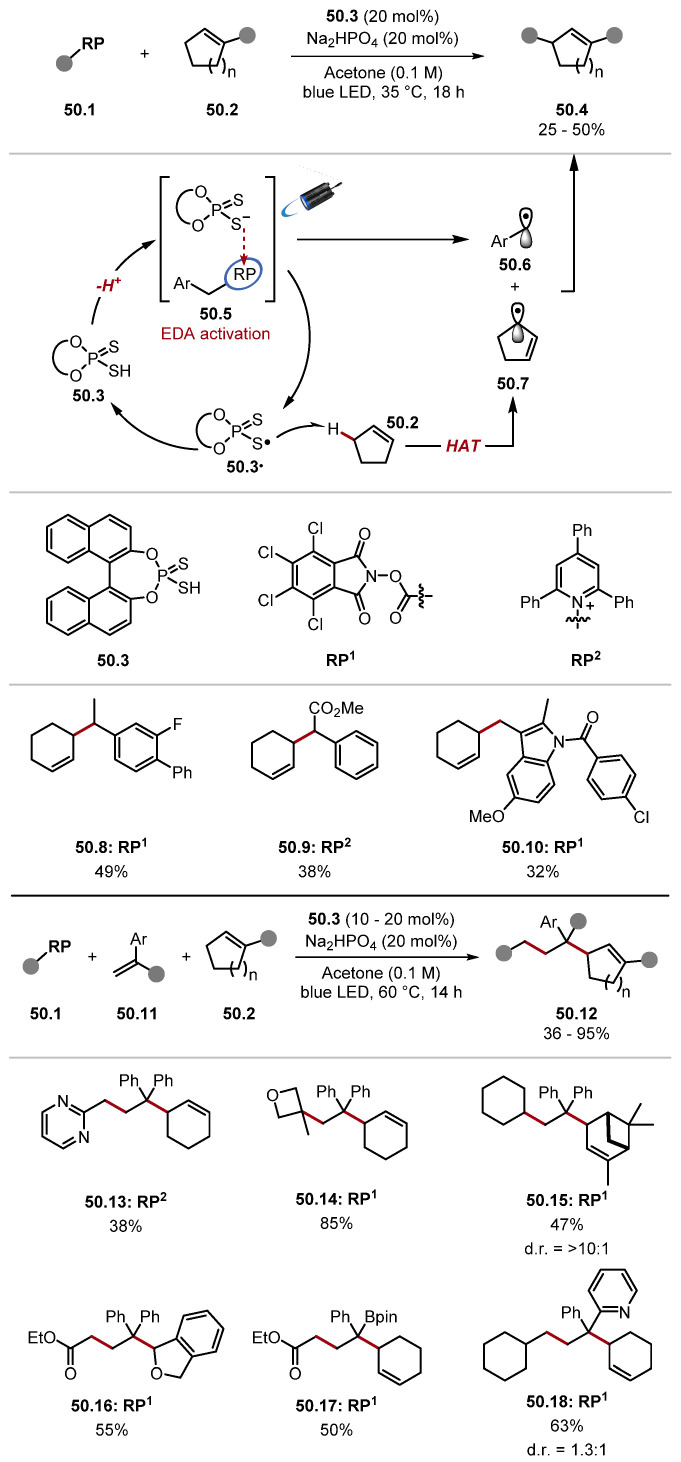
Benzylation of allylic C–H bonds through EDA initiation and HAT using phosphorodithioic acid reagent.

To date, no studies have extensively screened acridinium photocatalysts for the oxidation of BINOL-derived thiophosphoric acids. The decomposition of acridinium photocatalysts containing mesitylene rings through HAT pathways has previously been proposed by Nicewicz and can potentially be alleviated through the use of other acridinium photocatalysts, which were not explored in any works covering BINOL-derived thiophosphoric acid HAT reagents [178,273,274]. Moreover, PC**48.3** and other acridinium photocatalysts without substitution at the core are known to work well for intramolecular processes, but they can decompose through competing radical addition pathways in intermolecular processes [262,275]. BINOL-derived HAT reagents have also been used in processes initiated through EDA complexes. In 2022, the Melchiorre group developed a benzylation of allylic C(sp^3^)–H, which proceeded through HAT using phosphorodithioic acid reagent **50.3** (Scheme 50, top) [276].

The reaction is initiated through EDA complex **50.5** between a thiolate and tetrachlorophthalimides (RP^1^) or Katritzky salts (RP^2^) [230,277]. Upon irradiation, the EDA complex undergoes an intracomplex SET and rapidly fragments to form a benzyl radical **50.6** and thiyl radical **50.3·**, alongside CO_2_ and tetrachlorophthalimide anion (with RP^1^) or 2,4,6-triphenylpyridine (with RP^2^). The thiyl radical **50.3·** can subsequently abstract an allylic hydrogen atom to form an allyl radical **50.7**, which couples with benzylic radical **50.6**. Radical homocoupling products were observed in the reactions supporting this mechanism. The first protocol described in this work focused on directly reacting benzylic radicals with allylic radical to benzylate allylic C(sp^3^)–H bonds. This protocol resulted in the desired products in low-to-moderate yields. However, it showed a good chemoselectivity profile and tolerated both radical precursors RP^1^ and RP^2^.

**Scheme 51:**
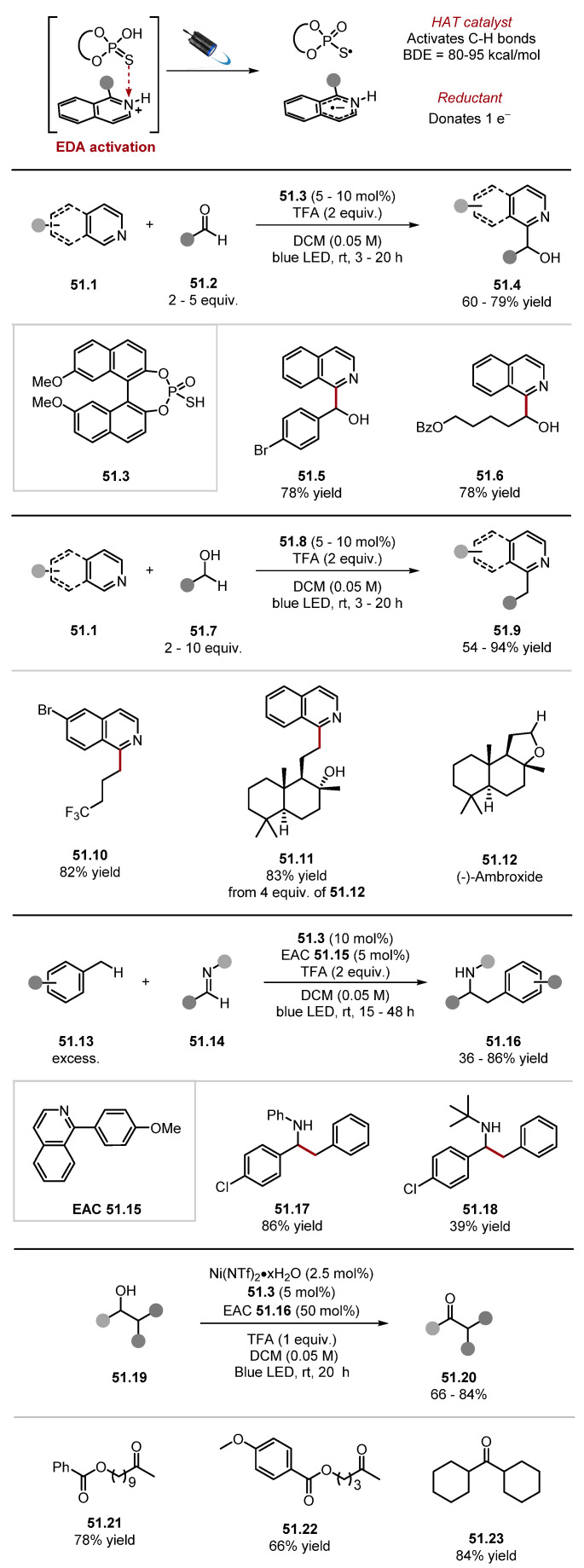
EDA organocatalytic system for HAT processes.

The method was amenable to LSF of pharmaceuticals; for instance, product **50.10** was formed from indomethacin. Another version of this protocol was developed to use kinetically unstable alkyl radicals, which did not react in the initial protocol (Scheme 50, bottom). This was accomplished by trapping the unstable alkyl radical with styrenes **50.11** to form a more stable benzylic radical, which subsequently coupled with an allylic radical **50.7** to form products **50.12**. The second protocol was assessed with a large substrate scope evaluating radical precursors, alkyl radical, styrene species, and allylic species. Pyrimidine benzylic radical formed product **50.13**, and an oxetane tertiary radical formed Product **50.14**. Likewise, a range of allylic precursors and styrene acceptors derived products such as pinene derivative **50.15** and benzyl ether **50.16**, pinacol boronic ester **50.17**, and pyridine **50.18**. The method was also showcased on several natural products and pharmaceuticals, further demonstrating the applicability of this procedure. This work was promptly followed by the Kanai group describing an EDA organocatalytic system that forms an HAT-active thiyl radical upon irradiation by visible light (Scheme 51) [278]. The EDA organocatalytic system was capable of several transformations for proceeding via HAT without an exogenous photosensitiser. This work built upon an observation that the hydroxyalkylation of *N*-heteroaromatics with aldehydes proceeded partially in the absence of a photocatalyst in the group’s previous work (Scheme 48) [278]. Hence, it was assumed that the irradiation of the EDA complex provided a thiyl radical, which can activate C–H bonds. A general protocol for the hydroxyalkylation of *N*-heteroaromatics was described. The protocol provided good-to-excellent yields and improved upon the yields of the previous study [263]. Following this, alcohols were used as alkylating agents for *N*-heteroaromatics in good-to-excellent yields. Interestingly, THF and ambroxide **51.12**-afforded ring opened product **51.11**. The electron-acceptor catalyst (EAC) **51.15** was used to activate HAT reagent **51.3** in situ without a photocatalyst. EAC **51.15** was used for an imine alkylation protocol. The EAC complex was also used for a CAD of secondary alcohols to ketones. This protocol provided the ketones in good yields and proceeded in the presence of weak benzylic ether C(sp^3^)–H bonds.

**Scheme 52:**
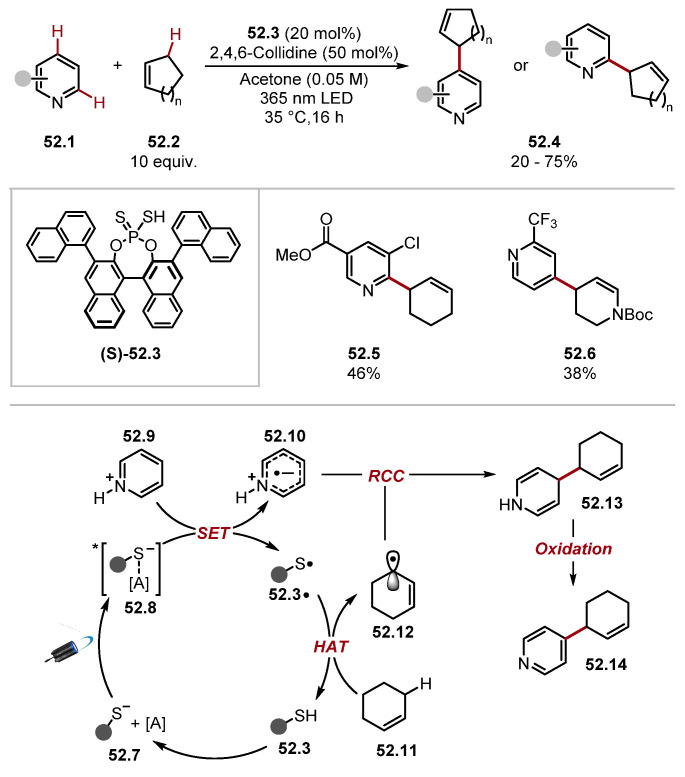
Heteroarylation of allylic C–H bonds through EDA initiation and HAT with phosphorodithioic acid reagent **52.3**.

Melchiorre subsequently developed a heteroarylation of allylic C–H bonds through EDA initiation (Scheme 52) [279]. Mechanistically, the reaction is initiated through an EDA complex **52.8** between the phosphorodithioate of reagent (*S*)-**52.3** and heteroarene **52.1**. The irradiation of complex **52.8** forms pyridyl radical **52.10** and thiyl radical **52.3·**. The thiyl radical **52.3·** abstracts an allylic C–H from **52.11** to form **52.12**. Radical-radical coupling between pyridyl radical **52.10** and allylic radical **52.12** forms intermediate **52.13**, which forms desired product **52.14**. Usually, C4 selectivity at the pyridine substrate was achieved due to the pyridyl radical **52.10** having greater spin density (SOMO) at C4 (as found by DFT and EPR hyperfine splitting). The protocol yielded C6 products when bulky substituents were present in position 3 (e.g., esters and amides).

### 2.3. Oxygen-Based HAT Reagents

As mentioned in Section 2.2 thiols can be deprotonated and oxidised to generate thiyl radicals capable of abstracting weak H atoms (Section 1.2). In contrast, oxygen-centred radicals are typically formed through the homolysis/reduction of weak O–O bonds of peroxides [*^t^*BuCH_2_O–OCH_2_*^t^*Bu BDE_O–O_ = 36.4 kcal mol^−1^] [79] or the oxidation of oxyanions (Section 2.3.1). Oxygen-centred radicals capable of HAT can also be accessed through the excitation of carbonyl compounds with light. In particular, ketones and 1,2-diketones form triplet states capable of HAT [49,53,280,281]. However, this is a method of direct HAT [19,48]. This section describes methods to access O-centred radicals in indirect HAT.

#### 2.3.1. Pyridinium *N*-Oxide HAT Reagents

**Scheme 53:**
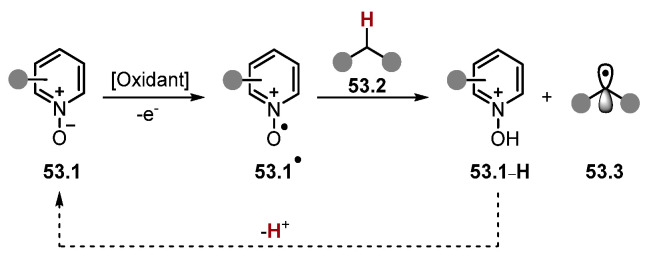
HAT with pyridine *N-*oxides.

Recently, pyridine *N*-oxides have been used as precursors to HAT reagents. Deng and Nicewicz independently reported the use of pyridine *N*-oxides as catalytic HAT reagents with acridinium photoredox catalysts simultaneously (Scheme 53) [282,283]. Pyridine *N*-oxides can act as HAT reagents through the oxidation of pyridine *N*-oxide **53.1** to *N*-oxide cation radical **53.1·** [284,285,286], which form a protonated *N*-oxide **53.1–H** upon HAT [287]. Computational studies by Deng found BDE values in protonated *N*-oxides used in their study ranged from 97.7–111.1 kcal/mol [282], and Nicewicz found that they range from 93–101 kcal/mol [283]. Protonated pyridine *N*-oxides are acidic (*N*-hydroxy-4-methylpyridine p*K*_a_ = 2.43 in DMSO) [288], meaning that pyridine *N*-oxides **53.1** can be used catalytically under basic conditions, similar to other HAT reagents covered within this review. Pyridine *N*-oxides were prone to deactivation by deoxygenation side reactions [283]. However, substituents on pyridine *N*-oxides can be adjusted to suppress this pathway.

**Scheme 54:**
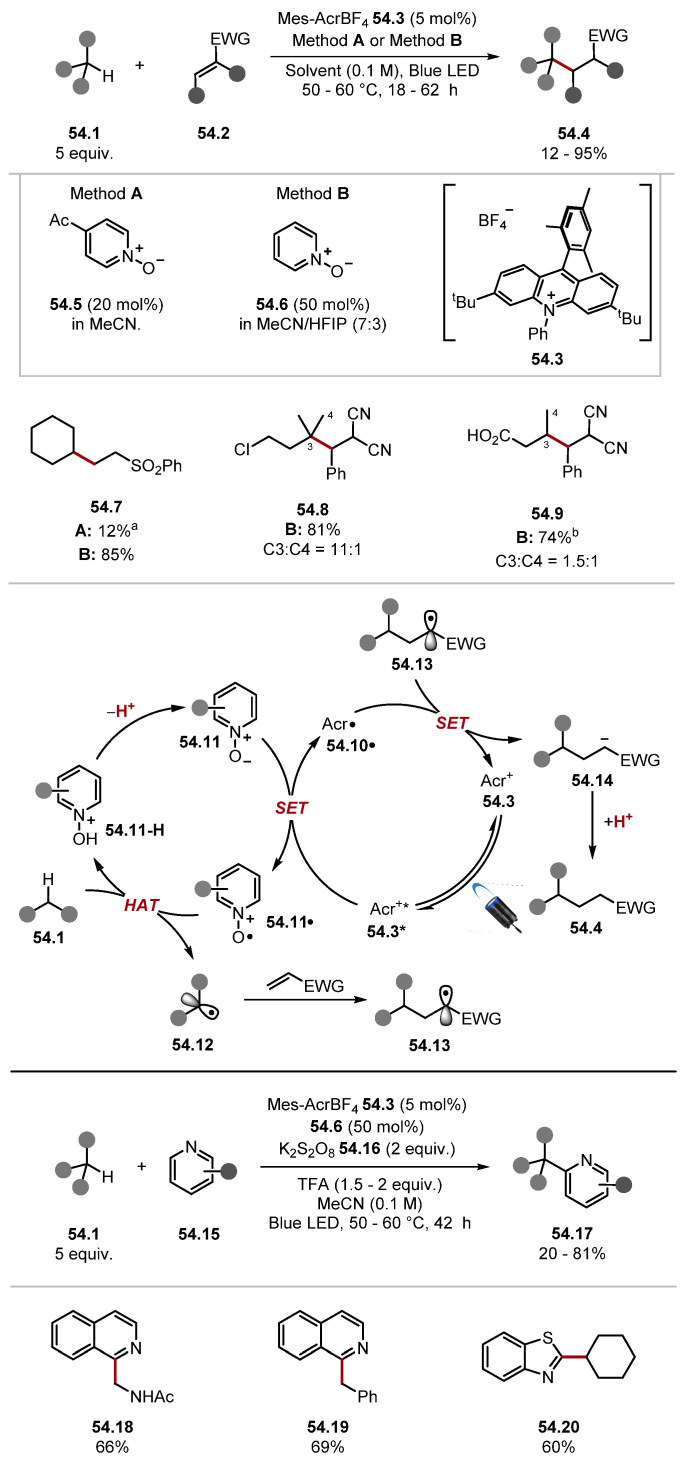
Pyridine *N-*oxide radical cations as HAT reagents 1.

Nicewicz accessed alkyl radicals **54.12** through HAT, with oxyl radicals derived from pyridine *N*-oxides **54.5** and **54.6** for Giese and Minisci pathways (Scheme 54) [283]. Extensive screening identified methods A and B for the alkylation of C–H bonds. Method B requires a higher catalyst loading due to the decomposition of HAT catalyst **54.6** through deoxygenation. For example, method B (HAT catalyst **54.6**) improved the yield of product **54.7** from 12% to 85% yield. Several Giese acceptors were tolerated. However, the substrate scope is limited to easily reducible alkenes due to the reduced form of MesAcrBF_4_ **54.3** having a low oxidation potential. The scope of C–H substrates was wide as products derived from alkanes (**54.7**, **54.8**, and **54.9**), amides, esters, ethers, alcohols, and aldehydes were readily formed. The functional group tolerance was good. For instance, halides **54.8** and carboxylic acid **54.9** provided good yields. The mechanism occurs through the oxidation of pyridine *N-*oxide (E_1/2_ = +1.84 V vs. SCE in MeCN) with photoexcited acridinium photocatalyst 54.3* (E_1/2_ = +2.08 V vs. SCE in MeCN) forming *N-*oxyl radical cation **54.11·**, which is a strong HAT reagent capable of oxidizing strong C–H bonds [pyridine *N-*oxide **54.6** BDE_O–H_ = 99 kcal/mol^−1^ versus cyclohexane BDE_C–H_ = 99 kcal/mol^−1^]. The resulting alkyl radical **54.12** is trapped with a Giese acceptor **54.2** forming a radical adduct **54.13**. The radical adduct is reduced to an anion by Acr**^•^ 54.3** (MesAcr **54.3** E_1/2_ (PC/PC**^•−^**) = −0.59 V versus SCE in MeCN versus E_1/2_ (**•**CH_2_CO_2_Et/−CH_2_CO_2_Et) = −0.63 V versus SCE in MeCN) and subsequently protonated [179,180]. Nicewicz also used pyridine *N-*oxide **54.6** for Minisci-type reactions. The functional group tolerance of this reaction was similar to the alkylation protocol with heteroarene products arising from amides (**54.18**), toluene (**54.19**), and cyclohexane (**54.20**) were formed products in moderate-to-good yields.

**Scheme 55:**
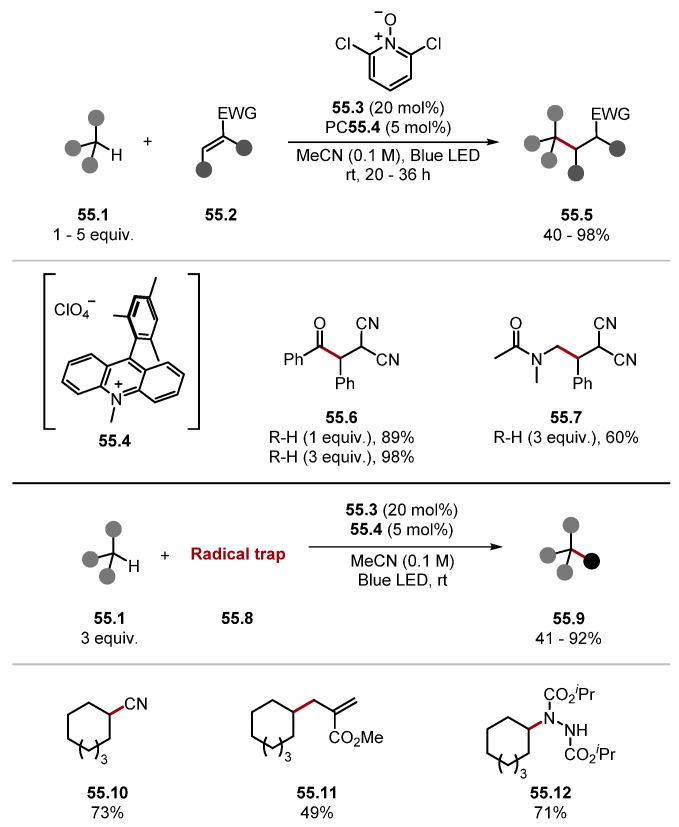
Pyridine *N-*oxide radical cations as HAT reagents 2.

Deng showed the functionalisation of various C–H substrates using pyridinium *N-*oxide **55.4** (Scheme 55) [282]. Products were successfully derived from aldehydes (**55.6**), amides (**55.7**), alcohols, ethers, and benzylic substrates, which were derivatised successfully. Impressively using one equivalent of C–H substrate only led to a minor decrease in yield. Moreover, various radical traps were deployed in this process to form **55.10** and **55.11**, with diisopropyl azodicarboxylate (DIAD) forming **55.12**.

**Scheme 56:**
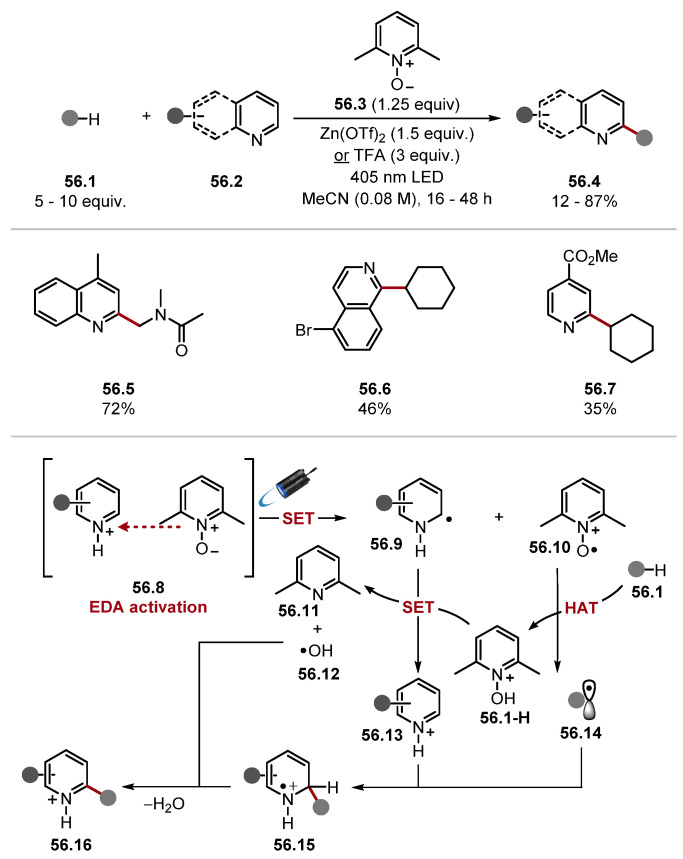
Pyridine *N*-oxide cation radicals as HAT reagents in a Minisci-style reaction.

Gryko showed that an EDA complex **56.8** between pyridine *N*-oxides **56.3** and Brønsted or Lewis acid-activated azines can generate pyridine *N*-oxide cation radicals **56.10·** for a subsequent HAT process (Scheme 56) [289]. The radicals generated through HAT were harnessed in Minisci-style reactions with various heteroarenes **56.2** activated under acidic conditions [127]. Various HAT substrates reacted well; for instance, cycloalkanes, alkenes, ethers, amides, and carbamates, as seen in products **56.5**–**56.7**. The method also tolerated numerous heterocycles. Notably, halides **56.6** and esters **56.7** reacted in moderate yields. The reaction is believed to be initiated through an EDA complex **56.8**, which upon irradiation by visible light provides reduced heteroarene **56.9** and *N*-oxide cation radical **56.10·**. *N*-oxide cation radical **56.10·** can abstract hydrogen from strong C–H bonds to form alkyl radical **56.14**. The alkyl radical is trapped by an acid-activated heteroarene **56.13** to form radical adduct **56.15**. The radical adduct **56.15** delivers the protonated product **56.16** after formally losing a hydrogen atom.

#### 2.3.2. Peroxide HAT Reagents

Peroxides have historically been used as oxidants and continue to be a reliable and robust option for indirect HAT and oxidation [112,290,291,292,293]. Alkyl peroxides function under acidic conditions, and peroxides with higher degrees of substitution are thermally stable [112]. Peroxides generate O-centred radicals through the homolysis of weak O–O bonds typically induced by light, heat, or a photosensitiser [*^t^*BuCH_2_O–OCH_2_*^t^*Bu BDE_O–O_ = 36.4 kcal mol^−1^] [79,112]. Alternatively, peroxides can generate O-centred radicals through the reduction of O–O bonds mediated by a metal complex or photosensitiser to form a oxyanion and oxyradical (Scheme 7) [112]. The resulting O-centred radicals can abstract hydrogen atoms to form moderate-to-strong O–H bonds depending on the peroxide used [*^t^*BuOOH BDE_O–H_ = 89.4 kcal/mol and *^t^*BuOH BDE_O–H_ = 105.1 kcal/mol and MeC(O)O-H BDE_O–H_ = 106.4 kcal/mol] [79].

**Scheme 57:**
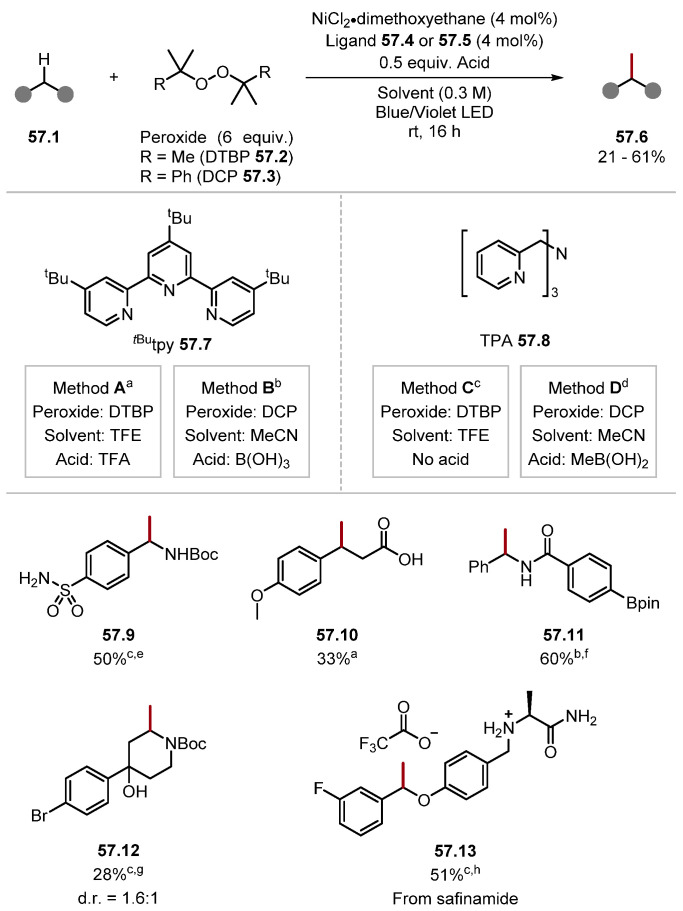
Methylation of C–H bonds through a combination of HAT using oxyl radicals and Ni-catalysed cross-coupling. ^a^ Method A was used. ^b^ Method B was used. ^c^ Method C was used. ^d^ Method D was used. ^e^ 0.5 equiv. MeB(OH)_2_ used as acid. ^f^ 1:1 MeCN:DMSO used as solvent. ^g^ 0.15 M concentration. ^h^ 4 equiv. of DCP, and MeCN was used as solvent.

In 2021, Stahl used peroxides (dicumyl peroxide or di-*tert*-butyl peroxide) to methylate C–H bonds in the presence of a nickel catalyst and ligand with visible light (Scheme 57) [294]. In this method, peroxides filled two roles: the HAT reagent and a source of methyl radical (after β-methyl scission) [295]. To this end, a 4-to-6-fold excess of peroxide reagent was used, and polar solvents were used (TFE or MeCN). Polar solvents produced superior results due to faster β-methyl scission, which likely arises from the increased solvation of the ketone by-product [296,297]. The nickel catalyst mediated the coupling of two alkyl radicals. In its absence, greater amounts of side-products were formed, and selective radical-radical coupling did not occur. The method afforded methylated products in low-to-fair yields. However, these are yields of monomethylated products after purification. Therefore, in the context of late-stage functionalisation, this is an excellent result.

**Scheme 58:**
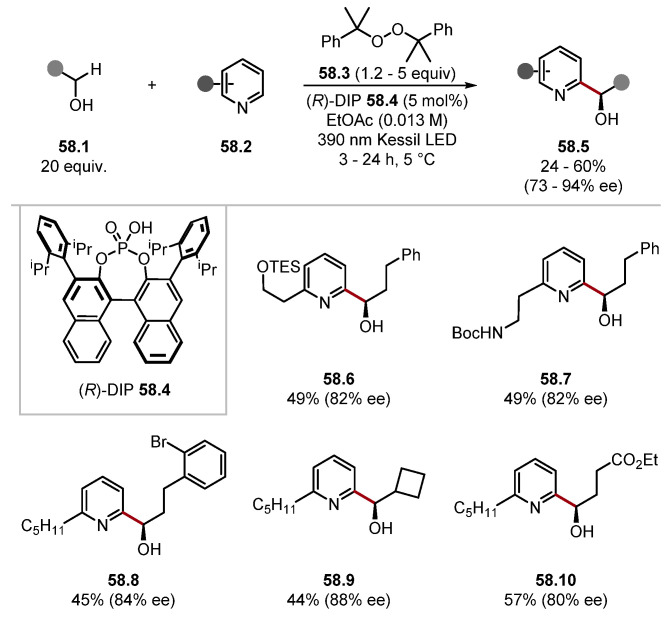
Enantioselective Minisci reaction with alcohols through HAT with dicumyl peroxide.

**Scheme 59:**
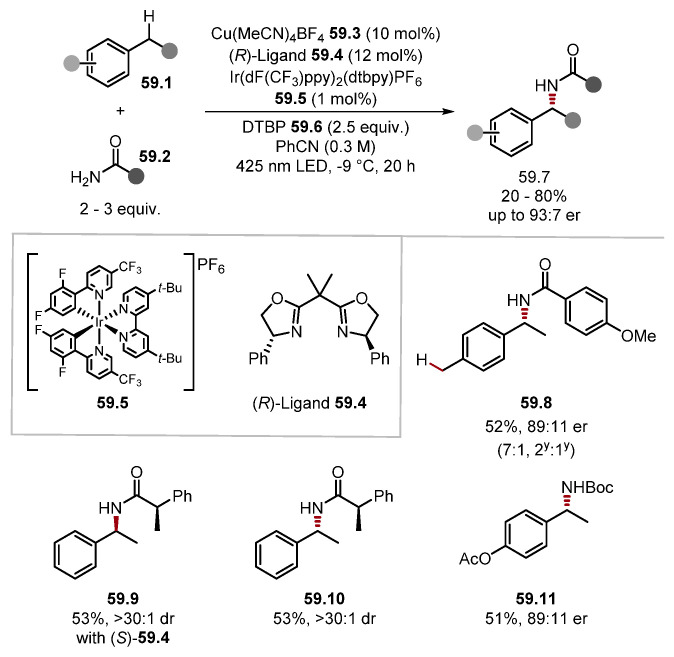
Enantioselective amidation using di-*tert*-butyl peroxide **59.6** for HAT with photoredox and copper-tandem catalysis.

**Scheme 60:**
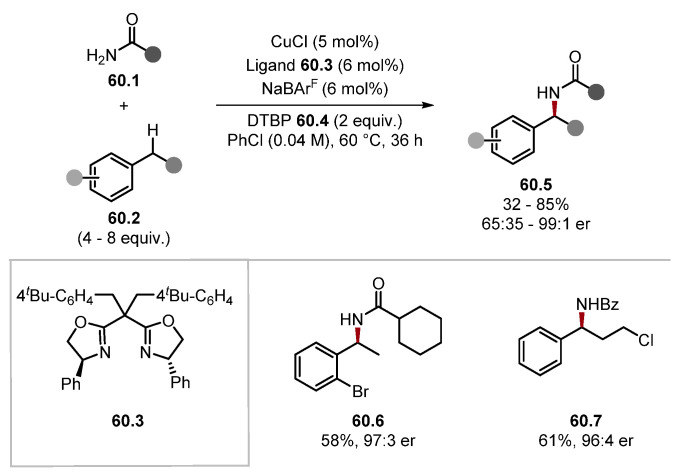
Enantioselective amination of benzylic C–H positions using di-*tert*-butyl peroxide and a Cu-catalyst.

The scope of the reaction was wide, and numerous functional groups were tolerated; for instance, sulfonamides (**57.9**), carboxylic acids (**57.10**), amides (**57.11**), alcohols (**57.12**), esters, ketones and amines, and carbamates (**57.12**). Notably, the abstraction at amine α-C–H bonds could be suppressed under acidic conditions as seen in safinamide derivative **57.13** [29]. Overall, this method is a landmark in terms of synthetic tools available for medicinal chemistry to explore the magic methyl effect [9,298,299]. Phipps recently disclosed an enantioselective Minisci reaction with alcohols and chiral phosphoric acid proceeding through HAT with dicumyl peroxide **58.3** under blue-light irradiation (Scheme 58) [300]. Notably, no photocatalyst was used, echoing Phipps’s previous hydrogen atom transfer-driven enantioselective Minisci reaction with amides [281]. DFT studies showed the enantioselectivity in this protocol arises from a complex of the protonated azine with a chiral phosphoric acid (CPA), which forms H-bonds with the incoming alcohol similarly to the group’s previous work [281,301]. Despite the protocol yielding products in only moderate yields (up to 60%), the enantiomeric excesses were excellent, with up to 94% ee. Moreover, the functional group tolerance of the protocol was good with numerous alcohols and azines reacting in moderate yields with excellent ee values. Kramer reported an enantioselective amidation and amination of benzylic C–H bonds using copper with BOX ligand **59.4** and photoredox tandem catalysis with di-*tert*-butyl peroxide (DTBP) **59.6** (Scheme 59) [302]. Amides and carbamates reacted in good yields. The protocol showed good functional group tolerance, and the enantioselectivity could be changed by changing the ligand’s stereochemistry (**59.9** and **59.10**). The deprotection of Boc-protected benzylic amine **59.11** allows for a two-step C–H amination. Zhou developed a similar transformation under thermal conditions using DTBP **60.4** and a Cu catalyst with BOX ligand **60.3** (Scheme 60) [303]. Only amides were showcased in the protocol, but both aryl and alkyl amides reacted in good yields.

**Scheme 61:**
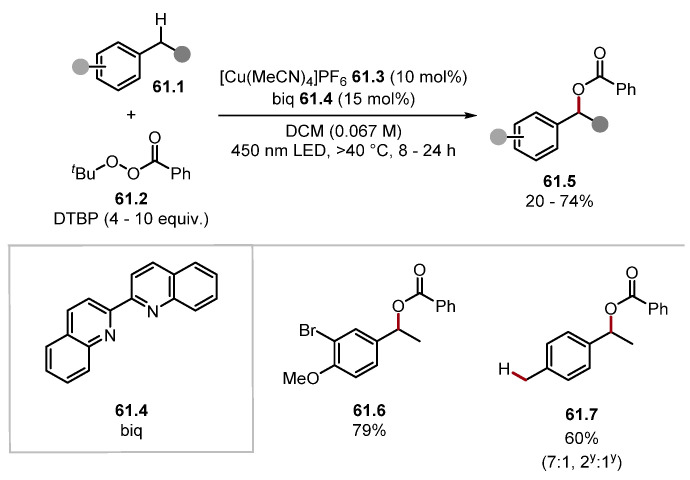
Esterification of benzylic C–H bonds using a photoactive Cu-catalyst and *tert*-butyl peroxybenozate **61.2**.

Stahl used a *tert*-butyl peroxybenzoate (TBPB) **61.2** with a photoactive copper catalyst for a benzylic C–H esterification (Scheme 61) [304]. The hydrolysis of the products allowed for a two-step C–H hydroxylation drawing a parallel with a recent direct hydroxylation of C–H bonds with nitroarene by Parasram [72].

**Scheme 62:**
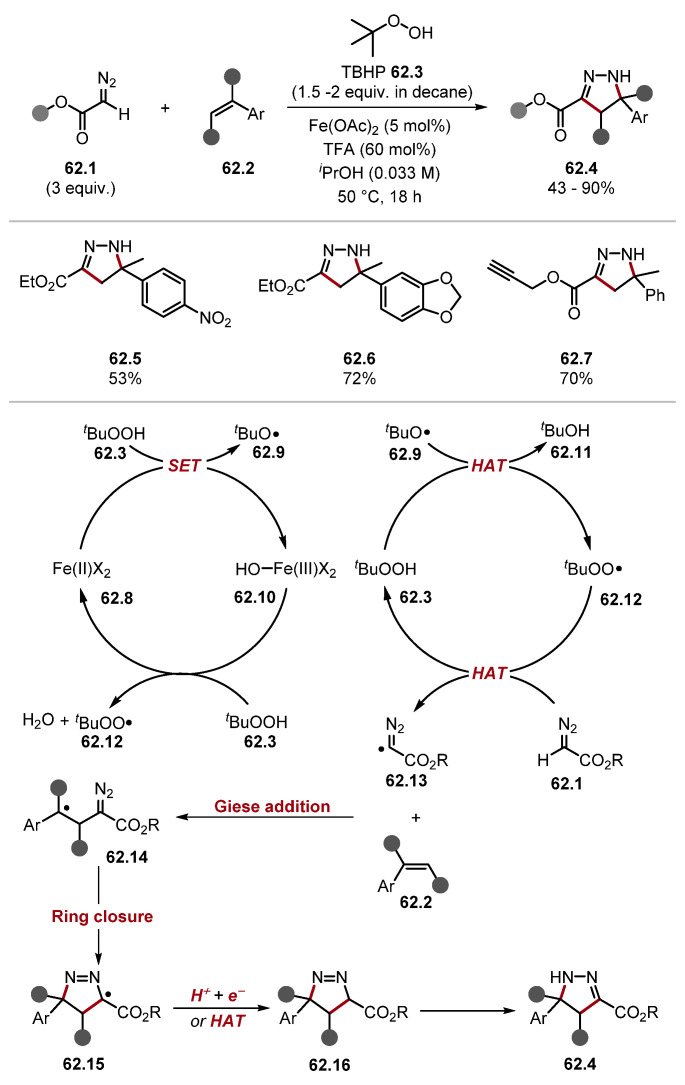
Formation of pyrazolines from styrenes **62.2**, and diazoacyl radical **62.13** formed through HAT with peroxyl radical **62.12**.

**Scheme 63:**
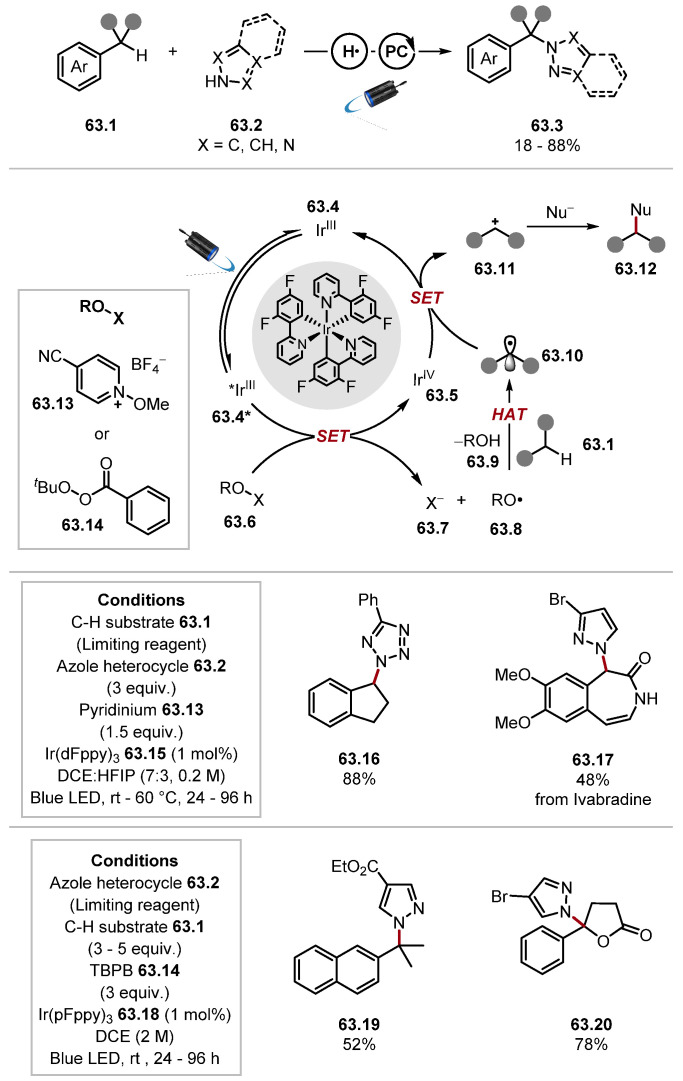
Azolation of benzylic C–H bonds through a ORPC using *N*-alkoxypyridinium salts or *tert*-butyl peroxybenozate for HAT.

**Scheme 64:**
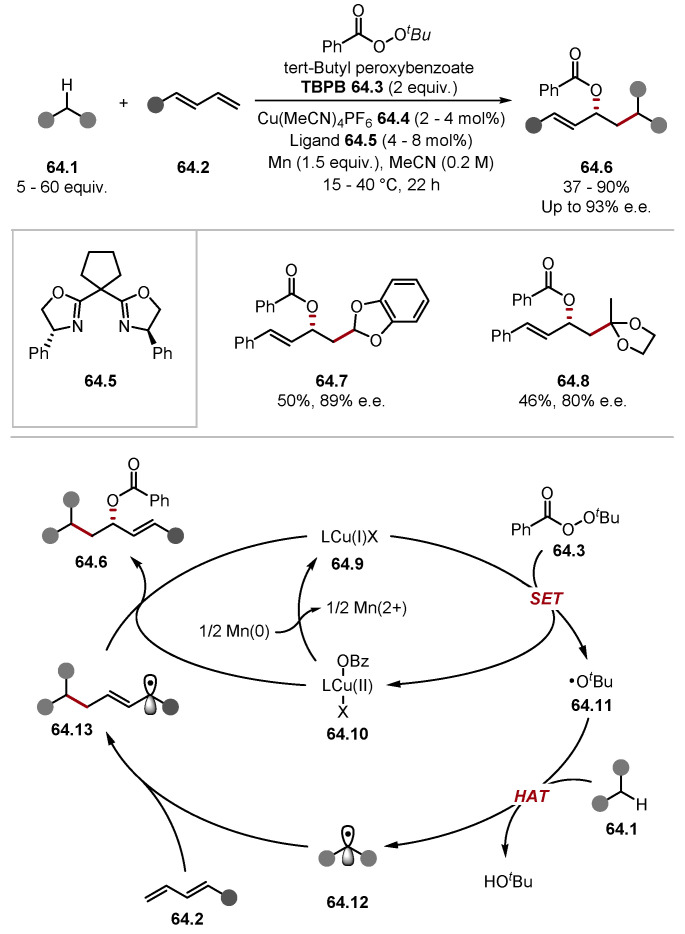
Asymmetric 1,2-oxidative alkylation of conjugated dienes mediated by HAT with *tert*-butyl peroxybenozate and Cu-catalysis.

Doyle used TBHP **62.3** to form diazoacyl radicals **62.13** from α-diazo esters **62.1**. Diazoacyl radicals **62.13** react with styrenes **62.2** to form pyrazolines **62.4** (Scheme 62) [305]. Only styrenes **62.2** and α-diazo esters **62.1** were used as substrates. However, the functional group tolerance of the reaction was good with products formed bearing nitro groups (**62.5**), 1,3-benzodioxole (**62.6**), and alkynes (**62.7**) in good yields. The reaction occurs through reductions of TBHP **62.3**, with an iron (II) complex **62.8** to form oxyl-radical **62.9** and an iron (III) complex **62.10**. TBHP **62.3** can form the peroxyl-radical **62.12** upon the oxidation of TBHP **62.3** with Fe(III) **62.10**. HAT to *tert*-butoxy radical **62.9** from TBHP **62.3** also delivered a peroxyl radical **62.12** [*^t^*BuOH BDE_O–H_ = 105.1 kcal/mol versus *^t^*BuOOH BDE_O–H_ = 89.4 kcal/mol] [79]. Peroxyl-radical **62.12** can abstract a hydrogen atom from α-diazo ester **62.1** to form diazoacyl radicals **62.13**, which undergo a Giese addition with Styrenes **62.2** to form a benzylic radical adduct **62.14**. The benzylic radical adduct **62.14** rapidly cyclises to form radical **62.15**, which is quenched by gaining a hydrogen atom via HAT or through a reduction and subsequent protonation affording an intermediate **62.16**. Intermediate **62.16** yields the pyrazoline **62.4** final product after tautomerisation. Musacchio used TBPB **63.14** and *N*-alkoxypyridinium salts **63.13** in a C–H azolation of benzylic and allylic C–H bonds (Scheme 63) [306]. *N*-alkoxypyridinium salts **63.13** are known to generate alkoxyl radicals in situ upon reduction [307]. *N*-alkoxypyridinium salts have been used to trigger HAT events from C–H, P–H, and Si–H bonds [308,309,310,311,312,313]. In this protocol, alkoxyl radicals were used to abstract benzylic C–H bonds to form a benzylic radical. The benzylic radical was further oxidised to a carbocation, which reacted with numerous azoles. Secondary benzylic positions were derivatised using the C–H substrate as a limiting reagent. However, the functionalisation of tertiary positions used a three-fold excess of the C–H substrate. Musacchio has developed mechanistically similar protocols incorporating a range of nucleophiles into C–H bonds (e.g., F, C, O, N, Br, Cl) [314,315]. Gong described an asymmetric 1,2-oxidative alkylation of conjugated dienes **64.2** to form allylic esters **64.6** (Scheme 64) [316]. This method used *tert*-butyl peroxybenzoate **64.3** as a HAT reagent to form oxyl radical **64.11** through a reduction with Cu(I) **64.9**. Oxyl radical **64.11** abstracts hydrogen from various C–H substrates to form alkyl radical **64.12**. Alkyl radicals are rapidly trapped by dienes to form allylic radicals **64.13** [317]. The allylic radical **64.13** then reacts with Cu(II) complex **64.10** to form the desired product **64.6**, reforming Cu(I) catalyst **64.9**.

#### 2.3.3. Miscellaneous Oxygen HAT Reagents

**Scheme 65:**
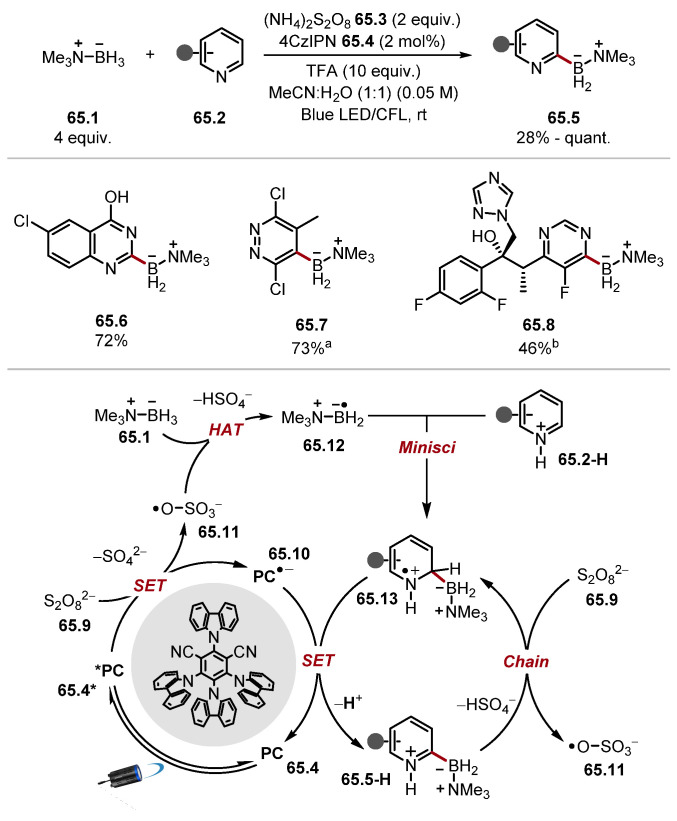
Borylation of azines **65.2** using amino boranes **65.1**. ^a^Reaction without acid, instead using K_2_CO_3_ (2 equiv.). ^b^Reaction used Ru(bpy)_3_(PF_6_)_2_ (2 mol%) and AcOH (10 equiv.).

Many other O-centred radicals can also be used for HAT. Persulfates are also common HAT reagents [318,319,320]. In 2021, Leonori used ammonium persulfate **65.3** to form aminoboryl radical **65.12** for a Minisci-style borylation of azines **65.2** with aminoborane **65.1** (Scheme 65) [321]. The protocol was amenable to many azines and showed excellent functional group tolerance with alcohols **65.6**, halides **65.7**, and nitriles, among others reacting well. The protocol was also showcased on pharmaceuticals; for instance, Voriconazole was borylated to product **65.8**. The reaction mechanism occurs through the oxidation of persulfate **65.9** to the O-centred radical **65.11**. The O-centred radical **65.11** is electrophilic and can abstract a hydridic hydrogen atom from a B–H bond in amino borane **65.1** to form an aminoboryl radical **65.12·**. Aminoboryl radical **65.12·** is a highly nucleophilic radical and rapidly adds to azine **65.2-H** through a Minisci-style pathway to form radical adduct **64.13**, which formally loses a hydrogen atom through chain contribution or PCET with the oxidised photocatalyst to form a borylated product **65.5-H**.

**Scheme 66:**
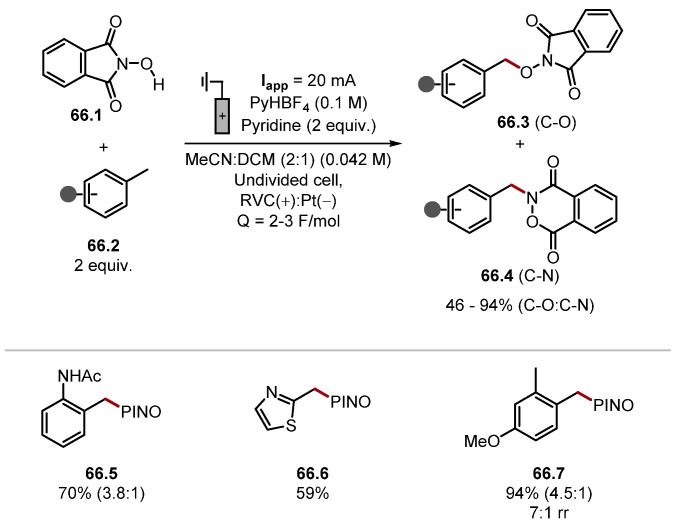
PINOlylation of methylarenes **66.2**, with NHPI **66.1** under cathodic oxidation.

**Scheme 67:**
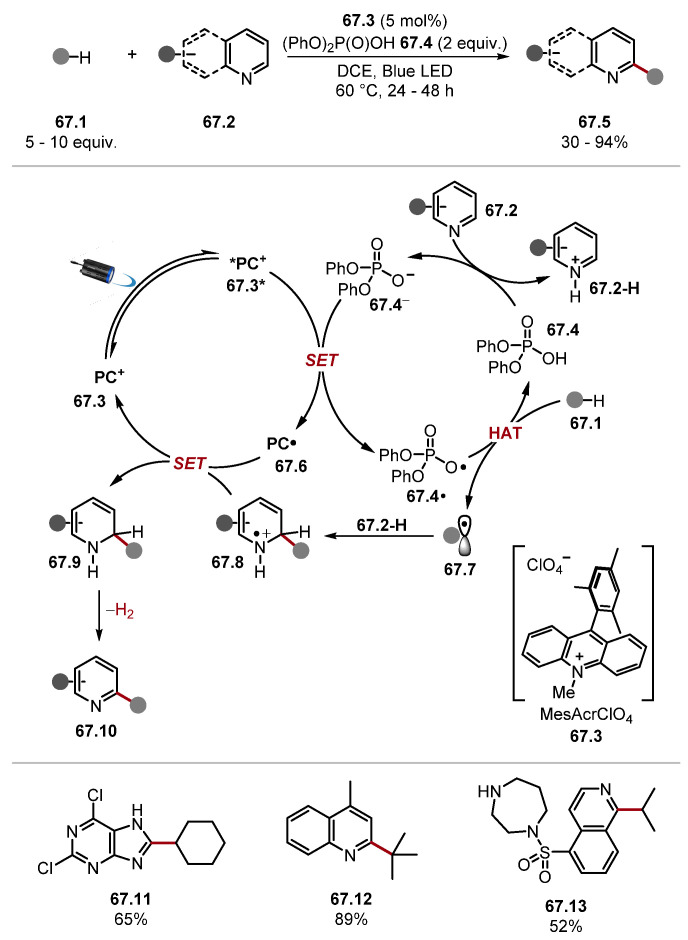
Minisci-style cross-dehydrogenative coupling between heteroarenes **67.2** and C–H substrates **67.1** through HAT with diphenyl phosphate **67.4**.

In recent years, *N*-hydroxyphthalimide (NHPI) **66.1** has been explored extensively as a radical initiator, oxidant, and/or HAT reagent [322,323,324]. Phthalimide-*N*-oxyl radical (PINO) **66.1·** can abstract hydrogen from weak, typically benzylic or allylic, C–H bonds to form NHPI **66.1** [NHPI BDE_O–H_ = 88.1 kcal/mol compared with cyclohexene BDE_C–H_ = 81.0 kcal/mol or toluene BDE_C–H_ = 88.5 kcal/mol] [79,325]. *N*-hydroxyphthalimide **66.1** can formally lose a hydrogen atom to form phthalimide-*N*-oxyl radical **66.1·**. Practically, this can be accomplished through oxidation followed by deprotonation. The oxidation potential of *N*-hydroxyphthalimide **66.1^−^** is quite high (E_1/2_ = +1.44 V vs. SCE in MeCN). However, the addition of pyridine lowers the oxidation potential significantly (E_1/2_ = +0.78 V versus SCE in MeCN, with two equivalents of pyridine) [326,327]. In 2022, Stahl generated phthalimide-*N*-oxyl radical **66.1·** anodically from NHPI **66.1** in the presence of two equivalents of pyridine for a PINOylation of methylarenes **66.2** (Scheme 66) [328]. The PINOylated products **66.3** were readily converted to benzylic alcohols or benzaldehydes under photocatalytic conditions in subsequent steps. Interestingly, a mixture of C–O **66.3** and C–N **66.4** coupled products was formed. However, the use of more hindered secondary radicals resulted in almost no C–N product forming. The method showed good functional group tolerance forming products bearing ketones, halides, amides **66.5**, and numerous heterocycles, such as product **66.6**. product **66.7** was formed in a 7:1 ratio with its regioisomer. Additionally, a robustness screening had shown the method developed to be quite insensitive to many different functional groups and heterocycles. Diphenyl phosphate **67.4**, and other phosphates, are seldomly used in HAT protocols but remain a viable option due to the strength of O–H bonds [diphenyl phosphate **67.4** BDE_O–H_ = 102.4 kcal/mol] and low p*K*_a_ (p*K*_a_ = 2.4) [329,330]. However, the relatively high oxidation potential of dialkyl/diaryl hydrogen phosphates limits wider relevance (**67.4** E_1/2_ ((RO)_2_P(O)O**·**/(RO)_2_P(O)O^−^) = +1.50 V vs. SCE in MeCN) [330]. In 2022, Wu and Deng developed a Minisci-style cross-dehydrogenative coupling between heteroarenes and various C–H substrates under photoredox–HAT conditions in a stop-flow microtubing reactor (Scheme 67) [331]. The protocol used strongly oxidised MesAcrClO_4_ **67.3** as a photocatalyst (E_1/2_ = +2.06 V versus SCE in MeCN) to oxidise a phosphate anion **67.4**^−^ (E_1/2_ ((PhO)_2_P(O)O**·**/(PhO)_2_P(O)O^−^) = +1.59 V versus SCE in MeCN) [331,332]. This forms the reduced **67.6** and phosphatyl radical **67.4·**, which abstracts a hydrogen from substrate **67.1** to form alkyl radical **67.7** and reform phosphate **67.4**. The alkyl radical was trapped by a protonated azine heterocycle **67.2** to form radical adduct **67.8**. The radical adduct **67.8** can then be reduced to form Intermediate **67.9** (and reform PC**67.3**), which rapidly loses H_2_ to rearomatise and form the functionalised heteroarene **67.10**. The prepared products from numerous C–H substrates, including ethers, alcohols, ethane, aldehydes, and cycloalkanes, such as cyclohexane product **67.11**. Aldehydes with secondary and tertiary substitution on α-carbon atoms formed decarbonylated products, such as product **67.12** from pivaldehyde. This is due to fast rates of decarbonylation for the corresponding acyl radicals [149]. The functional group tolerance of the reaction was good, with products being formed including numerous heterocycles, halides (see product **67.11**), amines (**67.13**), and ketones, among others reacting well. The method was showcased on numerous drug molecules, such as Fasudil **67.13**.

### 2.4. Carbon and Boron HAT Agents

#### 2.4.1. Carbon HAT Reagents

C-centred radicals are commonly used in intramolecular HAT processes, but they are rare in intermolecular HAT processes [333]. There are several reasons for this. The first reason is the historical difficulties associated with forming C-centred radicals [24]. In recent years, this limitation has been overcome with modern methods of radical generation and the use of various radical precursors [45,48,76,143,275,277,334,335,336,337,338]. Another reason is an inherently poor polarity match in the transition state between a C-centred radical and C–H bond [333], compared with the HAT of hydridic hydrogen atoms using N/S/O-centred radicals [32,87]. The limited examples of intermolecular HATs with C-centred radicals possess very favourable thermodynamic effects, as species that form bonds with very high C–H BDE values are used, or hydrogen atoms are abstracted from weak C–H bonds. Additionally, C(sp^2^) radicals are typically used as they possess greater electrophilic character than C(sp^3^) radicals [339]. These trends are clear looking at examples from the literature using highly reactive aryl radical [benzene BDE_C–H_ = 112.9 kcal/mol] or methyl radical [methane BDE_C–H_ = 105.0 kcal/mol] for HAT processes [79,340,341,342,343,344]. Other reported C-centred HAT reagents are electrophilic. Hence, they provide a better polarity match in the transition state and/or take place intramolecularly [19,333].

**Scheme 68:**
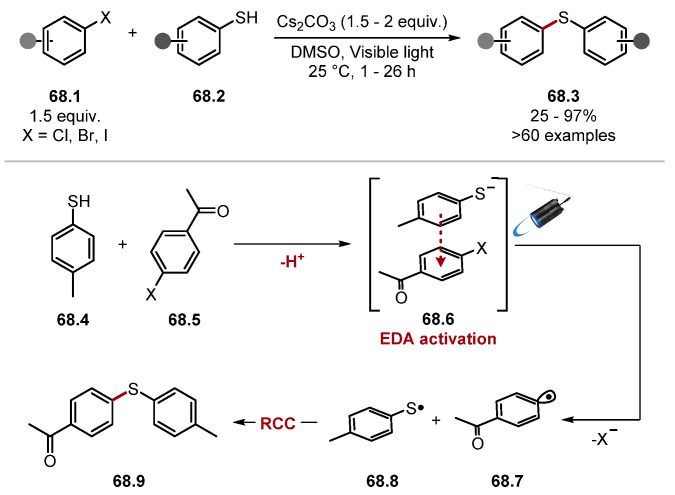
C–S cross-coupling through EDA activation and visible light.

Miyake showed that aryl halides **68.1** and thiophenols **68.2** formed C–S cross-coupled product **68.3** under basic conditions and irradiation by visible light (Scheme 68) [232]. Mechanistic studies suggested that this reaction occurred through an EDA complex **68.6**, generating an aryl radical **68.7** and S-radical **68.8**. These species subsequently underwent a radical–radical cross-coupling [230,231]. The mechanistic implications of Miyake’s findings inspired several groups to investigate aryl halides as precursors to aryl radicals for HAT processes.

**Scheme 69:**
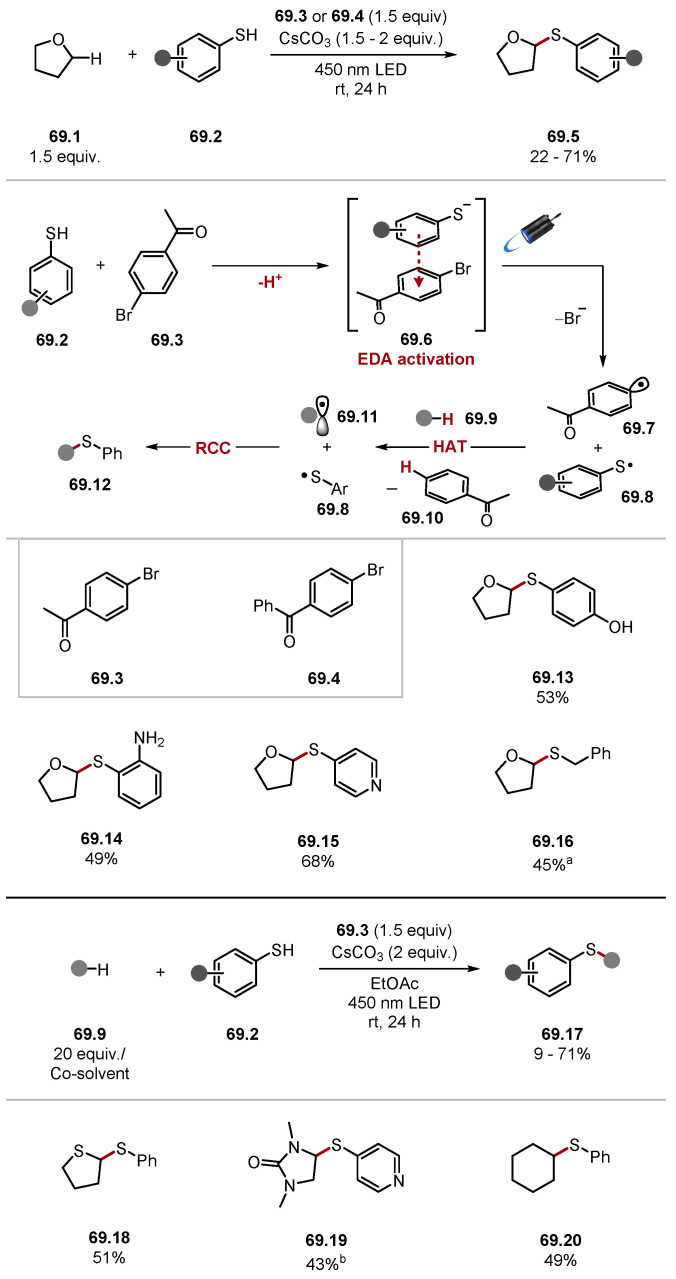
C–S cross-coupling through EDA activation to form aryl radicals. ^a^ Additional step to reduce residual ketone with NaBH_4_ and MeOH. ^b^ 1 mL of C–H substrate (0.1 M w.r.t thiol).

**Scheme 70:**
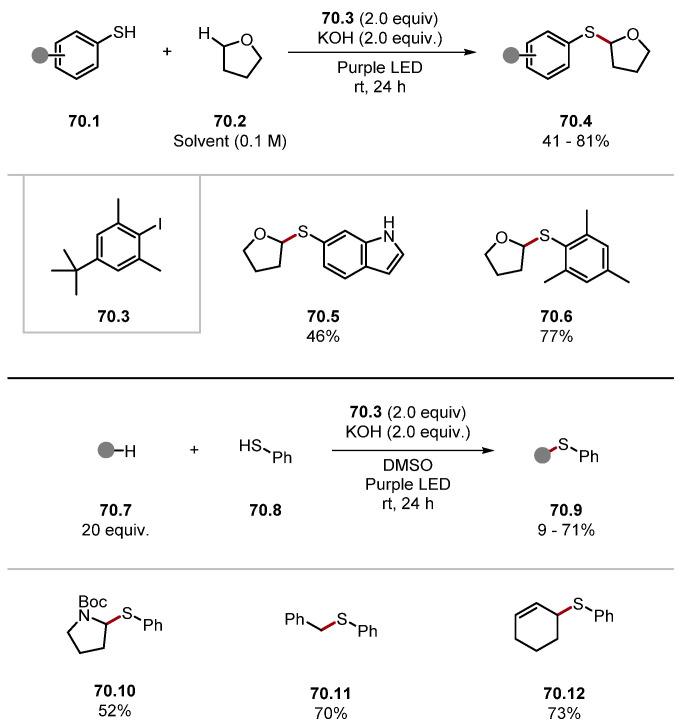
C–S cross-coupling through EDA activation to form aryl radicals for HAT.

In 2021, Akiyama used aryl halides **69.3** and **69.4** as pro-radicals in a HAT-driven cross-coupling of C–H substrate **69.9** (such as THF **69.1**) and thiophenol **69.2** for a C–S (Scheme 69) [345]. The mechanism of the reaction occurs through the formation of the EDA complex **69.6** upon deprotonation of thiophenol **69.2**. Upon irradiation with blue LEDs, the EDA complex fragments expel a bromide ion, thiophenyl radical **69.8**, and aryl radical **69.7**. As mentioned previously, aryl radicals **69.7** abstract hydrogen atoms to form alkyl radical **69.11**, which can selectively react with S-radical **69.8** to form cross-coupled product **69.12**. *p*-Bromoacetophenone **69.3** and *p*-bromobenzophenone **69.4** were used as aryl radical precursors. The protocol displayed good chemoselectivity as halides, while alcohol **69.13**, amine **69.14**, and pyridine **69.15** reacted well. Benzylic thiol **69.16** was tolerated. However, alkyl thiols were inactive in the reaction, which was likely due to the requirement for π–π interactions in the EDA complex. Numerous substrates reacted well under adapted conditions, forming products such as tetrahydrothiophene (**69.18**), urea (**69.19**), and cyclohexane (**69.20**). Shortly after, Xia published a similar method utilizing iodoarene **70.3** as an aryl pro-radical (Scheme 70) [346]. An assessment of the substrate scope showed excellent functional group tolerance. Several heterocycles, such as indole **70.5**, thiophene, furan, and pyridine were prepared. The reaction was also tolerant of steric hindrance, as seen in mesityl product **70.6**. An adjusted version of the method was used to assess the scope of C–H substrate. Again, the method was competent on numerous substrates, forming products such as carbamates (**70.10**), benzylic product (**70.11**), and allylic substrates (**70.12**), among many others. In contrast to Akiyama’s approach, the pro-radical iodoarene **70.3** contains electron-donating groups. Notably, less sterically encumbered iodoarenes provided greater amounts of the C–S cross-coupled product, as per Miyake’s work (Scheme 68). Using iodoarene **70.3**, only 5% of the C–S product (such as **68.3**) was seen. By comparison, around 14% of a similar side-product was seen in Akiyama’s screening experiments. In both works, this side product was easily removed by column chromatography.

**Scheme 71:**
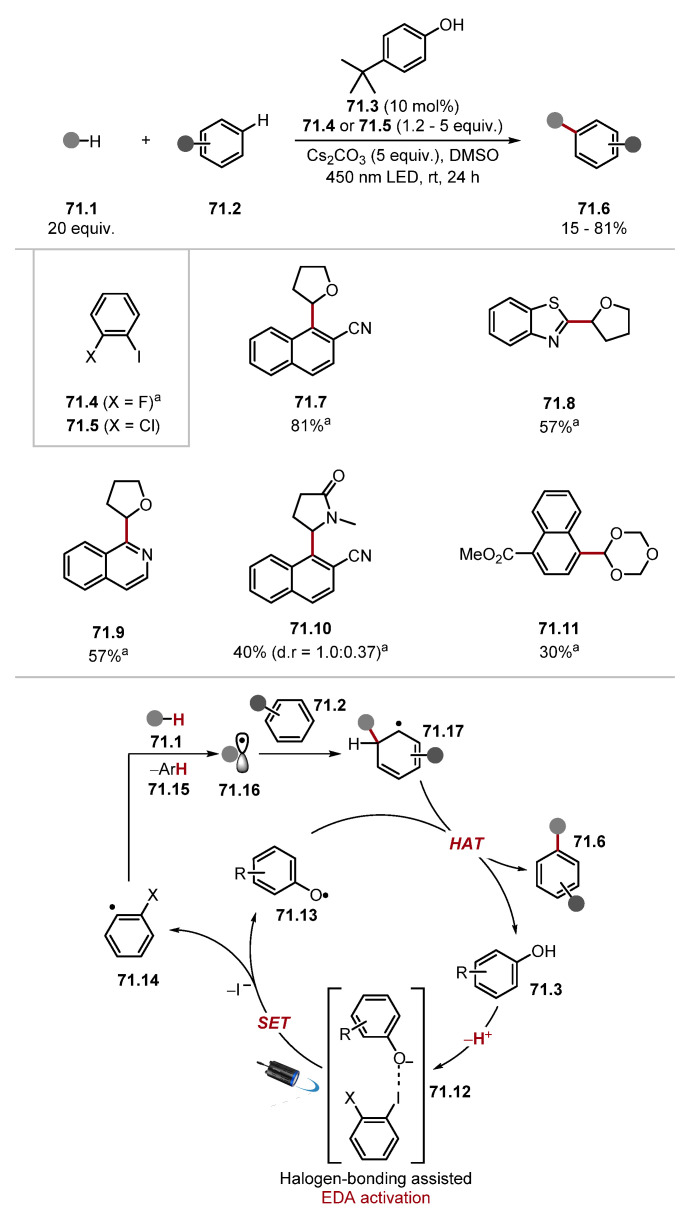
C–H arylation through halogen-bond-assisted EDA activation to form aryl radicals for HAT. ^a^ Aryl iodide **71.4** was used as a HAT reagent precursor.

In 2022, Akiyama developed another EDA complex strategy to access aryl radicals for the oxidation of C–H bonds in **71.1** using 4-*tert*-butylphenol **71.3** and aryl iodides **71.4** or **71.5** (Scheme 71) [347]. Electron-poor arenes **71.2** were used as radical traps in a base-promoted homolytic substitution [348,349,350]. Several aromatic compounds were functionalised, including cyanoarene (**71.7**), benzothiazole (**71.8**), and isoquinoline (**71.9**). Several C–H substrates were also amenable to this protocol, such as ethers (**71.7**, **71.8**, **71.9**), acetals, amide (**71.10)**, and trioxane (**71.11**). Trioxane **71.11** was deprotected to the corresponding aldehyde in a 96% yield constituting a two-step formylation of a C–H bond. The aryl radical **71.14** was generated by the irradiation of a halogen-bond-assisted EDA complex **71.12** with blue LEDs [351]. The aryl radical **71.14** can oxidise the C–H bond in **71.1** to form an alkyl radical **71.16**. This radical is trapped by electron-poor arene **71.2** to form radical adduct **71.17**. The deprotonation and SET or HAT of radical adduct **71.17** delivers the desired product **71.6**.

**Scheme 72:**
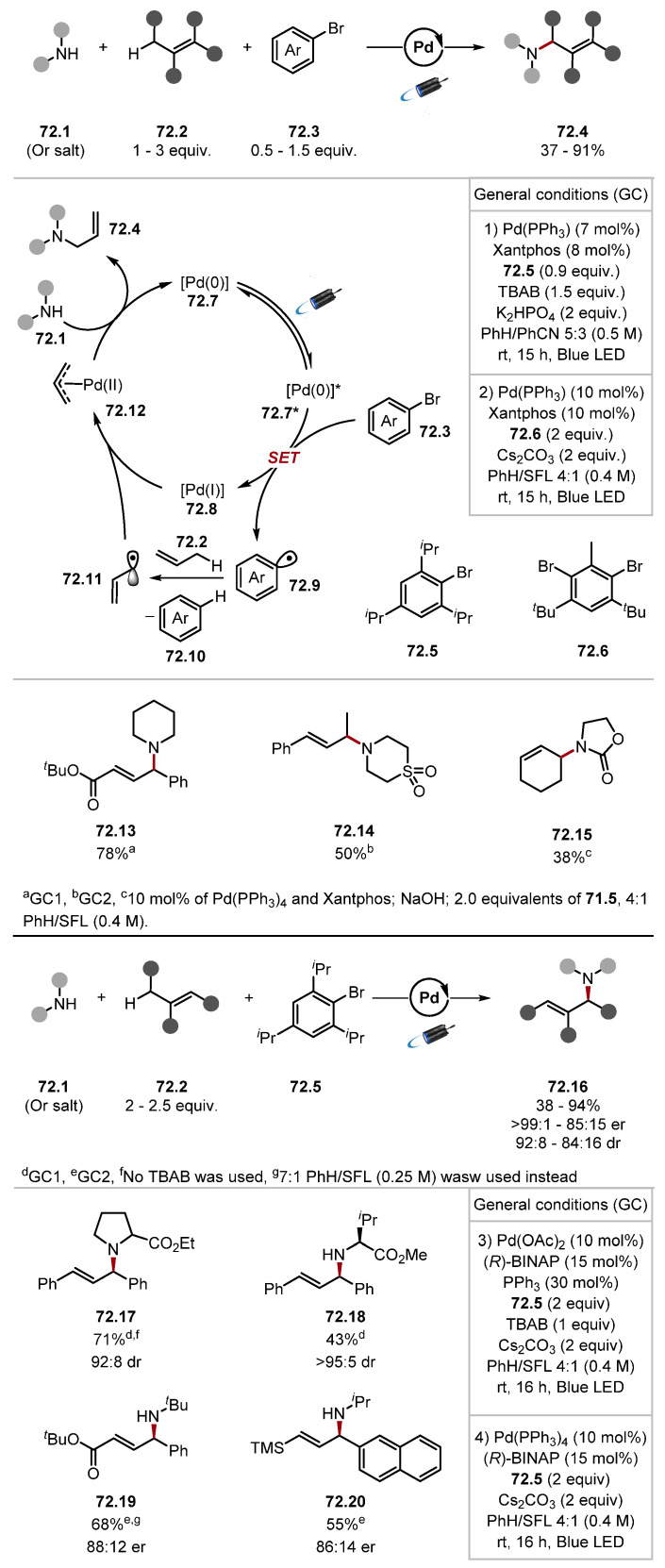
Amination of allylic C–H bonds through HAT with aryl radicals and Pd-catalysis.

In 2023, Gevorgyan developed an allylic C–H amination using the Pd-catalysis in tandem with HAT under blue-light irradiation (Scheme 72) [352]. The oxidative addition of aryl bromide **72.3** with Pd(0) **72.7** under blue-light irradiation results in a formal SET process, which forms Pd(I) **72.8** and aryl radical **72.9**. The aryl radical **72.9** can then abstract an allylic C–H atom from **72.2** to form allyl radical **72.11**. Allyl radical **72.11** combines with Pd(I) **72.8** to form Pd allyl complex **72.12**, which intercepts an amine **72.1** to form the allylic amine product **72.4** and reforms the Pd(0) catalyst **72.7**. Aryl bromides **72.5** and **72.6** were used as aryl radical precursors. Notably, using (*R*)-BINAP as ligand instead of xantphos afforded enantiopure products. The method formed products from a wide variety of amines, including cyclic **72.13** and linear amines **72.18**, as well as protected amino acids **72.17** and **72.18**. Various alkenes were also used, including α,β-unsaturated ester **72.19** and vinyl silane **72.20**.

**Scheme 73:**
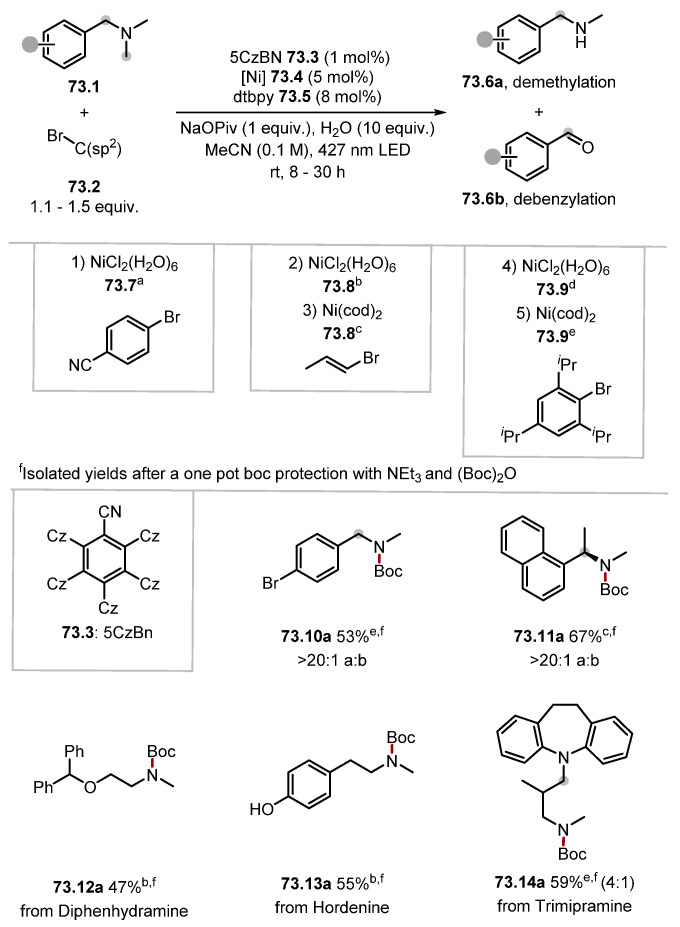
*N*-Demethylation of trialkyl amines using a combination of nickel and photoredox catalysis. ^a^ Conditions 1 were used. ^b^ Conditions 2 were used. ^c^ Conditions 3 were used. ^d^ Conditions 4 were used. ^e^ Conditions 5 were used. ^f^ Isolated yields after a one pot boc protection with NEt_3_ and (Boc)_2_O.

In 2023, Rovis used the expulsion of aryl radicals from a nickel oxidative addition complex for a *N*-demethylation of trialkylamines **73.1** using nickel and photoredox tandem catalysis (Scheme 73) [353]. The reaction occurs through the oxidative addition of Ni(0) to alkenyl/aryl bromide **73.2**. A subsequent anion exchange from nickel bromide to nickel pivalate facilitates the expulsion of an aryl radical upon an energy transfer step involving the photocatalyst. HAT on trialkylamine **73.1** yields an α-amino radical that can be captured with Ni(I) and form an alkyl Ni(II) complex. This complex exists in equilibrium with an Ni(0)-iminium complex. Hydrolysis of this complex delivers the demethylated product and the Ni(0) catalyst. Products were isolated as Boc-protected amines for the ease of isolation. The protocol was showcased on a number of pharmaceuticals containing a trialkylamine motif, forming products such as **73.12a**, **73.13a**, and **73.14a**. The expulsion of an aryl radical from metal centres through LCMT runs parallel to the expulsion of halogen radicals (e.g., Cl**·**, Br**·**) where these radical species have also been used in the context of HAT [31,142,143].

**Scheme 74:**
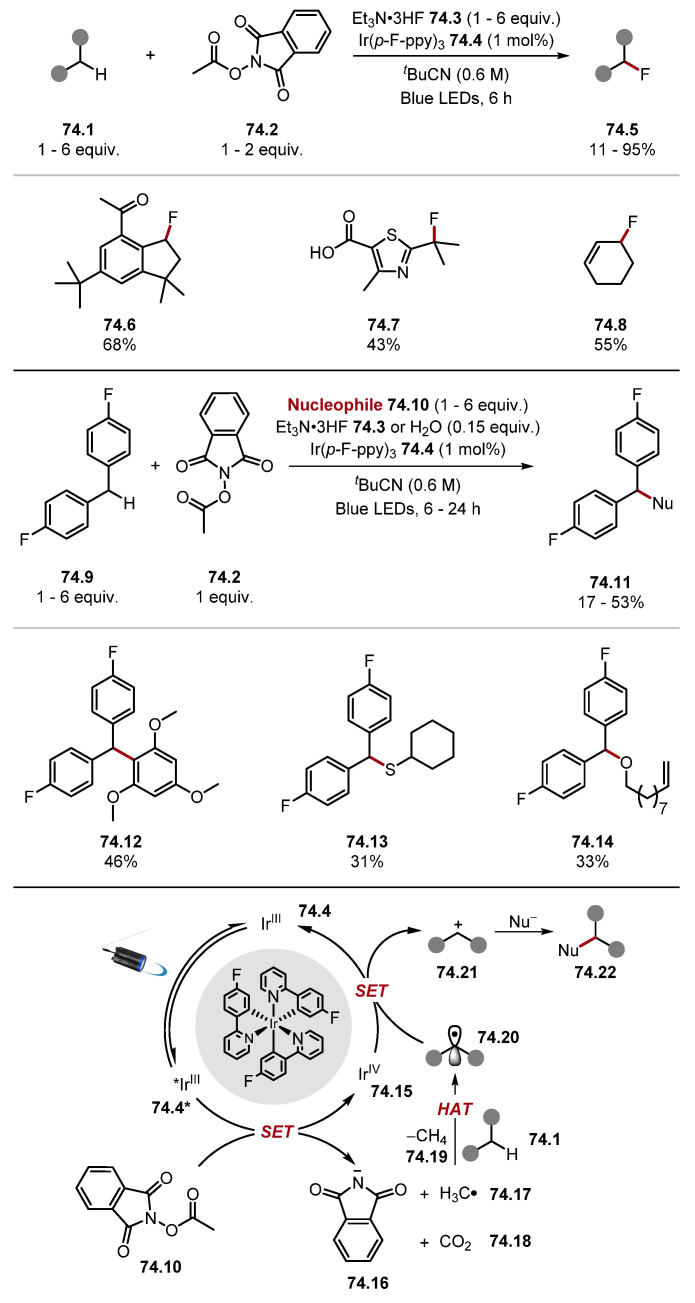
C–H functionalisation through HAT with methyl radical and radical-polar crossover.

In 2021, Doyle used a methyl radical **74.17** for HAT in an oxidative radical polar crossover (ORPC) protocol, which functionalised C–H bonds with nucleophiles (Scheme 74) [117]. The scope of C–H substrates was assessed using Et_3_N.3HF **74.3** as a source of F^−^. Generally, the methyl radical precursor **74.2** was used as the limiting reagent. However, low-to-excellent yields were also achieved by using C–H substrate as a limiting reagent with two equivalents of HAT reagent **74.2** with a 6-fold excess of Et_3_N.3HF **74.3**. The scope of the fluorination was general and displayed a good functional group tolerance with ketones **74.6**, esters, ethers, heterocycles, and even carboxylic acids **74.7** reacting well. Various C–H substrates were used; for instance, alkenes with allylic C–H bonds form products such as **74.8**, while benzylic species form **74.6** and **74.7**. The methyl radical **74.17** also abstracted weak H-atoms adjacent to EWGs such as ketones. Numerous nucleophiles were competent in an adjusted protocol, such as water, electron-rich arenes **74.12**, thiols **74.13**, and alcohols **74.14**. Hammett plot analysis showed HAT with the methyl radical **74.17** was less affected by polar effects than a methoxyl radical, explaining the abstraction of hydrogen atoms adjacent to EWGs preferably. Notably, there has been plenty of historical debate around the philicity of a methyl radical [86]. However, it should be noted that C-centred radicals are not as adept at abstracting protic hydrogen atoms as their isoelectronic amino–boryl radical counterparts as they possess less nucleophilic character [321].

The mechanism proceeds by oxidative quenching of the photoexcited Ir(*p*-F-ppy)_3_ **74.4** (E_1/2_ [Ir^IV^/*Ir^III^] = −1.60 V versus SCE in MeCN) [354] with (**74.2** E_1/2_ = −1.24 V versus SCE in DMF) [117]. The resulting radical anion rapidly undergoes mesolytic cleavage forming phthalimide anion **74.16**, CO_2_, and methyl radical **74.17** [355]. The methyl radical **74.17** then abstracts a hydrogen atom from C–H substrate **74.1** [diphenylmethane BDE_C–H_ = 85.3 kcal/mol] to form an alkyl radical **74.20** and methane **74.19** [methane BDE_C–H_ = 105.0 kcal/mol] [79]. The alkyl radical **74.20** is then oxidised with Ir(*p*-F-ppy)_3_ **74.15** (E_1/2_ (Ir^IV^/Ir^III^) = +0.97 V versus SCE in MeCN) [354], forming carbocation **74.21**, which traps a nucleophile forming the desired product **74.22**.

#### 2.4.2. Boron HAT Reagents

**Scheme 75:**
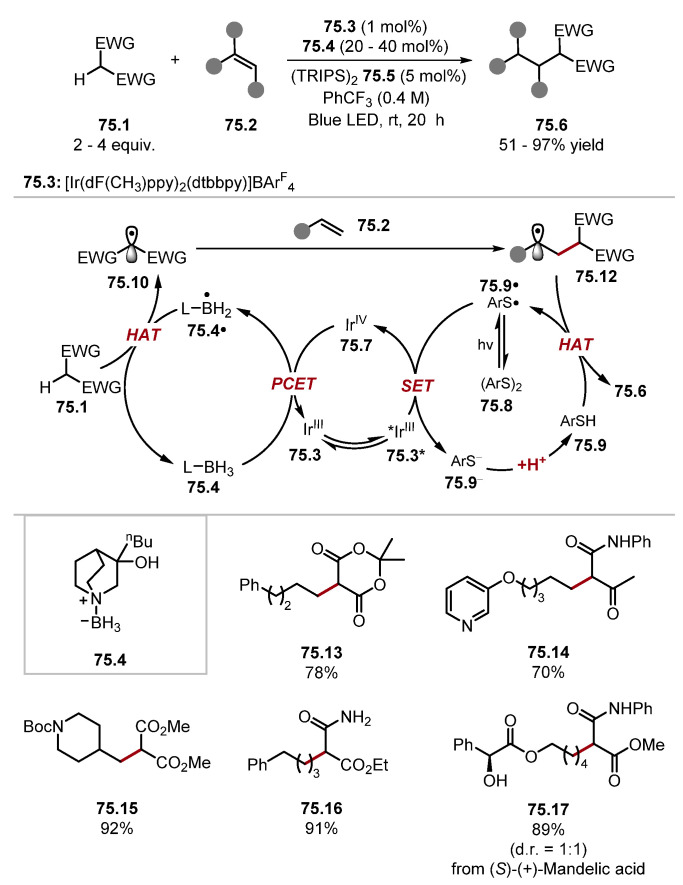
HAT for abstraction of protic hydrogen atoms with amino-borane **75.4** and subsequent hydroalkylation of unactivated olefins.

Chemistry involving boron-centred radicals (boryl radicals) has been popular in recent years [356]. In terms of HAT chemistry, boryl radicals have been used for the selective abstraction of protic H-atoms, making them very unique [84]. Hence, the application of modern technologies of radical generation (photoredox and electrochemistry) to boron-centred radicals are welcome additions in the literature. In 2022, Ye reported the selective abstraction of protic hydrogen atoms from 1,3-dicarbonyls and similar species **75.1** to generate electrophilic radicals for the hydroalkylation of electron-neutral olefins **75.2** (Scheme 75) [357]. This protocol represents an umpolung of classic enol reactivity as species like Meldrum’s acid and dimethyl malonate in products **75.13** and **75.15** generate electrophilic radicals upon HAT. As mentioned previously, the abstraction of protic hydrogen atoms is known to occur with boryl radicals [84]. Amino-borane **75.4** was found to generate a boryl radical **75.4·** upon PCET with a photoredox catalyst **75.3**. The boryl radical **75.4·** selectively abstracted a protic hydrogen from compound **75.1** [**75.4** BDE_B–H_ = 100 kcal/mol versus dimethyl malonate BDE_C–H_ = 93.9 kcal/mol]. This generates an electrophilic alkyl radical **75.10**, which undergoes radical addition with electron-neutral olefin **75.2** to form radical adduct **75.12**. The radical adduct **75.12** undergoes HAT with thiophenol **75.9** to deliver product **75.6**. Various 1,3-dicarbonyl, and related species, including Meldrum’s acid (**75.13**), acetoacetanilide (**75.14**), dimethylmalonate (**75.15**), and amide (**75.16**) reacted in excellent yields. Primary and secondary amides are problematic in classic enolate chemistry due to the acidity of amide protons [358,359,360]. The olefin substrate was also able to accommodate a wide variety of functionality, including pyridine (**75.14**), Boc-piperidine (**75.15**), halogens, and tosylates. The method was also showcased on numerous drugs and complex molecules. This work is a landmark for HAT chemistry as the radical reactivity of enols demonstrated here represents a unique opportunity for the expansion of classic enol C–H functionalisation into the radical sphere. Other methods for generating electrophilic radicals require pre-functionalised substrates (e.g., α-haloketones) or involve the oxidation of enolates [24,361,362,363,364]. Consequently, methods that proceed through HAT of protic hydrogen atoms are of interest for decreasing step counts, improving chemoselectivity and atom economy (oxidative enolate coupling requires stoichiometric amounts of oxidants). Wu recently reported HAT of protic hydrogen atoms in acetonitrile was a competing pathway in a reaction driven by halogen atom transfer with a tertiary amine borane complex [365].

## 3. Conclusions and Closing Remarks

As demonstrated in this review, the field of HAT has experienced tremendous growth due to facile access to radical species enabled by photoredox catalysis and electrochemistry. The increasing understanding of mechanistic intricacies in HAT chemistry has enabled the design of increasingly selective protocols. This can be seen, for example, in fine-tuning the electron density of C–H bonds with additives, as well as the methodical selection and alterations made to HAT reagents. This trend is likely to continue in the coming years with increasingly selective and efficient protocols for HAT chemistry becoming available. The selective functionalisation of specific bonds in saturated heterocycles (for instance, β/γ-C–H bonds in piperidine or β-C–H bonds in pyrrolidine) would constitute a notable milestone in HAT chemistry. This challenge has been addressed with direct HAT using decatungstate [59]. However, indirect HAT methods are limited to one example [294]. Another current challenge is the use of a C–H substrate as the limiting reagent to make HAT protocols viable in LSF, although notable recent advances have been made in this area (Section 2.1.2 and Section 2.3.1). Finally, the introduction of stereocentres at C–H bonds is an outstanding challenge that has received attention in recent years [50,302,303]. The parallel developments in photoredox catalysis, electrochemistry, or other methods of radical generation will enable further development of HAT chemistry. A great example of this is the use of EDA initiation in tandem with HAT chemistry (Section 2.2.2 and Section 2.4.1). In our opinion, the end goal of C–H functionalisation is for specific C–H bonds to be acknowledged as functional handles and widely used as such. In this sense, the wide range of radical traps available, including metal catalysts, and the body of HAT reagents to oxidise them will allow an ever-increasing range of functional groups to be incorporated into C–H bonds.

## Data Availability

Not applicable.

## Abbreviations

(TRIPS)_2_	Bis(2,4,6-triisopropylphenyl) disulfide
4CzIPN	1,2,3,5-Tetrakis(carbazol-9-yl)-4,6-dicyanobenzene
Acr	Acridinium
Ar	Aryl
Bn	Benzyl
Boc	*tert*-butyloxycarbonyl
BOX	Bis(oxazoline) (ligands)
Bz	Benzoyl
CAD	Catalytic Acceptorless Dehydrogenation
Cbz	Carboxybenzyl
CT	Chain transfer
Cz	Carbazolyl
DABCO	Diazabicyclooctane
DCE	1,2-Dichloroethane
DFT	Density-functional theory
DMA	Dimethylacetamide
DMF	*N*,*N*-Dimethylformamide
DMSO	Dimethyl sulfoxide
dr	Diastereomeric ratio
Dtbbpy	Di–*tert*-butylbipyridyl
EAC	Electron acceptor catalyst
EDA	Electron–donor–acceptor
ee	Enantiomeric excess
er	Enantiomeric ratio
EWG	Electron-withdrawing group
HAT	Hydrogen atom transfer
HFIP	Hexafluoroisopropanol
IBX	2-Iodoxybenzoic acid
*^i^*Pr	Isopropyl
LED	Light-emitting diode
LSF	Late-stage functionalisation
MesAcr	Mesityl acridinium
MLCT	Metal–ligand charge transfer
OA	Oxidation addition
PCET	Proton-coupled electron transfer
PFTB	Perfluoro-*tert*-butanol
PRC	Polarity reversal catalysis
PTH	*N-*phenylphenothiazine
Pyf	Tetrafluoropyridinyl
RE	Reductive elimination
SCE	Saturated calomel electrode
SCS	Spin-centred-shift
SET	Single electron transfer
SFL	Sulfolane
SOMO	Singly occupied molecular orbital
TBAB	Tetrabutylammonium bromide
*^t^*Bu	*tert*-butyl
TEDA^2+^	Selectfluor Radical Dication
TIPS	Triisopropylsilane
TMS	Trimethylsilyl
TMS	Trimethylsilyl group
TPI	Thiophosphoric imide
